# Atomic transistors based on seamless lateral metal-semiconductor junctions with a sub-1-nm transfer length

**DOI:** 10.1038/s41467-022-32582-9

**Published:** 2022-08-22

**Authors:** Seunguk Song, Aram Yoon, Jong-Kwon Ha, Jihoon Yang, Sora Jang, Chloe Leblanc, Jaewon Wang, Yeoseon Sim, Deep Jariwala, Seung Kyu Min, Zonghoon Lee, Soon-Yong Kwon

**Affiliations:** 1 Department of Materials Science and Engineering & Center for Future Semiconductor Technology (FUST), Ulsan National Institute of Science and Technology (UNIST), Ulsan, 44919 Republic of Korea; 2 Department of Electrical and Systems Engineering, University of Pennsylvania, Philadelphia, PA 19104 USA; 3 Center for Multidimensional Carbon Materials (CMCM), Institute for Basic Science (IBS), Ulsan, 44919 Republic of Korea; 4 Department of Chemistry, Ulsan National Institute of Science and Technology (UNIST), Ulsan, 44919 Republic of Korea

**Keywords:** Electronic devices, Two-dimensional materials

## Abstract

The edge-to-edge connected metal-semiconductor junction (MSJ) for two-dimensional (2D) transistors has the potential to reduce the contact length while improving the performance of the devices. However, typical 2D materials are thermally and chemically unstable, which impedes the reproducible achievement of high-quality edge contacts. Here we present a scalable synthetic strategy to fabricate low-resistance edge contacts to atomic transistors using a thermally stable 2D metal, PtTe_2_. The use of PtTe_2_ as an epitaxial template enables the lateral growth of monolayer MoS_2_ to achieve a PtTe_2_-MoS_2_ MSJ with the thinnest possible, seamless atomic interface. The synthesized lateral heterojunction enables the reduced dimensions of Schottky barriers and enhanced carrier injection compared to counterparts composed of a vertical 3D metal contact. Furthermore, facile position-selected growth of PtTe_2_-MoS_2_ MSJ arrays using conventional lithography can facilitate the design of device layouts with high processability, while providing low contact resistivity and ultrashort transfer length on wafer scales.

## Abstract (web summary)

Edge-to-edge metal-semiconductor junctions have the potential to improve the performance of 2D transistors. Here, the authors report a synthetic strategy to fabricate monolayer MoS_2_-PtTe_2_ heterojunction arrays with sub-1-nm transfer length and enhanced carrier injection compared to vertical 3D metallic contacts.

## Introduction

The scaling of the dimensions of electronic components is essential to increase the density of devices in an integrated chip (IC)^1,2^. To fulfill industrial requirements, the transistors in ICs must have both ultrashort physical lengths of the gate (*L* < ~12 nm) and contact (*L*_c_, which should be smaller than the required tightest metal pitch, ∼16 nm) by 2034^1,3^. In this respect, two-dimensional (2D) van der Waals (vdW) materials have emerged because of their higher carrier mobility and superior electrostatic controllability at the atomically thin limit^1,2^ to achieve channel scaling for future nanoelectronics. However, fundamental limitations in producing ultra-scaled, low-resistance contact electrodes for 2D semiconductors (e.g., transition metal dichalcogenides (TMDs))^2,4^ using a conventional 3D metal contact limit the switching performance of transistor. For example, the deposition of the 3D metal contact onto 2D semiconducting TMDs typically yields a disorder-rich interface at the metal-semiconductor junction (MSJ), resulting in high contact resistance (*R*_c_)^2,4^. Furthermore, the resultant 3D/2D MSJs have inevitably long transfer length (*L*_T_) of charge carriers (e.g., *L*_T_ > ∼50–200 nm)^5–10^, which leads to an exponential increase in *R*_c_ with the reduction in the contact size if *L*_c_ is smaller than *L*_T_ (i.e., *L*_c_ < *L*_T_), prohibiting prospective contact scaling.

To avoid the problems associated with conventional vertical contact, pioneering works on the use of edge-to-edge connected lateral 3D-2D (or 2D-2D) MSJs have been conducted^5,11–17^. It has a directly metalized junction with strong hybridization, and the absence of atomic discontinuities or defects at the lateral MSJs facilitates superior contact between the 2D semiconducting TMDs and metal electrodes (i.e., smaller *R*_c_)^5,11,13^. Furthermore, using a lateral MSJ has a significant advantage in lowering the *L*_T_ because the carriers are only injected through the few-atom-thick interface (i.e., *L*_T_ decreases to a few nm). Thus, the lateral MSJ-based transistor performs excellently even if *L*_c_ is reduced to the sub-nm range^5^. However, owing to the general thermal and chemical instability of TMDs, practical techniques for controlling heterogeneous integration are highly challenging. Only proof-of-concept has been demonstrated^5,11,13–17^, thereby failing to achieve high yield and high device density. For instance, obtaining edge contact by lithographic techniques requires that the 2D semiconductors be protected against oxidizing conditions using a passivation layer (e.g., hexagonal boron nitride)^11,13^, or in-situ etching prior to edge metallization^5^.

Chemical vapor deposition (CVD) of TMD-based 2D-2D MSJ heterostructures lacks the control of spatial locations^12,14,15^, and their active layer is inevitably degraded by thermal budget effect because the synthesis of 2D semiconductors along the edge of 2D vdW metals are still at the embryonic stage of development. Although graphene has been widely studied for low-resistance contacts with 2D semiconductors^16,17^, the large lattice mismatch between graphene and the 2D semiconductor, along with the polymer-based residues incorporated into the assemblies during the graphene-transfer processes, commonly hinder the production of edge-contacted in-plane 2D-2D MSJs and limit the performance of the contacts^16,18^. Recent studies have demonstrated significant advances towards fabricating lateral 2D-2D MSJs using irregularly-formed flakes of 2D vdW metals, Mo_2_C and VS_2_^14,15^. However, for the approach to be practical and scalable, limitations in terms of the reproducibility of conformal MSJ patterns with sub-1-nm *L*_T_ on a large scale and low *R*_c_ through the suggested approach need to be addressed. Therefore, in the long term, it is vital to develop a groundbreaking technique for fabricating a 2D vdW metal with high stability and processability that can offer a substantial degree of freedom in the device architecture.

Here, we demonstrate the formation of synthetic edge contacts consisting of metallic 2D vdW PtTe_2_ crystals with high thermal stability and the facile position-selected growth of conformal lateral 2D-2D MSJ patterns using conventional lithography. We investigated the high thermal stability of PtTe_2_ under ultrahigh vacuum (UHV) (which maintained its intrinsic surface property up to ∼825 °C) and succeeded in obtaining edge-directed PtTe_2_-MoS_2_ lateral heterojunctions by using two-step CVD. The resultant epitaxially grown lateral MSJ sustained almost ideal stoichiometry without the development of thermally induced voids or the production of mixed alloys. The monolayer MoS_2_ MSJ transistors with PtTe_2_ edge contacts exhibited superior *n*-type carrier transport compared to those with 3D vertical contacts, attributed to both the reduced thermionic emission at the Schottky barrier and the lack of interfacial defects. Additionally, the position-controlled growth of PtTe_2_ and subsequent chemical assembly to MoS_2_ allowed us to obtain 2D TMD-based synthetic edge contact arrays on a large scale. The patterned MSJ showed ultralow contact resistivity (> 11.7 Ω·μm^2^), which is almost one order of magnitude smaller than those of typical 3D top contact electrodes (∼10^3^ to 10^5^ Ω·μm^2^), along with a significantly short *L*_T_ (∼0.7 nm), indicating the potential of PtTe_2_ for affording drastically miniaturized high-quality metal contacts to atomic transistors.

## Results

### Synthetic strategy for high-quality, edge-contact MSJ

**Fig. 1:**
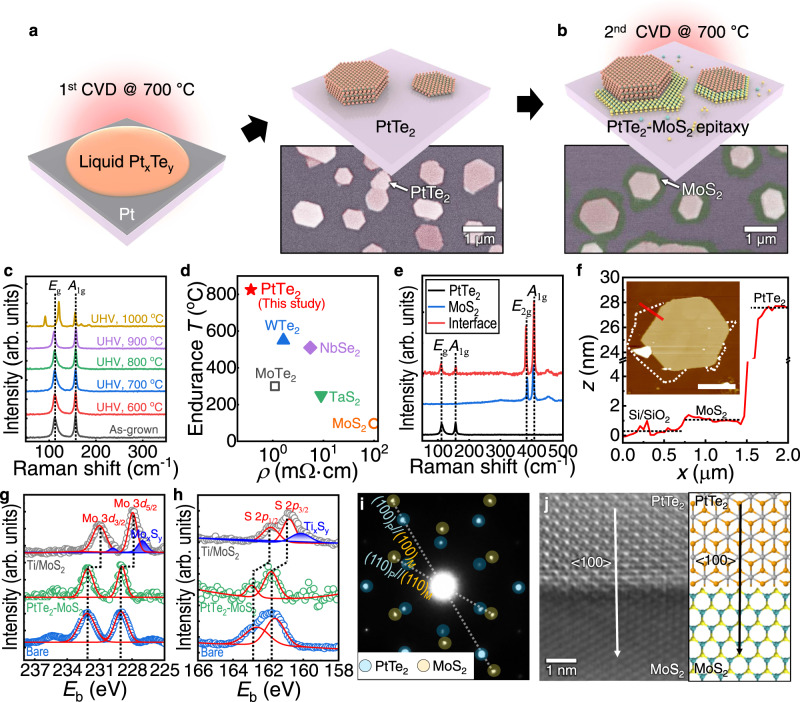
Formation of MoS_2_–PtTe_2_ lateral heterostructure by two-step growth. **a** Schematic of the growth process for the PtTe_2_ flakes at a growth temperature of 700 °C. **b** Schematic of the growth process of MoS_2_ along the edge of PtTe_2_. The insets of (**a**) and (**b**) show the representative false-colored scanning electron microscopy (SEM) image of the synthesized PtTe_2_ flakes and PtTe_2_-MoS_2_ heterostructures on SiO_2_/Si substrate. **c** Raman spectra of multilayer PtTe_2_ flakes displaying strong *E*_g_ and *A*_1g_ signals (vertical dashed lines) without any substantial differences up to 900 °C annealing under UHV conditions. **d** Benchmark plots of metallic TMDs such as 1T’-WTe_2_^31,35^, 2H-NbSe_2_^32,37^, 1T’-MoTe_2_^33,38^, 1T-TaS_2_^36,39^, and 1T’-MoS_2_^34^ with respect to the room-temperature electrical resistivity (*ρ*) and the endurance temperature, *T*, that the material can tolerate without structural degradation. The endurance *T* of the metallic TMDs were characterized under vacuum (solid) or Ar atmosphere (binned). **e** Confocal Raman spectrum captured at the heterojunction’s interface, PtTe_2_, and MoS_2_ flakes. The vertical lines indicate *E*_g_ and *A*_1g_ modes from PtTe_2_, and *E*_2g_ and *A*_1g_ modes from MoS_2_. **f** Atomic force microscopy (AFM) height profile along the red line displayed in the corresponding AFM image (inset, with a scale bar of 2.5 μm; the MoS_2_ boundary is indicated as white dashed lines.), indicating the synthesized MoS_2_ forms a monolayer (*H* ≈ 0.78 nm) while the PtTe_2_ forms multiple layers (thickness, *H* ≈ 28 nm). **g**, **h** X-ray photoelectron spectroscopy (XPS) analysis of the PtTe_2_-MoS_2_ heterostructure. For comparison, the spectra of bare MoS_2_ grown independently on the substrate and those of Ti-deposited MoS_2_ are displayed. The XPS profiles of the for Mo 3*d*, and S 2*p* regions are shown in (**g**) and (**h**), respectively. The XPS Mo 3*d*_3/2_, Mo 3*d*_5/2_, S 2*p*_1/2_, and S 2*p*_2/3_ peaks are specified by dashed lines. **i**, **j** Transmission electron microscopy (TEM) investigations of the heterostructure consisting of a multilayer PtTe_2_ with a monolayer MoS_2_. **i** Selected area electron diffraction (SAED) patterns of the MoS_2_-PtTe_2_ heterostructure, showing the orientationally aligned (100) and (110) planes of each material, indicating the epitaxial growth of the MoS_2_. **j** (left) High-angle annular dark-field scanning-TEM (HAADF-STEM) image demonstrating the atomic arrangements at the MoS_2_-PtTe_2_ heterojunction. (right) Schematic of the lateral heteroepitaxy aligned to the <100> direction.

To produce electronic-grade edge contacts, we developed a synthesis method for lateral MSJs that ensures high-quality MoS_2_ as a channel material. Many previous studies on lateral MSJs prepared using CVD rely on the growth of 2D metals after the preparation of 2D semiconductors because typical 2D semiconductors (e.g., WS_2_, MoSe_2_) require a relatively high growth temperature (∼700–800 °C) compared to vdW metals (e.g., ∼600 °C for NbS_2_ or VSe_2_)^19,20^. However, this sequence for two-step CVD can thermal degrade 2D semiconductors, substantially lowering the performance of resultant device because of the increased chalcogen vacancies of the channel^21^. Instead, we developed a technique to create a 2D semiconductor (i.e., MoS_2_) after preparing 2D metal (i.e., PtTe_2_) to produce a high-quality semiconducting 2D sheet in the lateral MSJ (Fig. 1a, b).

As an efficient edge contact metal for MoS_2_, we selected one of 2D metallic TMDs, PtTe_2_, because it exhibits many attractive features as potential *n*-type metal contacts for 2D semiconductors. For example, the theoretical work function (WF) of few-layered PtTe_2_ (WF; ∼4.56–4.57 eV)^22^ is one of the smallest among those reported for chemically synthesizable metallic TMDs^23–25^ (Supplementary Fig. 1), and its electrical conductivity (>10^6^ S/m) has shown to be the highest among 2D metals^26,27^. However, the simultaneous achievement of large-area production of high-quality stoichiometric, metallic TMD thin films with thermal stability remains challenging, mainly because of the difficulties in incorporating Te and low environmental stability during thermal CVD^28,29^. Furthermore, guaranteeing the thermal stability of the 2D metal is essential for our two-step CVD process because PtTe_2_ must resist the thermal procedure required for synthesizing of MoS_2_ while maintaining excellent quality. In this regard, no success has been achieved yet for 2D-2D lateral heterojunctions based on 2D tellurides.

Since Te vacancies substantially reduce the Gibbs free energy for the adsorption of ambient gas and cause structural instability^30^, the growth of high-quality PtTe_2_ is essential to ensure its stability to some extent. Thus, we synthesized the PtTe_2_ crystals using the eutectic solidification method^26,31^, where the pre-deposited Pt precursor reacted with liquefied Te at 700 °C (Fig. 1a). The resultant PtTe_2_ formed single crystals with a highly stoichiometric nature (Supplementary Fig. 2). As our synthetic methods do not require an oxide precursor, no oxygen-metal bonds, which often become reactive sites and cause poor stability, were formed^32^. In addition, the PtTe_2_ crystals maintained their high-quality structures with Te terminations even after exposure to air for up to ∼3 h, as indicated by TEM analysis (Supplementary Fig. 3). The WF of PtTe_2_ (∼4.65 eV), characterized by ultraviolet photoelectron spectroscopy (UPS), was comparable to the computed value^22^ (Supplementary Fig. 2h), satisfying the basic requirement of an efficient *n*-type contact for the MoS_2_ transistor, considering its band structure (Supplementary Fig. 1).

To evaluate the thermal stability of the as-synthesized PtTe_2_, the crystal was annealed under UHV conditions (∼10^−10^ Torr) at temperatures between *T* = 500–1000 °C for 1 h. The Raman spectra obtained are shown in Fig. 1c. The as-grown PtTe_2_ and the UHV-annealed crystals heated to 900 °C showed comparable Raman in-plane *E*_g_ (∼110 cm^−1^) and out-of-plane *A*_1g_ (∼156 cm^−1^) vibrational modes of PtTe_2_^27^ (black dashed line in Fig. 1c). Remarkably, only annealing above ∼1000 °C altered the peak positions and their intensities, demonstrating the high thermal stability of the PtTe_2_. The thermal stability is an intrinsic property independent of the thickness and degree of structure order, as evaluated by X-ray photoelectron spectrum (XPS) and UPS (Supplementary Fig. 4). The surface properties of PtTe_2_ start to change above the *T* of ∼825 °C, which is far beyond the limitations of metallic TMDs (e.g., WTe_2_, MoTe_2_, TaS_2_, and 1 T’-MoS_2_) measured under vacuum or in an Ar atmosphere^32–36^. In addition, low electrical resistivity of PtTe_2_ (*ρ* ≈ 0.37 mΩ·cm; Supplementary Fig. 5a, b), even lower than other few-layered metallic TMDs^31,34,37–39^ (*ρ* ≈ 0.5–100 mΩ·cm), shows promise for a robust electrode that may prevent thermal stress as summarized in Fig. 1d.

Following the synthesis of thermal-stable PtTe_2_ multilayers, a monolayer of MoS_2_ was formed laterally along the edge of the PtTe_2_ by CVD at ∼700 °C (Fig. 1b). Compared to the edge, the atomically pristine, dangling-bond-free surface of a 2D crystal typically possesses fewer surface defects and minimal MoS_2_ nucleation. Density functional theory (DFT) simulations were performed to investigate the selective nucleation of MoS_2_ on the edges of PtTe_2_. Adsorption energy calculations reveal that there was preferential adsorption and subsequent nucleation of MoS_2_ at the PtTe_2_ edge. For instance, the MoS_2_ monomer exhibited a lower adsorption energy of −3.6 eV at the PtTe_2_ edge compared to −2.7 eV on the PtTe_2_ basal plane (Supplementary Fig. 6). These findings are consistent with those of previous reports on the two-step CVD of 2D heterostructures, where in the adsorption energy of 2D TMDs at the edge of a 2D crystal was lower than that on the 2D basal plane^19,40^. The low occurrence of MoS_2_ nucleation on the PtTe_2_ surface is attributed to the higher adsorption energy of MoS_2_ nuclei on the basal plane. In our experiments, we significantly lowered the mass flux of precursors through the source-contact geometry and use of a MoO_x_ thin-film precursor significantly reduced the opportunities for nucleation (Supplementary Note 1). The attachment of adatoms or atomic clusters of MoS_2_ by heterogeneous nucleation was allowed only at the edge of PtTe_2_. The introduction of a higher mass flux would thus increase the possibility of producing more nucleations^41^ and trigger the synthesis of randomly distributed multilayer MoS_2_ on the structures.

Attributed to the edge-mediated growth mode, the MoS_2_ crystals were observed exclusively along the PtTe_2_ edges as shown in false-colored scanning electron microscopy (SEM) images (inset of Fig. 1b and Supplementary Fig. 7). The Raman signals at the interface of MSJ demonstrated the strong signals of PtTe_2_ without variation in positions, while the MoS_2_ features of E_2g_ (∼384 cm^−1^) and A_1g_ (∼407 cm^−1^) modes also existed (Fig. 1e). Most of the MoS_2_ attached to PtTe_2_ was a monolayer with a uniform thickness of ∼0.7 nm, as shown in the AFM image and height profile (Fig. 1f). XPS analysis of the heterostructures was used to reveal the surface compositions and chemistries (Fig. 1g, h and Supplementary Fig. 8). The XPS spectra of the MoS_2_ layer did not show a substantial peak shift compared to that of non-stitched bare flakes (green, Fig. 1g, h). In comparison, the vertically deposited 3D metals on MoS_2_ can induce surface defects^4,42^, that is, a non-stoichiometric layer (i.e., Mo_x_S_y_) or alloys (i.e., Ti_x_S_y_), as we experimentally demonstrated in the case of Ti/MoS_2_ (gray, Fig. 1g). Hence, the coincident binding energy of MoS_2_ connected to PtTe_2_ and that of the bare flakes suggests that the present growth mode conferred the intrinsic surface properties of the 2D semiconductor layer (i.e., MoS_2_), as the MoS_2_ layer was grown after preparing PtTe_2_.

XPS analysis further revealed no noticeable change in the stoichiometry of PtTe_2_ after thermal CVD for MoS_2_ (at. % (Te/Pt) ≈ 1.80). This high stability of PtTe_2_ during thermal CVD was also confirmed by SEM-EDS characterization (Supplementary Fig. 7d). Pt and Te were the only two observed elements, and there were no apparent variations in their stoichiometry (that is, averaged at. % (Te/Pt) = 1.90 ± 0.06). In addition, we electrically characterized PtTe_2_ to verify that the influence of the high-temperature process was negligible (Supplementary Fig. 5c, d). The sustained low *ρ* (≈ 0.35 mΩ·cm) and the weak dependence on gate voltage (*V*_g_) imply that exposure to chemical species (MoO_x_ and S) does not degrade PtTe_2_ (Supplementary Fig. 5d). Back-scattered SEM analysis did not show any traces of in-plane mixed alloying (Supplementary Fig. 7e–h), further demonstrating the high stability of PtTe_2_.

Structural analysis conducted using transmission electron microscopy (TEM) also confirmed the high quality of the PtTe_2_-MoS_2_ lateral heterostructure (Fig. 1i, j, and Supplementary Figs. 9–11). We found that the MoS_2_ monolayer grew epitaxially along the edge of PtTe_2_ single crystal using the selected area electron diffraction (SAED) pattern (Fig. 1i), indicated by the two aligned sets of hexagonal diffraction spots with six-fold symmetry reflecting the epitaxially aligned (110) and (100) lattice planes. Atomic-resolution analysis was performed using high-angle annular dark-field scanning-TEM (HAADF-STEM) (Fig. 1j). The 2H-MoS_2_ was atomically stitched to 1T-PtTe_2_ without void-like defects. The high stoichiometry and compositional consistency of PtTe_2_ were also confirmed by the STEM-EDS mapping and spectra (Supplementary Fig. 9a–f). The electron energy loss spectroscopy (EELS) line scan further revealed the abrupt compositional change at the junction (Supplementary Fig. 9g–i). Cross-sectional TEM analysis of synthetic MSJ also provided identical insight into the edge-to-edge connected structure, demonstrating in-plane epitaxial growth of MoS_2_ and sharp interface at the junction (Supplementary Fig. 10). Remarkably, the absence of mixed or alloyed structures in the as-synthesized MSJ implies that our synthesis approach was successful. This also demonstrates the merit of our approach compared to those adopted in previous studies^14,16,18^ (as summarized in Supplementary Table 1). Interestingly, the high density of dangling bonds at the PtTe_2_ edge generated several MoS_2_ basal planes that were attached vertically to the edges of the PtTe_2_ flakes (Supplementary Fig. 11). However, the vertical MoS_2_ layers were relatively insignificant in the synthesized results because most MSJs possessed laterally stitched components without vertical structures.

We performed DFT calculations to understand the formation mechanism of defect-free interface in the lateral PtTe_2_-MoS_2_ MSJ (Supplementary Fig. 12). The calculated relative energies (*ΔE*) of the possible intermediates (i.e., MoO_x_ and S adsorbents) attached to the PtTe_2_ edge indicated the relative stability of each atomic structure during the epitaxial growth of MoS_2_ (Supplementary Table 2). We found that reactive gas-phase S atoms were bound to MoO_3_ precursor attached to the edge of PtTe_2_ (*ΔE* < −2.88 eV), and that O in MoO_3_ tended to desorb as it reacted with additional S by forming SO_2_(g) because of the consequent exothermic processes. A similar repeated reduction process for MoO_3-x_ resulted in the formation of the most stable MoS_2_ structure because of the substantial energy relative to the initial structure (*ΔE* = −19.49 eV). Furthermore, each periodic interfacial cell comprised four PtTe_2_ and five MoS_2_ unit cells to minimize the lattice mismatch at the heterojunction (Supplementary Fig. 13). The lattice mismatch between PtTe_2_ and MoS_2_ was ∼18% when each unit cell of PtTe_2_ was matched to a unit cell of MoS_2_ in a one-to-one ratio according to the equation *λ* = (|*a*_1_ − *a*_2_ | )/*a*_2_, where *a*_1_ (∼3.25 ± 0.05 Å) and *a*_2_ (∼3.96 ± 0.05 Å) are the in-plane lattice constants of MoS_2_^43^ and PtTe_2_^44^, respectively (Supplementary Fig. 13a). In comparison, adjusting the number of unit cells on the interface to a five-to-four ratio of MoS_2_ and PtTe_2_ resulted in a smaller mismatch between the materials (∼3%). STEM analysis also validated our heterostructure with consistently matched periodic cells along the <100> direction, indicating a (semi-)coherent interface (Supplementary Fig. 13b–d). Given this smaller interfacial cell mismatch (∼3%), we calculated the most stable atomic structure for the heterostructure using DFT and the multicell model, as depicted in Supplementary Fig. 13b, c. The Pt-S and Mo-Te covalent bonds formed at the interface, resulted in strong orbital interactions between the atoms at the heterojunction.

### High-performance monolayer MoS_2_ FETs with PtTe_2_ edge contact

**Fig. 2:**
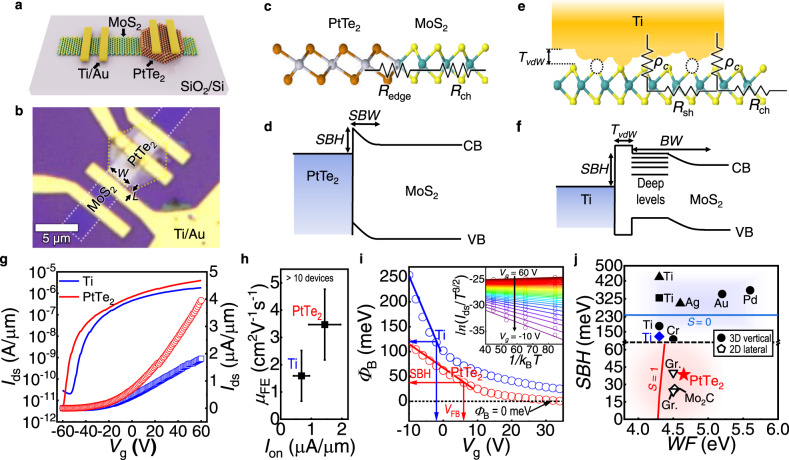
High performance of edge-contacted PtTe_2_-MoS_2_ metal-semiconductor junction (MSJ) field-effect transistors (FETs). **a**–**h** Monolayer MoS_2_ FETs with asymmetric carrier injection from PtTe_2_ lateral epitaxial contact and from vertical Ti, characterized at room temperature. **a** Schematic and (**b**) optical microscopy image of a device with asymmetric Ti/Au and PtTe_2_ electrodes contacted to a monolayer MoS_2_ (white dashed lines) with a defined channel width (*W*) and length (*L*). **c**, **e** Cross-sectional illustrations of resistance networks of monolayer MoS_2_-based MSJ with (**c**) atomically stitched PtTe_2_ lateral contact and (**e**) top-contact with Ti/Au. Metallization of MoS_2_ can degrade both the contact resistivity (*ρ*_c_) and sheet resistance (*R*_sh_) of MoS_2_. In contrast, the PtTe_2_-MoS_2_ heterostructure has simpler resistance components, and the chemically derived edge resistance (*R*_edge_) is the only series resistance for the channel (*R*_ch_). **d**, **f** Comparison of band alignments with different Schottky barrier heights (SBHs) and Schottky barrier width (SBW) with respect to the MoS_2_ conduction band (CB) and valance band (VB) edges for (**d**) PtTe_2_-MoS_2_ heterostructure and (**f**) conventional 3D metal-contacted MoS_2_. Tunneling barrier formed by vdW gap between Ti and MoS_2_ (*T*_vdW_) and defect-induced deep levels are displayed in (**f**). **g** Drain current (*I*_ds_) as a function of gate voltage (*V*_g_) for PtTe_2_-MoS_2_ (red) and Ti/MoS_2_ (blue) MSJ FETs on the logarithmic (lines; left) and linear (symbols; right) scales. **h** Summary of the field-effect mobility (*μ*_FE_) and on-state current (*I*_on_) of FETs with different carrier injections from PtTe_2_ (red) and Ti (blue). The error bars indicate the standard deviations from each device set. **i**, **j** SBH of PtTe_2_-MoS_2_ MSJ extracted using thermionic emission model. **i** Calculated thermionic barrier height of PtTe_2_-MoS_2_ (red) and Ti/MoS_2_ MSJ (blue) as a function of *V*_g_ showing the low SBH of 38.5 meV at the flat band voltage. Inset: representative Arrhenius plot (*ln*(*I*_ds_/*T*^3/2^) *vs*. 1/*k*_B_*T*) of PtTe_2_-MoS_2_ heterojunction with different *V*_g_. **j** Comparison of the SBH of few-layer MoS_2_ based MSJ FETs with 3D top contacts (Ti^47–49^, Cr^48^, Ag^49^, Au^48^, and Pd^48^; solid) and with 2D lateral contacts (Mo_2_C^15^, and graphene^16,17^; open). The extracted SBHs of PtTe_2_-MoS_2_ (red) and Ti/MoS_2_ (blue) are indicated by colored symbols. Plots of the strength of Fermi-level pinning (*S* = |d(SBH)/d(WF)|) are shown as solid lines in order to compare the mechanism of formation of the SBHs.

We now shift our focus to the electrical characterization of the edge-contacted 2D-2D MSJ field-effect transistors (FETs). After the definition of MoS_2_ channels by the reactive ion etching process, PtTe_2_-flake-connected MoS_2_ MSJ FETs were fabricated by Ti/Au (10/70 nm) contact pad deposition on PtTe_2_. The Ti/Au layer was also deposited on the other side of the MoS_2_ channel to produce an asymmetrical contacted MoS_2_ channel with a fixed width (*W*) and length (*L*) for comparison with the MoS_2_-Ti vertical junction (Fig. 2a, b). The gate bias was applied through the 300-nm-thick SiO_2_ dielectric layer for this measurement. Because the reverse-biased contact (source) causes most of the voltage drop and dominates the transistor behavior in *n*-type MoS_2_ MSJ FETs, the source (either Ti or PtTe_2_) determines the performance of a FET with asymmetric contacts. This allows the electrical properties of the barrier to be systemically evaluated by controlling the interface^15,29^. We selected Ti as the counterpart to PtTe_2_ because it is the most widely used contact and has a low WF of ∼4.33 eV^23^, which is even smaller than that of PtTe_2_ (∼4.60-4.65 eV in Fig. 1g).

Figure 2g shows a representative transfer characterization (*I*_ds_*-V*_g_) of the MSJs where the electrons were injected from the PtTe_2_ edge (red) and the vertical Ti contact (blue). In addition to the output curve (Supplementary Fig. 14a), the on-state current (*I*_on_) of PtTe_2_-MoS_2_ (∼4.0 μA/μm) was twice that of Ti-MoS_2_ (∼1.8 μA/μm). The two-terminal field-effect mobility (*μ*_FE_) was also increased to ∼9.7 cm^2^ V^−1^ s^−1^ by injection of charge carriers from PtTe_2_, compared to Ti (∼7.0 cm^2^ V^−1^ s^−1^) for the same channel (∼3.5 ± 1.3 cm^2^ V^−1^ s^−1^ and ∼1.6 ± 0.9 cm^2^ V^−1^ s^−1^ on average for more than 10 devices with PtTe_2_ and Ti asymmetric contacts, respectively; and the averaged values are demonstrated in Fig. 2h). We found a slight negative shift of the turn-on voltage when the PtTe_2_ source electrode was selected. This negative shift reflects the improved carrier injection from the contact^45,46^ because the reduced *R*_c_ and SBH caused the transistor easier to switch on (note that we could not observe an obvious doping effect on the in the XPS (Fig. 1g, h) and Raman spectra (Fig. 1e) after lateral epitaxy of PtTe_2_). The performance enhancement was also confirmed for the MoS_2_ FET with symmetric contact geometry (i.e., PtTe_2_-MoS_2_-PtTe_2_; Supplementary Figs. 14b–e). It showed a *μ*_FE_ of ∼15.8 cm^2^ V^−1^ s^−1^, higher than those from asymmetrically contacted FET, which again verifies the influence of the resistance of the FET components related to Ti.

High-energy deposition of a 3D metal typically degrades the surface of monolayer MoS_2_ by forming sulfur vacancies and promoting atomic diffusion at MSJs^4,42^ (i.e., forming interfacial defects of Ti/MoS_2_ as shown in Fig. 1g, h). These processes increase the *R*_sh_ and contact resistivity (*ρ*_c_) by forming localized states as depicted in the contact components in Fig. 2e, f^29,47–49^. In addition, the established gap states can shift the charge neutrality, pinning the Fermi level to the mid-gap of MoS_2_, which can significantly impact the Schottky barrier height (SBH)^48,50^. According to Sze’s model^51^, the Fermi level pinning (FLP) strength increases with the density of interfacial traps (*D*_it_; Supplementary Fig. 15 and Supplementary Note 2); thus, the large *D*_it_ in our Ti/MoS_2_ MSJ FETs could induce strong FLP to the mid-gap states, and therefore resulted in a large SBH and lower thermionic emission efficiency over the SB. Furthermore, the top contact scheme has a long path for carrier transfer (e.g., wide width of the barriers, BW), leading to a larger *R*_sh_ and *ρ*_c_. The vdW gap between the 3D metal and 2D semiconductor can also produce an additional tunnelling barrier (*T*_vdW_), although Ti/MoS_2_ may possess a narrower *T*_vdW_ than other 3D metals/MoS_2_ because of the strong hybridization^2,42^. In contrast, the PtTe_2_-MoS_2_ MSJ developed herein has a cleaner interface that prevents further metallization on the MoS_2_ surface (i.e., absence of *R*_sh_), leading to simple contact components only related to the edge (Fig. 2c, d)^5,11^. The absence of *T*_vdW_ and the reduced BW can improve carrier injection through the edge of PtTe_2_. Notably, despite the presence of a vdW gap between PtTe_2_ and the metal contact pad (i.e., Ti/Au in Fig. 2a), the contribution of *R*_c_ in the Ti/PtTe_2_ interface (∼0.12–0.23 kΩ·μm) of the MoS_2_-based MSJ system was insignificant (∼0.13%; Supplementary Fig. 16). Furthermore, the estimated *R*_c_ of the Ti/PtTe_2_ interface is almost the lowest value that can be obtained in a 3D metal/vdW metal interfacial system (Supplementary Table 3) and is even smaller than that obtained with the most widely used vdW metal (graphene).

To gain insight into the physical characteristic associated with carrier transport through the barriers, the thermionic barrier heights (*ϕ*_B_) of the monolayer MoS_2_ MSJ FETs with symmetric contacts of PtTe_2_ and Ti were calculated by electrical characterization at low temperatures (Fig. 2i, j and Supplementary Fig. 17). This allowed us to extract the SBHs at the interface by fitting the Arrhenius plot to the thermionic emission model^48^:

1 $${I}_{{ds}}=\left[A{A}^{*}{T}^{3/2}{{\exp }}\left(-\frac{q{\phi }_{B}}{{k}_{B}T}\right)\right]\left[{{\exp }}\left(\frac{q{V}_{{ds}}}{{k}_{B}T}\right)-1\right]$$

Here, *A* is the junction area, and A^*^ is the effective Richardson–Boltzmann constant. According to Eq. (1), the *ϕ*_B_ can be extracted from the slope of the Arrhenius plot (inset of Fig. 2i), resulting in *ϕ*_B_ as a function of *V*_g_ (Fig. 2i). Interestingly, the *ϕ*_B_ for PtTe_2_-contacted MSJ at the flat band voltage (*V*_FB_) was found to be a substantially small value of ∼35.9 ± 9.8 meV (average of four devices), where the primary carrier injection mechanism changed from the thermionic transport (*V*_g_ < *V*_FB_) to tunneling (*V*_g_ > *V*_FB_, where the linear *V*_g_-*ϕ*_B_ relation ends at this actual SBH). For *V*_g_ values exceeding ∼30 V, *ϕ*_B_ was negative, indicating tunneling-dominant electrical transport through the reduced SBW of the edge contact, which is difficult to achieve with the 3D metal contact owing to the wider BW and the presence of *T*_vdW_ for the latter. For example, the SBW at the PtTe_2_-MoS_2_ interface (∼0.94 nm) calculated using a classical model for Schottky diodes was smaller than that of the Ti/MoS_2_ MSJ FETs (∼1.5 nm) (Supplementary Note 2).

The extracted SBH (*ϕ*_B_at *V*_FB_) of ∼35.9 meV is considerably lower than that of the Ti/MoS_2_ MSJ (∼125 meV) and almost one order of magnitude smaller than previously reported values for other 3D-metal/MoS_2_ MSJ^47–49^ (≈100–500 meV in Fig. 2j). Deep energy level states were established primarily because the interface was degraded by penetration of the 3D metals into the MoS_2_ band^29,47–49^ (Fig. 2f). Therefore, the SBH of the FETs with Ti contact was much higher than the ideal value calculated by applying the Schottky-Mott rule (e.g., SBH = WF – *χ* ≈ 0 meV, where *χ* is the electron affinity of monolayer MoS_2_ (≈ 4.28 eV)^48^). The deviation from the Schottky-Mott rule is substantial for 3D contacts; thus, SBHs cannot be effectively modulated by selecting the 3D metals, as the Fermi levels are pinned to the shifted charge-neutral level (CNL). The interface index, *S* (=|d(SBH)/d(WF)|), indicates the extent of the deviation^48^, which approaches zero for the 3D metal contacts (blue line in Fig. 2j). This strong FLP (*S* ≈ 0) agrees well with the theoretical model prediction^51^ that the *S* value decreases with increasing interfacial defect density (*D*_it_ ≈ ∞). We found that the increase in *D*_it_ due to the replacement of the edge PtTe_2_ by a top Ti contact could approach ∼1.13 × 10^13^ eV^−1^ cm^−2^, and gave rise to a stronger FLP (Supplementary Fig. 15 and Supplementary Note 2).

Compared with vertical 3D metal contacts, the synthetic edge contact can weaken the FLP because of the lowered dimensionality with fewer interfacial defects^10,13,50,52^ Hence, the SBHs can follow the ideal Schottky-Mott limit to some extent (*S* = 1; red line in Fig. 2j), enabling the formation of tiny SBHs by using low-WF 2D metals. Furthermore, the impact of strong covalent bonds at the edge interface, which may result in a slight deviation from the perfect Schottky-Mott limit through the formation of a metal-induced gap state (MIGS)^13^, is less significant compared to that at the top contacts because of the 1D-like atomically thin interface (Supplementary Fig. 18). Accordingly, the SBH in all 2D lateral contacts (Mo_2_C^15^, and graphene^16,17^) to few-layer MoS_2_ MSJ FETs produced by CVD (∼26-45 meV) is much smaller than those of vertical 3D contacts (Fig. 2j). Similarly, the FLP-free PtTe_2_ edge interface allowed the achievement of a negligible SBH (∼35.9 meV) due to low WF of PtTe_2_ (∼4.56 eV^22^); this is one of the lowest WF values among 2D metals, as summarized in Supplementary Fig. 1. This smaller SBH (∼35.9 meV) enabled improved thermionic emission and tunneling transport through the SB compared with the top-contact Ti/MoS_2_, which had a higher SBH (∼135 meV) and additional *T*_vdW_. A higher *μ*_FE_ and on/off ratio was therefore achieved, as indicated by variation of carrier transport through the barriers with *V*_g_ (Supplementary Fig. 19).

### Spatial arrangement of synthetic edge contacts

**Fig. 3:**
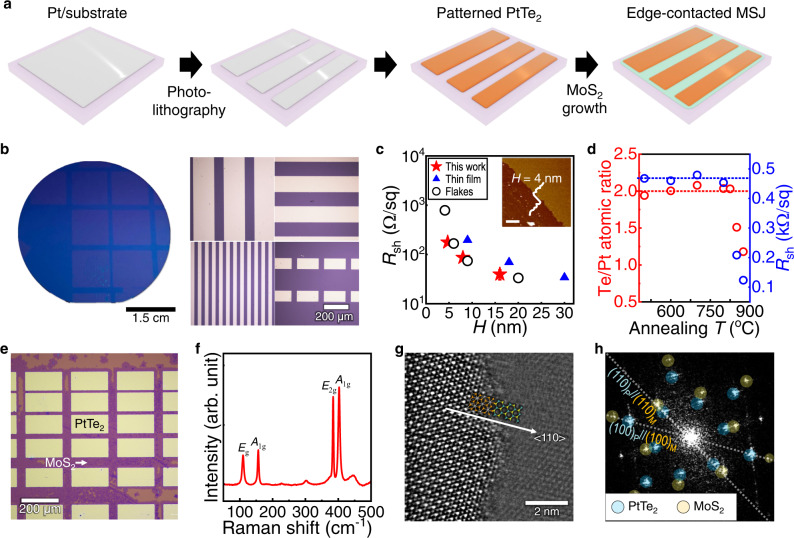
Wafer-scale growth of PtTe_2_ patterns for synthetic edge contact arrays. **a** Schematic of procedure for realizing PtTe_2_-MoS_2_ heterostructure arrays in a position-controllable manner, involving photolithography before the tellurization of Pt. **b** Photograph image of as-grown large-scale PtTe_2_ patterns on a 2-inch-scale SiO_2_/Si substrate (left), and representative OM images of the thin film captured on the left image (right). **c** Sheet resistance (*R*_sh_) of as-grown PtTe_2_ thin film as a function of the film thickness (*H*), characterized by four-probe method. *R*_sh_ values for high-quality PtTe_2_ from previous reports^27,53^ are displayed for comparison. The inset shows a representative AFM image of the as-grown thin film (scale bar: 1 μm). **d** (left) XPS-derived atomic ratio of the thin film, showing the nearly ideal stoichiometry of PtTe_2_ (at. % (Te/Pt) ≈ 2; dashed red line) irrespective to *T* up to 825 °C, (right) *R*_sh_ of thin films as a function of annealing *T*, with *R*_sh_ ~467 Ω/sq (for *T* = 500 °C) indicated with a blue dashed line. **e** Representative OM images of MoS_2_ monolayer laterally contacted to the edge of polycrystalline PtTe_2_ thin film patterns with *H* ≈ 4 nm, in the form of fully stitched thin film depending on the growth conditions. **f** Representative Raman spectrum of PtTe_2_-MoS_2_ heterostructure, showing strong signals of each material, e.g., *E*_g_ (110.2 cm^−1^) and *A*_1g_ (156.5 cm^−1^) peaks of PtTe_2_ and *E*_2g_ (384.5 cm^−1^) and *A*_1g_ (402.3 cm^−1^) modes of MoS_2_. **g**, **h** TEM analysis of heterostructure with patterned PtTe_2_ (*H* ≈ 4 nm) and monolayer MoS_2_. **g** HAADF-STEM image of heterostructure showing epitaxially stitched PtTe_2_ and MoS_2_ without void-like defects along both surfaces. **h** Diffractogram corresponding to interface region, indicating orientationally aligned (100) and (110) planes in each atomic structure.

As the ultimate approach for demonstrating the advantage of PtTe_2_ with respect to material processing, we eventually controlled the spatial arrangement of the synthetic edge contacts at the microscopic level (Fig. 3a). Powder-based tellurization was used to transform the Pt patterns prepared by conventional photolithography into PtTe_2_ patterns at a low temperature of ∼400 °C (here, we used Pt instead of PtO_x_, which can assure high quality of the resultant PtTe_2_^32^). Remarkably, the patterns were successfully manufactured on a 2-inch SiO_2_/Si wafer, which is vital for achieving scalable, mass production of PtTe_2_ electrodes in any desired shapes (Fig. 3b). The electrical characterization by the four-point probe method showed that the *R*_sh_ of the as-synthesized PtTe_2_ thin film was *H*-dependent due to the greater carrier scattering as *H* decreased (Fig. 3c). The *R*_sh_ values of the developed PtTe_2_ samples were lower than those previously reported for CVD-grown films^53^, and comparable to those of single-crystalline flakes^27^, suggesting that the developed films are of high quality. We found that the XPS-extracted stoichiometries for 4-nm-thick PtTe_2_ were almost perfect (at.% ratio of Te/Pt ≈ 2) independent on the thermal stress up to *T* ≈ 825 °C (Fig. 3d), suggesting the high quality and thermal stability of the film (similar to the case for single crystals in Fig. 1). The *R*_sh_ of the PtTe_2_ thin film was also ∼450 Ω/sq for the samples annealed below 800 °C. However, it suddenly changed as *T* reached 850 °C (*R*_sh_ ≈ 208 Ω/sq), indicating the atomic displacements of Te (which begins to resemble the electrical properties of Pt, as noted in caption of Supplementary Fig. 4).

Subsequent conducted thermal CVD of MoS_2_ induces lateral epitaxy of monolayer MoS_2_ from the edge of the arranged PtTe_2_ patterns (Fig. 3e). A key to achieving a fully stitched MoS_2_ thin film between the PtTe_2_ crystals (instead of small flakes) is the extension of the growth time while decreasing the deposition rate via delicate control of the atomic flux. A higher growth temperature (>730 °C) and extensive precursors (for MoO_x_ and S) resulted in multilayer MoS_2_ and alloyed MoS_2_/PtTe_2_ structures due to the island growth mode, whereas the layer-by-layer growth mode was enabled by the opposite manner. Figure 3f shows a representative Raman spectrum of the heterojunction, which shows the vibrational modes of each layered crystal. The Raman peaks for MoS_2_ in the flakes and at the interface did not differ significantly (e.g., the energy difference between *E*_2g_ and *A*_1g_ peaks ≈ 19 cm^−1^), suggesting the growth of monolayer MoS_2_. The HAADF-STEM image (Fig. 3g) and its indexed diffractogram (Fig. 3h) confirm epitaxial alignment between monolayer MoS_2_ and PtTe_2_. The STEM(-EDS) and corresponding (inverse) FFT study also verified the formation of a well-stitched lateral heterojunction comprising high-quality atomic layers (Supplementary Fig. 20). We did not find any significant differences from the bare MoS_2_ flake in the XPS characterization of MoS_2_ grown along the PtTe_2_ array (Supplementary Figs. 8c, d).

### Transport in the edge-contacted PtTe_2_-MoS_2_ MSJ FET arrays

**Fig. 4:**
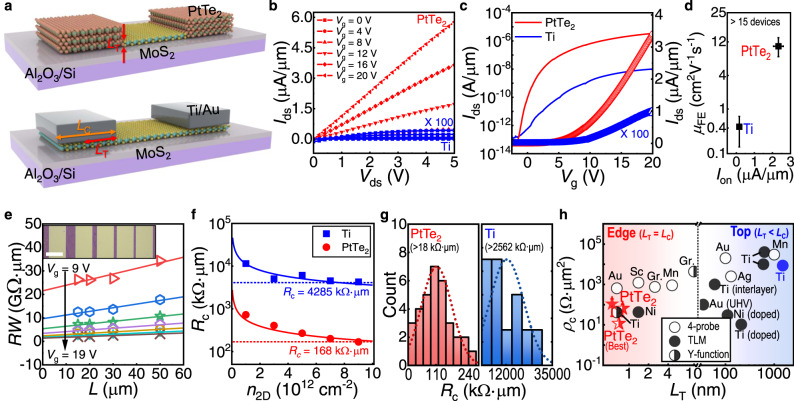
Electrical transport studies of the edge-contacted PtTe_2_-MoS_2_ MSJ FETs array. **a** Schematic showing monolayer MoS_2_ FETs with patterned PtTe_2_ edge contacts (upper) and vertical Ti contacts (lower). The transfer length (*L*_T_) of the carrier and physically attained contact length (*L*_c_) are schematically demonstrated. **b** Representative *I*_ds_–*V*_ds_ characteristics of monolayer MoS_2_ FET with symmetric PtTe_2_ and Ti contact electrodes depending on *V*_g_. **c** *I*_ds_–*V*_g_ characteristics of corresponding devices on linear (symbols; right) and logarithmic scale (lines; left). **d** Average *μ*_FE_ and *I*_on_ of different FETs (>15 devices) contacted to PtTe_2_ (red) or Ti (blue). The error bars indicate the standard deviations of each measurement set. **e–g** Determination of contact resistance (*R*_c_) of MSJs with different compositions using (**e**, **f**) TLM and (**g**) *Y*-function method. **e** TLM plot showing total resistance normalized by contact width (*RW*) as a function of *L*. The *y*-intercept *y*ields the 2*R*_c_ in units of Ω ∙ μm. Inset shows OM image of MoS_2_ (purple-like) grown between edges of PtTe_2_ patterned for TLM. (f) TLM-derived *R*_c_ of PtTe_2_-MoS_2_ MSJ FETs approaching ~168 ± 127 kΩ∙μm when the carrier density (*n*_2D_) of ~9 × 10^12^ cm^−2^ was largely induced by *V*_g_ (red circles), which is significantly lower than that of Ti/MoS_2_ (~4,285 ± 1,959 kΩ∙μm, blue squares). Solid lines specify curves fitted to the relation of *R*_c_ depending on the *n*_2D_ (*R*_c_ ∝ *n*_2D_^−0.5^)^2^. **g** Histograms of *R*_c_ for PtTe_2_-MoS_2_ (left) and Ti/MoS_2_ MSJ FETs (right) extracted by the *Y*-function method (Supplementary Fig. 22). The lowest *R*_c_ of PtTe_2_-MoS_2_ obtained in this study was ~18.2 kΩ·μm and the average value reached ~113.0 ± 60.3 kΩ·μm. **h** Benchmark specific contact resistivity (*ρ*_c_) and *L*_T_ of synthetic PtTe_2_ contacts. The experimental data from MoS_2_ FETs with lateral graphene contact (Gr.^16,55^), 3D edge contacts (Ti^11^, Ni^5^, Au^10^, Sc^54^, and Mn^13^), and top contacts (Ti^6,58^, Ni^59^, Au^7,10^, Ag^9^, and Mn^13^) are demonstrated. The colored symbols are the extracted data for PtTe_2_ (red stars) and Ti (blue circle) in this work. For fair comparison, the data are sorted by *ρ*_c_ extraction methods, i.e., four-point probe (open), TLM (solid), and *Y*-function (half-open symbol).

Arrays of FETs composed of edge-contacted MSJs patterns were fabricated and operated by using an Al_2_O_3_ back-gate dielectric (Fig. 4a). Analysis of the output and transfer characteristics (Fig. 4b, c) showed that the monolayer MoS_2_ FETs with PtTe_2_ symmetric contacts exhibited a maximum *μ*_FE_ value of ∼17.9 cm^2^ V^−1^ s^−1^ (∼10.6 ± 2.9 cm^2^ V^−1^ s^−1^ on average), *I*_on_ of up to ∼3.4 μA/μm (∼2.3 ± 0.3 μA/μm) and *I*_on_/*I*_off_ in the range of ∼10^7^ to 10^8^, which are much higher than those fabricated using vertical Ti contacts on monolayer MoS_2_ FETs with the same device geometries (channel *L* and *W*). We repeatedly observed performance enhancement for a large number of devices (>15), where the averaged *μ*_FE_ of the Ti/MoS_2_ FET was ∼0.4 ± 0.3 cm^2^ V^−1^ s^−1^ and the *I*_on_ was ∼0.2 ± 0.1 μA/μm (Fig. 4d). It is worthwhile to note that, considering their *L* and *L*_T_ values, the *μ*_FE_ value of the MoS_2_ FETs with PtTe_2_ edge contacts were either comparable or even higher than those of previously reported devices with various 3D edges (e.g., Sc^54^, Ti^50^, Au^10^, and Mn^13^) or 2D lateral contacts (e.g., graphene (Gr.)^17,18,55,56^, and VS_2_^14^) (Supplementary Figs. 21a–c).

To determine whether the *μ*_FE_ of a device was severely underestimated because of the contact property, we extracted the intrinsic mobility (*μ*_0_) of more than 30 different devices using the *Y*-function method (Supplementary Fig. 22). The *μ*_0_ value is typically free from underestimation due to *R*_c_ and provides a better indication of the intrinsic performance^11,55,57^. The averaged *μ*_0_ value in our PtTe_2_-MoS_2_ MSJ FETs was calculated to be ∼11.1 ± 4.5 cm^2^ V^−2^ s^−1^, which is almost comparable to the *μ*_FE_ values (∼10.6 ± 2.9 cm^2^ V^−2^ s^−1^) calculated using the transconductance (*g*_m_ = ∂*I*_ds_/ ∂*V*_g_). The insignificant difference between *μ*_FE_ and *μ*_0_ (∼4.5%) implies the presence of a minor contact barrier at the edge interface.

Notably, by using a MSJ fabricated with transfer length method (TLM) patterns, we could determine the *R*_c_ values of the monolayer MoS_2_ FETs with PtTe_2_ edge contact (Fig. 4e, f). The TLM-driven *R*_c_ reached ∼168 ± 127 kΩ·μm when a carrier density (*n*_2D_) of ∼9 × 10^12^ cm^−2^ was attained, which was largely induced by a *V*_g_ of ∼19 V. This *R*_c_ value is ∼25 times lower than that of its counterpart using Ti (∼4,285 ± 1,959 kΩ·μm) in Fig. 4f. We note that the demonstration of *R*_c_ as a function of *n*_2D_ (= *C*_ox_(*V*_g_-*V*_th_)/*q*) helps compare devices because *n*_2D_ includes information on the threshold voltage (*V*_th_) that can be varied by channel doping, the gate dielectric layer, and the interfacial trap density of the substrate^2,6,7^. The *R*_c_ was also calculated using the *Y*-function method^11,55,57^ (Fig. 4g and Supplementary Fig. 22), where the minimum *R*_c_ of the device was ∼18 kΩ·μm (∼113 ± 60 kΩ·μm, on average) and ∼2,562 kΩ·μm (∼12,443 ± 8,406 kΩ·μm) for PtTe_2_-MoS_2_ and Ti/MoS_2_, respectively. The average *R*_c_ values were comparable to those extracted from the TLM, demonstrating the reliability of the characterization methods. The low *R*_c_ obtained with PtTe_2_ allowed the FETs to outperform the Ti/MoS_2_ MSJ FETs (Fig. 4b–d).

The significantly low TLM-driven *R*_c_ at the monolayer-thick interface (e.g., *L*_T_ ≈ 0.7 nm) of the spatially-arranged PtTe_2_-MoS_2_ MSJ suggests that the contact length (*L*_c_) can be further scaled without an increase in *R*_c_ (Fig. 4h), as an advantage. For 3D top contacts, *R*_c_ increases as the *L*_c_ decreases below the *L*_T_ because current crowding becomes severe, according to the transmission line model^6,7^ expressed as *R*_c_ = $\sqrt{{\rho }_{c}{R}_{{sh}}}$cot(*L*_c_/*L*_T_), where *L*_T_ = $\sqrt{{\rho }_{c}/{\rho }_{{sh}}}$ and *ρ*_c_ (= *R*_c_ ∙ *L*_T_) is the specific contact resistivity (Supplementary Fig. 23). Thus, there have been tremendous efforts (i.e., insertion of an interlayer^6^, chemical doping^58,59^, and metal deposition under UHV conditions^7^) to achieve immunity of *R*_c_ to *L*_c_ scaling by lowering both *ρ*_c_ and *L*_T_ (right panel in Fig. 4h). However, the achievement of a small *L*_T_ (< tens of nanometers) together with low *ρ*_c_ is inherently challenging for 3D top contacts^8–10^. Instead, the edge contact can prospectively afford a negligible *L*_T_ because carrier transport occurs strictly through the interface of the edge^5,11,13^ as depicted in Fig. 4a.

We summarize the device performances of MoS_2_ FETs with 3D edge^5,10,11,13,50,54^ or 2D lateral contacts^14–18,55,56^ (Supplementary Table 5). Although a direct comparison of the contact properties with our device is inappropriate because of the differences in the *L*_T_ values, MoS_2_ channel thicknesses, and *R*_c_-extraction methods (see Supplementary Note 3 for more details), the developed PtTe_2_-MoS_2_ MSJ had a significantly low *ρ*_c_ (as low as ∼11.7 Ω·μm^2^) and *L*_T_ ≈ 0.7 nm, which is the lowest value among those reported for few-layer MoS_2_ FETs with 2D lateral graphene^16,55^, or 3D edge (Ti^11^, Ni^5^, Au^10^, Sc^54^, and Mn^13^) contacts (see the left panel in Fig. 4h and Supplementary Fig. 21d), thus promising the realization of ultralow *R*_c_ in *L*_c_-scaled transistors for next-generation 2D nanoelectronics. We propose that even further reduction of *R*_c_ and SBH is possible in our PtTe_2_ edge contact by achieving a multilayer lateral 2D-2D MSJ heterostructure, which can be attributed to the effectively screened interfacial traps from the substrates^13,60^, the downshift of the conduction band edge in multilayer MoS_2_^29^, and the weaker current crowding at the thicker heterointerface^6–10,13^. Moreover, the use of high-*k* dielectrics (e.g., HfO_2_, Sb_2_O_3_, SrTiO_3_), passivation layers (e.g., BN), and top-gate structures, which were already applied in the previous edge-contact FETs^10,11,13,18,55^, can also improve the performance of our device, which can be due to the suppressed charge scattering and trapped states from surrounding disorders^60^.

## Discussion

We report here a spatially-controlled, reproducible preparation of metallic vdW PtTe_2_ crystals as a lateral edge contact with semiconducting monolayer MoS_2_. The high-quality stoichiometric PtTe_2_ retained its surface properties even after CVD at the high temperature of ∼750 °C under UHV conditions. The edge of thermally stable PtTe_2_ provides nucleation sites for the subsequently grown 2D semiconductor without noticeable thermal degradation, resulting in in-plane lateral MSJs without substantial interfacial issues such as alloying or void-like defects. Therefore, the PtTe_2_-MoS_2_ MSJ may have a simple resistance network and displays superior *n*-type carrier transport through the short and narrow thermionic barriers, enabling the higher performance of the FET compared to that composed of vertical 3D contacts. This approach also provides a more scalable way to produce an arrangement of lateral heterostructures in a dimension-controlled manner, where the contact properties could be evaluated by using the TLM patterns consisting of the lateral MSJ. The substantially small contact resistivities achieved through the atomically thin edge suggest that the developed contact scheme has the potential for scaling the contact length for miniaturized 2D electronics.

Our work on the synthesized edge-contact MSJ arrays offers benefits in terms of scalability for both material synthesis and device fabrication. The edge contact between 2D (or 3D) metals and 2D semiconductors should be developed and evaluated based on CVD-grown 2D layers to investigate its practical potential for semiconductor technology in the future. However, almost all investigations on lateral MoS_2_-based MSJs have drawbacks in terms of reproducibility or the achievement of pure edge contact (Supplementary Table 4). In this regard, our strategy based on direct growth of a large-area 2D metal on a dielectric substrate followed by MoS_2_ epitaxy has considerable advantages over other methods using 2D metals such as Mo_2_C^15^ and graphene^16–18,55,56^, formed by mechanical exfoliation of single crystal and/or transfer of CVD-grown layers. More importantly, the realization of pure edge contacts with the *L*_T_ reduced to ∼1 nm using graphene^16–18,55,56^ or VS_2_^14^ is highly challenging because of the inevitable laterally overlapped junction or alloyed structures.

In addition, the construction of TLM patterns using a TMD-based synthetic edge contact provides assurance by permitting a better systematic analysis. Because of the complexity of realizing a lateral MSJ, it is difficult to achieve reproducible data from multiple devices and to perform statistical computations for *R*_c_^10,11,13–16,18,54,55^. Investigating *R*_c_ of edge-contact MSJs using TLM pattern proves particularly challenging because of channel-to-channel variations and deficiencies in integration scalability. Many of the studies on lateral MSJ rely on the four-point measurement^10,13–16,18,54^ or *Y*-function methods^11,55^ for *R*_c_ extraction, which can be inaccurate compared with the TLM (Supplementary Note 3). Therefore, together with the reproducibility of our 2D material system, the statistical analysis of the *R*_c_ values extracted by TLM and the Y-function method in this study can provide better insights into the electrical features of 2D-2D edge contacts.

## Methods

### Growth of PtTe_2_ flakes

The growth of multilayer PtTe_2_ single crystals was conducted using a conventional horizontal furnace system, in which the Te-Pt precursors on a SiO_2_/Si substrate were placed inside the center of the chamber. To prepare the precursor sample, a Pt thin film precursor (∼2 nm, 99.9% purity pellet) was deposited using an e-beam evaporator (Temescal FC-2000), and then 0.1 g of Te powder was introduced manually to cover up the thin film. The reactant was then heated to ∼700 °C (at a heating rate of ∼50 °C/min) under a pressure of ∼0.1 Torr using H_2_ (10 sccm) as the carrier gas. After maintaining a growth temperature of ∼700 °C for 1 min, the furnace was naturally cooled to room temperature. We succeeded in synthesizing PtTe_2_ directly on top of the SiO_2_/Si substrate because the unreacted Te was vaporized and sucked out toward the vacuum pump.

### Preparation of patterned PtTe_2_ thin films

The pre-deposited, poly-crystalline Pt thin film (∼1 nm) on the SiO_2_/Si substrate and Te powder (∼0.1 g) inside a quartz boat were placed in a horizontal furnace ∼1 cm apart. Under low pressure (0.1 Torr) at ∼400 °C, the evaporated Te vapor reacts with the Pt precursor, resulting in a homogenous, uniform PtTe_2_ layer with a thickness of ∼4 nm. The thickness of the as-grown thin film could be controlled by modulating the Pt precursor’s thickness. For position-controllability, the shape of any desired pattern was defined using photolithography (MIDAS MDA-400S) with a photoresist with an undercut profile (DPRi-1549), followed by Pt deposition and lift-off. Using the same method for thin films, the shaped Pt precursor could be tellurized, resulting in successful growth of the PtTe_2_ patterns.

### Synthesis of MoS_2_ along the edge of PtTe_2_

The MoS_2_ flakes were synthesized using MoO_3_ thin film (∼1 nm-thick on a 1 cm^2^ SiO_2_/Si substrate, evaporated using an e-beam evaporator) and S powder as precursors in atmospheric-pressure two-zone CVD with Ar/H_2_ as the carrier gas. To promote the nucleation of MoS_2_, ∼1 μL of a 0.01 M NaCl promoter solution was pipetted on the corner of the oxide thin film on the substrate. Then, the NaCl solution was baked at ∼100 °C to evaporate the water entirely. The formerly prepared PtTe_2_ multilayers on the substrate were placed face to face on top of the MoO_3_ film prepared with NaCl. This metal oxide/PtTe_2_ sample was placed at the center of the heat zone of the furnace, with the S-powder-containing boat loaded upstream of the CVD furnace. Under an Ar/H_2_ ratio of 70/20 sccm, the system was steadily heated to ∼700 °C. At a growth temperature of ∼700 °C, S vapor was introduced because the powder was heated at ∼200 °C just before reaching the growth temperature and then maintained constantly for a growth time of ∼10 min. The as-synthesized PtTe_2_ sample was stored in a chamber under UHV atmosphere (∼10^10^ Torr) immediately after synthesis, and the air exposure time of PtTe_2_ until the subsequent CVD process was <5 min.

### Structural characterization

The morphological investigation was conducted using an SEM (Hitachi S-4800 or Su8220) equipped with high- and low-angle BSE detectors. Micro-Raman measurements were performed with a 532 nm laser (Thermo Scientific DXR2 Raman Microscope) configured for wavenumber precision of ≤0.066 cm^−1^. XRD patterns were captured using a Bruker AXS D8 instrument with a Cu K*α* source. AFM images were recorded on a Bruker Dimension AFM operating in tapping mode. High-resolution STEM images, SAED patterns, EDS were obtained using an aberration-corrected FEI Titan^3^ G2 60-300 equipped at an acceleration voltage of 200 kV. Noises of high-resolution STEM images were subtracted by Wiener filter. EELS was performed using a Gatan Quantum 965 dual EELS system with an energy resolution of 1.0 eV under an acceleration voltage of 200 kV. Specimen for cross-section TEM analysis were prepared by focused ion beam (FEI Helios Nanolab 450HP). XPS and UPS measurements were performed using an ESCALAB 250XI system (ThermoFisher K-alpha) equipped with an Al X-ray source under UHV conditions. The calibration of the XPS was performed by the alignment of the C 1 *s* spectrum (whose binding energy is 284.5 eV).

### DFT calculation

We constructed a 4 × 4 × 1 PtTe_2_ supercell with vacuum along the *b* and *c* directions to model a PtTe_2_ single layer strap with an edge having 50% Te coverage. We performed geometry optimization based on DFT calculations using the supercell and the Vienna ab initio software packet^61^ (VASP) code. We employed the projector-augmented-wave (PAW) method^62^ and the Perdew-Burke-Enzerhof (PBE) functional^63^ using a plane-wave basis set with an energy cutoff of 500 eV. The k-points were sampled using a 4 × 4 × 1 Monkhorst-Pack mesh^64^, and the spin-orbit coupling effect was also included.

### Electrical device fabrication and measurements

To define the device channel, the shape of the channel layer was defined by e-beam lithography (NBL and NB3), and then a reactive ion etching process was conducted using SF_6_ and O_2_ plasma. The etched structures were transferred to a dielectric layer on highly *p*-doped Si, which can be used as the back-gate. For instance, the single-crystalline PtTe_2_-MoS_2_ MSJ was transferred to a 300-nm-thick SiO_2_ layer, whereas the conformally grown array was transferred to a 50-nm-thick Al_2_O_3_ layer. The well-connected heterointerface between PtTe_2_ and MoS_2_ confirmed through TEM analysis (Figs. 1j and 3g), proves that the wet transfer method utilizing a polymeric supporting layer did not affect the edge contact of the samples. The SiO_2_ layer was dry-oxidized in a furnace (KHD-306) with ±3% uniformity, and the Al_2_O_3_ dielectric layer was prepared by atomic layer deposition (Lucida, D100), being deposited within ±2% uniformity along the wafer. Ti/Au (∼10 and ∼70 nm, respectively) contacts and pads were then deposited using e-beam lithography and an e-beam evaporator. Electrical characterizations at different temperatures (138-300 K) were performed using a Keithley 4200-SCS detector in a cryogenic probe station (Lakeshore CRX-4K) under a high vacuum (∼10^−6^). The *μ*_FE_ of the FETs on the Al_2_O_3_ dielectric insulator were determined by measuring the gate oxide capacitance per unit area (*C*_ox_) of Al_2_O_3_ via *C*-*V* analysis of the metal-insulator-semiconductor structure (e.g., Pt/Al_2_O_3_/*p*^++^Si), where the DC voltage was swept from −5 to 10 V, while an AC voltage with an amplitude of ∼100 mV and frequency of ∼20 kHz was applied. The calculated *C*_ox_ in the accumulation region was 0.164 ± 0.001 μF cm^−2^ (average of ten devices), which corresponds to an equivalent oxide thickness of ∼117.9 nm.

## Supplementary information

Supplementary Information

## Data Availability

Relevant data supporting the key findings of this study are available within the article and the Supplementary Information file. All raw data generated during the current study are available from the corresponding authors upon request.
