# Supplementary material for: Atomic transistors based on seamless lateral metal-semiconductor junctions with a sub-1-nm transfer length
Source: Nat Commun. 2022 Aug 22;13:4916. doi: 10.1038/s41467-022-32582-9 (PMC9395343; doi:10.1038/s41467-022-32582-9)
Supplement: Supplementary file 1 — Supplementary Information [file 41467_2022_32582_MOESM1_ESM.pdf]

Supplementary information:

Atomic transistors based on seamless lateral metal-semiconductor junctions with a sub-1-nm transfer length

Seunguk Song[1,2], Aram Yoon[1,3], Jong-Kwon Ha[4], Jihoon Yang[1], Sora Jang[1], Chloe Leblanc[2], Jaewon Wang[1], Yeoseon Sim[1], Deep Jariwala[2], Seung Kyu Min[3,4], Zonghoon Lee[1,3*], and Soon-Yong Kwon[1*]

[1]*Department of Materials Science and Engineering & Center for Future Semiconductor Technology (FUST), Ulsan National Institute of Science and Technology (UNIST), Ulsan 44919, Republic of Korea*
[2]*Department of Electrical and Systems Engineering, University of Pennsylvania, Philadelphia, PA 19104, USA*
[3]*Center for Multidimensional Carbon Materials (CMCM), Institute for Basic Science (IBS), Ulsan 44919, Republic of Korea*
[4]*Department of Chemistry, Ulsan National Institute of Science and Technology (UNIST), Ulsan 44919, Republic of Korea*

[*]Correspondence should be addressed. Email to: zhlee@unist.ac.kr (Z.L.), sykwon@unist.ac.kr (S.-Y.K.)

- Supplementary Figures 1-23
- Supplementary Tables 1-5
- Supplementary Notes 1-3
- Supplementary References 1-78

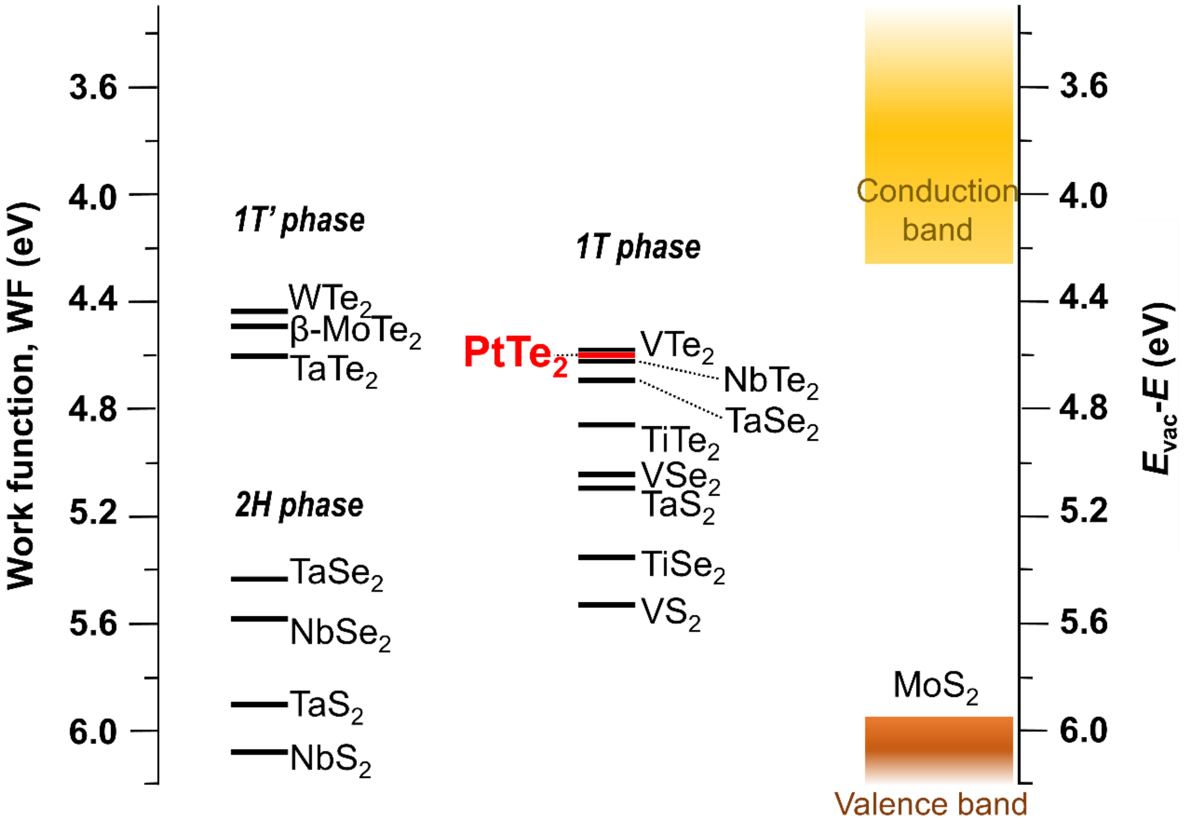

Supplementary Fig. 1. Work function (WF) of $PtTe_2$ and other metallic vdW TMDs. Left: WF of different metallic TMDs with different phases (1T', 2H, and 1T); right: electron affinity and ionization potential of MoS_2. Only the metallic TMDs phases achievable by the chemical vapor-based synthesis are demonstrated. Other than that of $PtTe_2$, the demonstrated values were calculated by density functional theory[1-4]. According to the Schottky-Mott rule, a small SBH can be achieved by using low-WF metals for *n*-type semiconductors (e.g., SBH = WF – χ, where χ is the electron affinity of monolayer MoS_2 (≈ 4.28 eV)[5]).

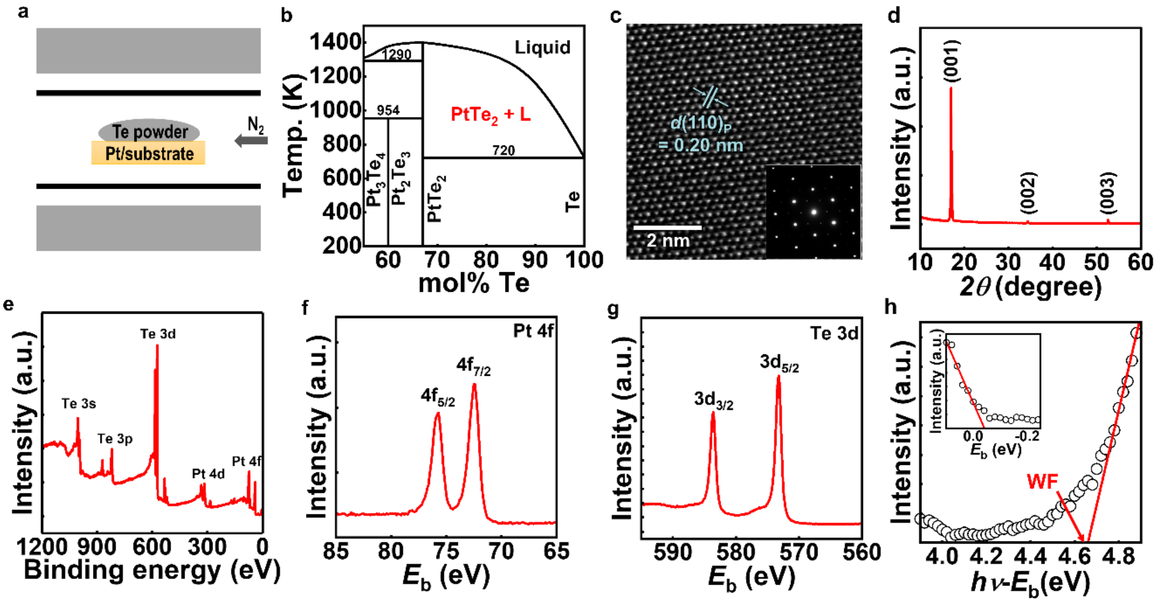

Supplementary Fig. 2. Synthesis of high-quality $PtTe_2$ single crystals by eutectic solidification. (a) Schematic of the growth process for the $PtTe_2$ flakes on the desired substrate at the growth temperature of ~700 °C. (b) Phase diagram of the binary Pt-Te system, used in the growth scheme. (c) HAADF-STEM image of a $PtTe_2$ crystal showing the atomic distance in the (110) plane ($d(110)_P \approx 0.20$ nm). The inset shows the representative diffraction patterns of the crystal with hexagonal spots of (100) and (110) planes, indicating its single-crystalline nature. (d) XRD pattern of the as-grown sample showing peaks for (00$1l$) planes of $PtTe_2$. (e-g) XPS characterization of a $PtTe_2$ crystal, (e) Full-range XPS spectrum, (f) Pt 4*f*, and (g) Te 3*d* scans. The oxide-related peaks were absent from the spectra (at.% Te:Pt = 1.97:1). (h) UPS spectrum of multilayer $PtTe_2$ crystal in the high-energy regime, with the WF indicated by the *x*-intercept. The inset shows the UPS spectrum with regards to the valence band offset.

: Because the liquid-like eutectic phase has only $PtTe_2$ crystal (liquid ⇔ Te (s) + $PtTe_2$ (s)) at a growth temperature of ~700 °C (Supplementary Fig. 2b), we were not concerned about the formation of other phases such as Pt_2Te_3 or Pt_3Te_4. The unreacted Te evaporates during the growth because of the high Te vapor pressure, resulting in high-quality $PtTe_2$ directly on top of the substrate. Supplementary Fig. 2c shows the HAADF-STEM image of $PtTe_2$ displaying the periodic atomic arrangement of $PtTe_2$ with a (110) lattice plane spacing of ~0.20 nm, which is in agreement with the atomic structure of the mechanically exfoliated flakes[63]. The corresponding SAED pattern (inset of Supplementary Fig. 2c) shows the specific set of diffraction spots, indicating the single-crystalline nature of the as-grown $PtTe_2$. Representative signals for pure $PtTe_2$ were further verified by XRD characterization (Supplementary Fig. 2d) as corresponding to the three (00$1l$) planes, which signify that the structures are highly aligned with the *c*-plane-orientation. Furthermore, XRD peaks related to by-products or indicating an

incomplete reaction (e.g., Pt or Te) were absent. XPS characterization showed strong signals corresponding to Pt-Te binding energies from Te 3d and Pt 4f scans (Supplementary Figs. 2e-g). Owing to its high crystallinity, the $PtTe_2$ surface showed filled energy levels as a metal without any forbidden gap, indicated by the sudden increase in intensity at the binding energy (E_b) of 0 eV in the UPS spectrum (inset of Supplementary Fig. 2h). On the other hand, the secondary electron cutoff in the UPS spectrum (Supplementary Fig. 2h) shows an x-intercept of ~4.65 eV, which corresponds to the WF of the $PtTe_2$.

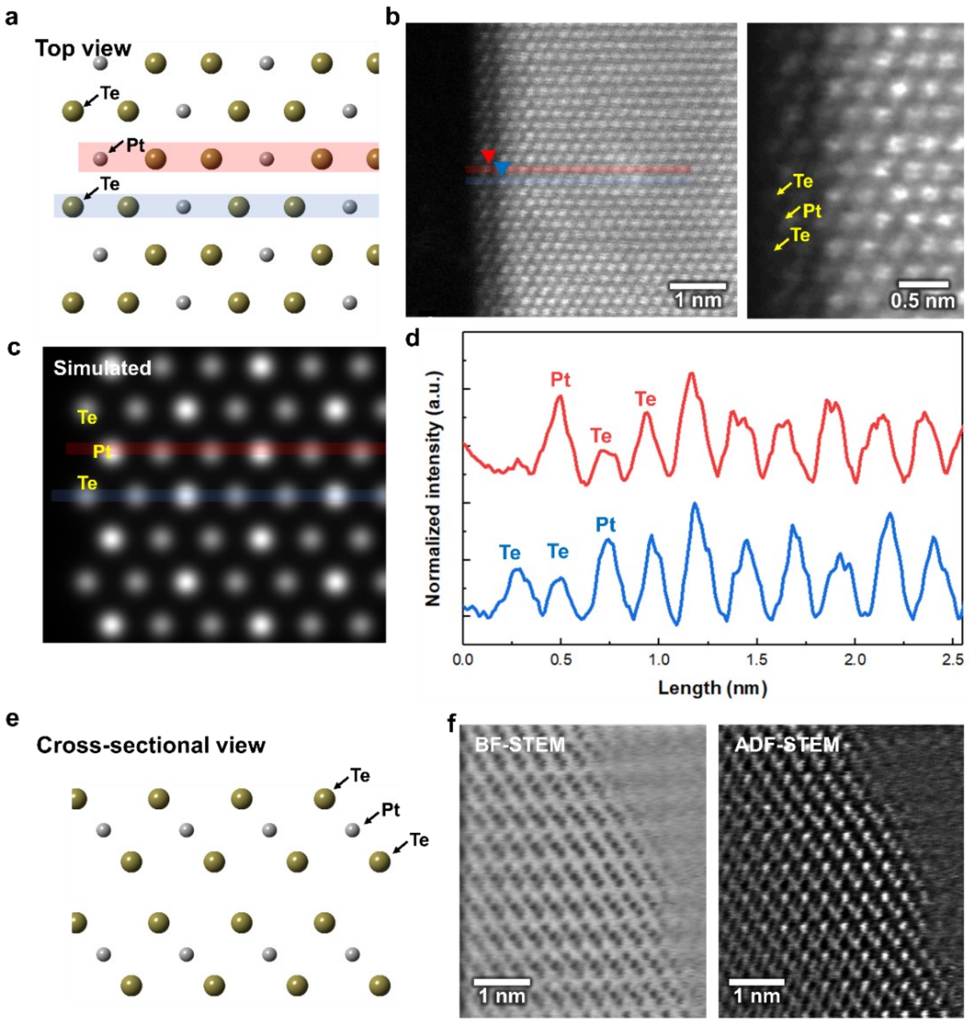

Supplementary Fig. 3. Atomic structures of $PtTe_2$ bare flakes and as-synthesized $PtTe_2$ crystals analyzed by TEM. (a–d) Top-view structural analysis of $PtTe_2$ flake. (a) Atomic-scale schematic of $PtTe_2$ showing its 1T phase feature and Te-termination of the structure at the left edge. (b) (left) atomic-resolution HAADF-STEM image of the few-layer $PtTe_2$ flake showing its high crystal quality, and (right) zoomed in HAADF-STEM image of the $PtTe_2$ edge showing the 50 % Te-termination. (c) Simulated HAADF-STEM image of $PtTe_2$ showing different intensities depending on the locations of the Pt and Te atoms. (d) Intensities profiles through the atomic columns along the red and blue lines in panel (b). (e–f) Cross-sectional TEM analysis of a $PtTe_2$ crystal. (e) Schematic cross-sectional view of $PtTe_2$ bilayer. (f) (left) BF- and (right) ADF-STEM of the cross-section of $PtTe_2$ showing the minor structural damage at the edge. The TEM sampling for the plain view was conducted under ambient air for ~3 h; thus, all the Te-terminated edges correspond to those of a clean, high-quality crystal without significant oxidation. The positive differential Gibbs free energies of adsorption of both H_2O (46.3 KJ mol^{-1}) and O_2 (1.8 KJ mol^{-1}) on $PtTe_2$ surfaces at 300 K reported in a previous study[7] may have prevented the adsorption of oxygen-related molecules on our $PtTe_2$ sample under ambient.

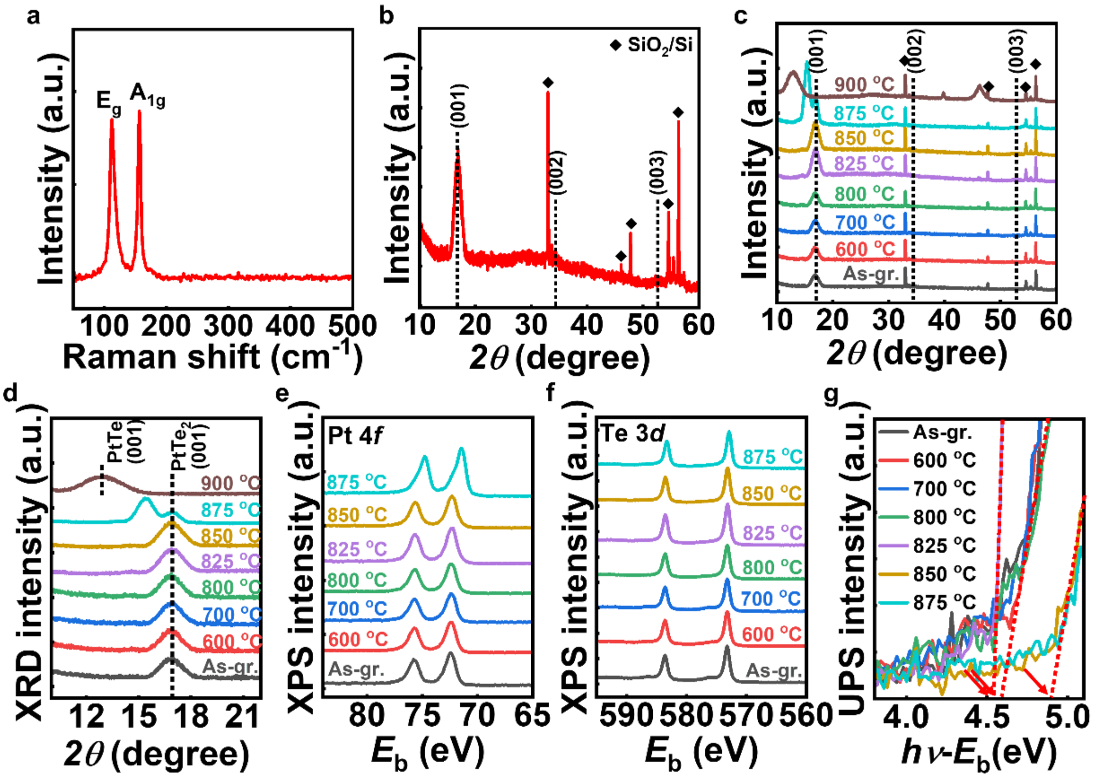

Supplementary Fig. 4. The thermal stability test of $PtTe_2$ crystals (*H* = 4 nm). (a) Representative Raman spectrum of the as-grown $PtTe_2$ thin film's surface. (b) XRD pattern of the corresponding $PtTe_2$, showing the distinct peak corresponding to the (001) plane at $2\theta \approx 16.77^{\circ}$ whereas other (001*l*) planes were not detected most likely because the film was only ~4 nm thick. (c-g) Thermal stability test at different *T* under UHV conditions. (c) Full-range XRD patterns of the $PtTe_2$ thin film samples annealed at different *T* under UHV (not normalized). The peak positions for (001*l*) plane of $PtTe_2$ are indicated as dashed lines. The diamond-marked peaks originated from the SiO_2/Si substrate. (d) Magnified XRD spectra for the thin film annealed at different *T*. The intensities were normalized to the highest peak. (e,f) XPS spectra of the $PtTe_2$ thin film for (e) Pt 4*f* and (f) the Te 3*d* scan. Only the sample heated at ~875 °C showed noticeable peak shifts with respect to that of the as-grown thin film. (g) UPS spectra in the high-energy regime for the $PtTe_2$ surface annealed at different *T*, where the WF was changed as the variation of the *x*-intercept (red arrows).

: To confirm the intrinsic thermal stability of $PtTe_2$ surface, we produce the $PtTe_2$ with a ultrathin layer with a 4 nm thickness. The power-based growth method was used instead of eutectic solidification; tellurization of a Pt precursor thin film at ~400 °C under low pressure (< 10^{-1} Torr) under H_2 flow (5 sccm). The thin film was found to be polycrystalline with an average grain size of ~30 nm. Heat treatment up to 850 °C did not induce a change in the XPS spectrum of the Pt-4f core levels, which had binding energies of 75.8 ($4f_{5/2}$) and 72.4 ($4f_{7/2}$) eV

with respect to the as-grown sample, whereas the spectrum of the crystal annealed at 875 ºC showed peaks shifted to lower energies (Supplementary Figs. 4d, e). Similarly, the XPS-extracted stoichiometries were almost perfect up to $T \approx 825$ ºC; however, the samples annealed at the higher T (> 850 ºC) showed Te-deficiency (e.g., at. % (Te/Pt) ≈ 1.18 at $T = 875$ ºC.

The loss of Te at T above ~850 °C was also reflected in the XRD patterns (Supplementary Fig. 4c, d). The peak corresponding to the (001) plane was shifted from 2θ = ~16.71 to ~12.99°, due to the formation of PtTe with a wider interplanar spacing of ~0.67 nm[65] (compared to ~0.52 nm for $PtTe_2$[66]). Note that the Raman spectrum of the single crystal annealed at 1,000 ºC (yellow in Fig. 1c) also indicates the formation of PtTe, with new peaks at ~92, ~120, ~169, and ~186 cm^{-1} corresponding to the characteristic modes of PtTe (ref. [10]). The WFs characterized by ultraviolet photoelectron spectroscopy (UPS) ranged from 4.60 to 4.65 eV (Supplementary Fig. 4g) for the samples with T below 825 °C; however, annealing at a higher T (> 850 °C) shifted the WF to ~4.90 eV, which resembled that of Pt (ref. [11]) (~4.90 eV).

We assumed that the high quality of our $PtTe_2$ might provide thermal stability as described in the main text. The near-zero in-plane thermal expansion of $PtTe_2$ (~2.0×10^{-6} K^{-1}; smaller than those of graphene (~ -12.7×10^{-6} K^{-1}), MoS_2 (~4.9×10^{-6} K^{-1}), and WSe_2 (~6.8×10^{-6} K^{-1}))[43] may also contribute to the preservation of its lattice at high temperatures.

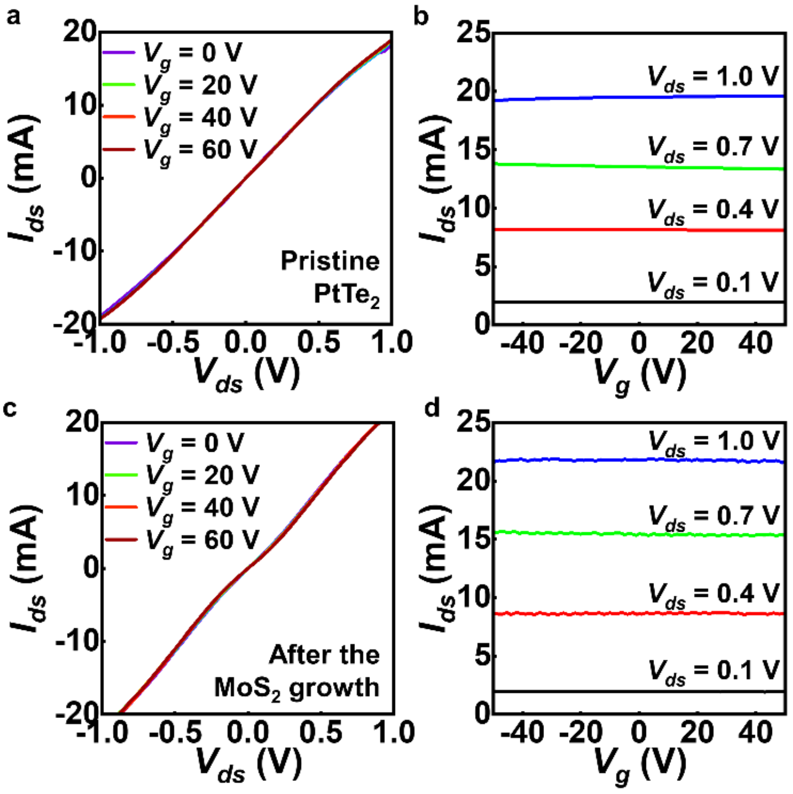

Supplementary Fig. 5. Two-terminal electrical characterization of $PtTe_2$ crystals. (a-b) The electrical characterization of the pristine $PtTe_2$ crystals, (a) output and (b) transfer curves for an as-prepared $PtTe_2$ crystal. The two-terminal resistivity ($\rho \approx 0.37$ mΩ·cm) was calculated by linear fitting of the output curve. (c-d) The electrical transport through the $PtTe_2$ region after the two-step growth of the lateral heterostructure fabricated with MoS_2. (c) Output curve showing the similarly low resistance properties ($\rho \approx 0.35$ mΩ·cm), sustaining within ~10% change. The change in ρ can be attributed to measurement error or to increased resistance with the contact pad caused by annealing. (d) Transfer characteristics of $PtTe_2$ after high-temperature CVD, showing the insignificant gate modulation of the conductivity.

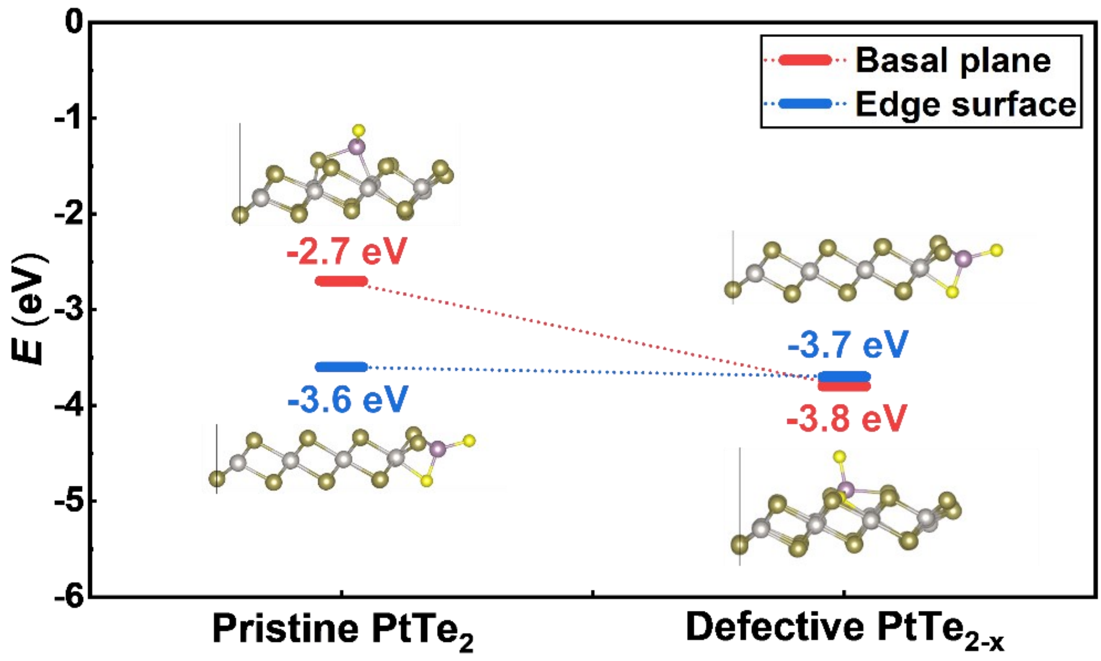

Supplementary Fig. 6. Adsorption energy comparison of MoS_2 monomer on basal plane and edge surface of pristine and defective $PtTe_2$. The adsorption energy represents the binding energy when a MoS_2 monomer is forcibly attached, but has no effect on the further development of MoS_2 growth.

: DFT simulations were performed to investigate the selective nucleation of MoS_2 on the edges of $PtTe_2$. Adsorption energy calculations reveal that there was preferential adsorption and subsequent nucleation of MoS_2 at the $PtTe_2$ edge. For instance, the MoS_2 monomer exhibited a lower adsorption energy of -3.6 eV at the $PtTe_2$ edge compared to -2.7 eV on the $PtTe_2$ basal plane. This substantial difference in the adsorption energy may explain the edge-mediated growth of MoS_2. Conversely, the energy calculation for the defective $PtTe_{2-x}$ with Te monovacancy had a different tendency than our actual case. The high thermal stability of $PtTe_2$ and its highly stoichiometric nature (Te/Pt atomic ratio ≈2) after the thermal CVD of MoS_2 were validated in Figs. 1c and 3d, and Supplementary Fig. 4. The calculation results for defective, nonstoichiometric $PtTe_{2-x}$ indicate that preferential growth of vertically overlapped MoS_2 on $PtTe_{2-x}$ can occur. This result is consistent with the growth of the overlapped MoS_2 layer on the basal plane of the defective $PtTe_{2-x}$ at a high T = 800 ºC in our experiment (at which T is higher than in our typical case of ~670-700 ºC).

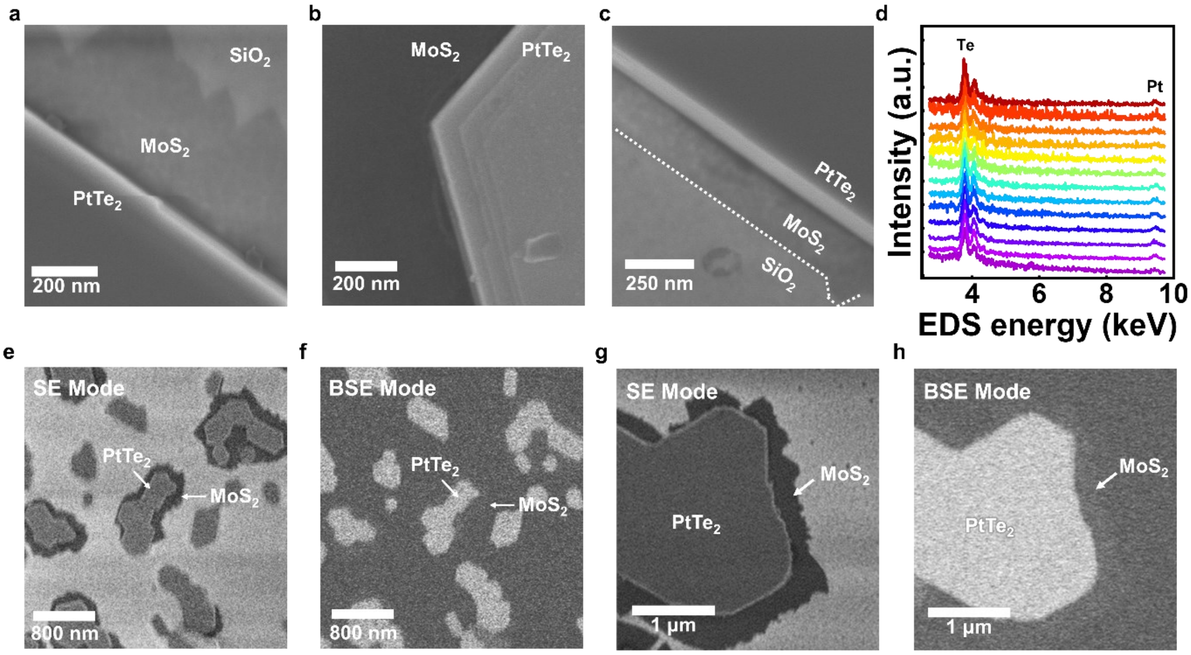

Supplementary Fig. 7. SEM analysis of the $PtTe_2$-MoS_2 lateral heterointerface. (a-c) SEM images of the MoS_2 layers synthesized along the edge of single-crystalline $PtTe_2$. No significant optical contrast was observed in $PtTe_2$, indicating the high thermal stability of the surface. (d) SEM-EDS spectra for $PtTe_2$ crystals after the edge growth of MoS_2, showing the strong Te and Pt signals even after high-temperature CVD. The 15 different spectra depicted were captured randomly at different heterostructures. (e-h) SEM analysis of the $PtTe_2$-MoS_2 heterointerface in BSE mode (> 100 nm) to observe the distribution of $PtTe_2$ and MoS_2. The SEM images in the normal SE mode (e, g) and corresponding images in the BSE mode (f, h) are depicted. Since Pt and Te have higher atomic masses than Mo and S, $PtTe_2$ appears much brighter than MoS_2. Along the basal surface of $PtTe_2$, no contrast difference in BSE-SEM images was observed.

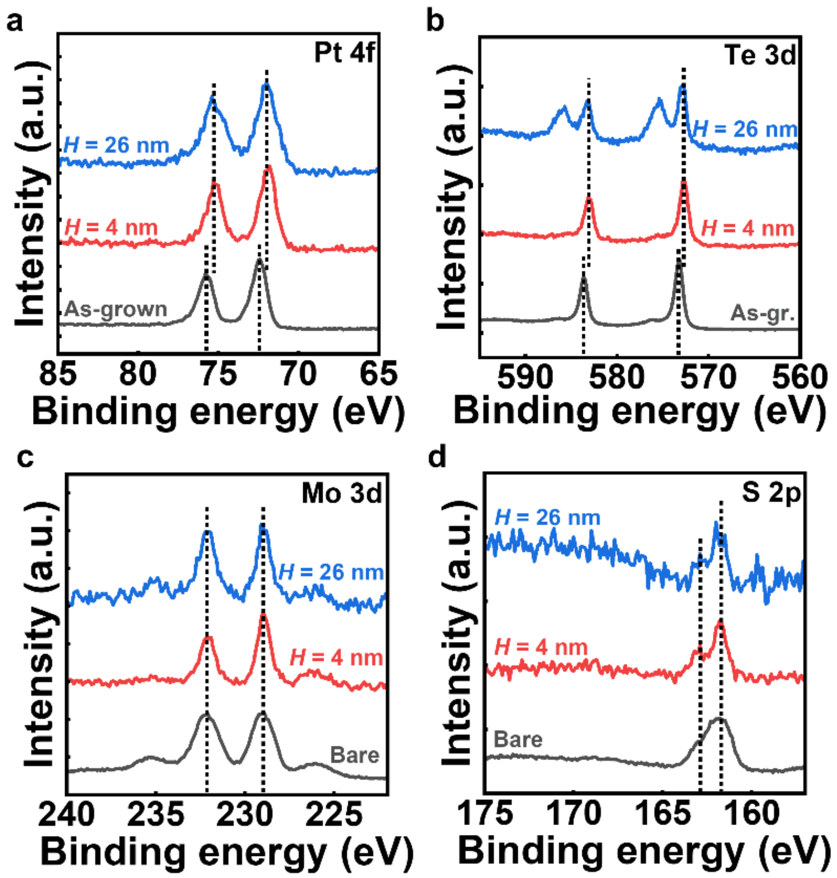

Supplementary Fig. 8. XPS analysis of in-plane heterostructure with poly- (*H* = 4 nm, red) and single-crystalline (*H* = 26 nm, blue) $PtTe_2$. The XPS spectra for Pt 4*f*, Te 3*d*, Mo 3*d*, and S 2*p* energy levels are demonstrated in (a)-(d), respectively. The spectra for as-grown $PtTe_2$ single crystal (grey in (a) and (b)), and bare MoS_2 grown independently on the substrate (grey in (c) and (d)) are displayed for comparison.

: XPS analysis revealed that there were no significant changes in the stoichiometry of $PtTe_2$ (~1.80), although the binding energies of Pt and Te shifted to lower levels (to ~600 meV) (Supplementary Figs. 8a, b). Recalling the negligible XPS peak shift of the Te-deficient sample annealed at T = 850 °C in Supplementary Fig. 4, the observed energy shift in Supplementary Figs. 8a, b may be related to the increased carrier density in the vicinity of $PtTe_2$ by the stitching of MoS_2 layer rather than the change in stoichiometry[69]. In contrast, the XPS spectra for the MoS_2 layer do not show a substantial peak shift compared to those of the non-stitched bare flakes. This suggests that our growth mode led to the MoS_2 layer acquiring the intrinsic surface properties of a 2D semiconductor because the MoS_2 layer was grown after the preparation of $PtTe_2$.

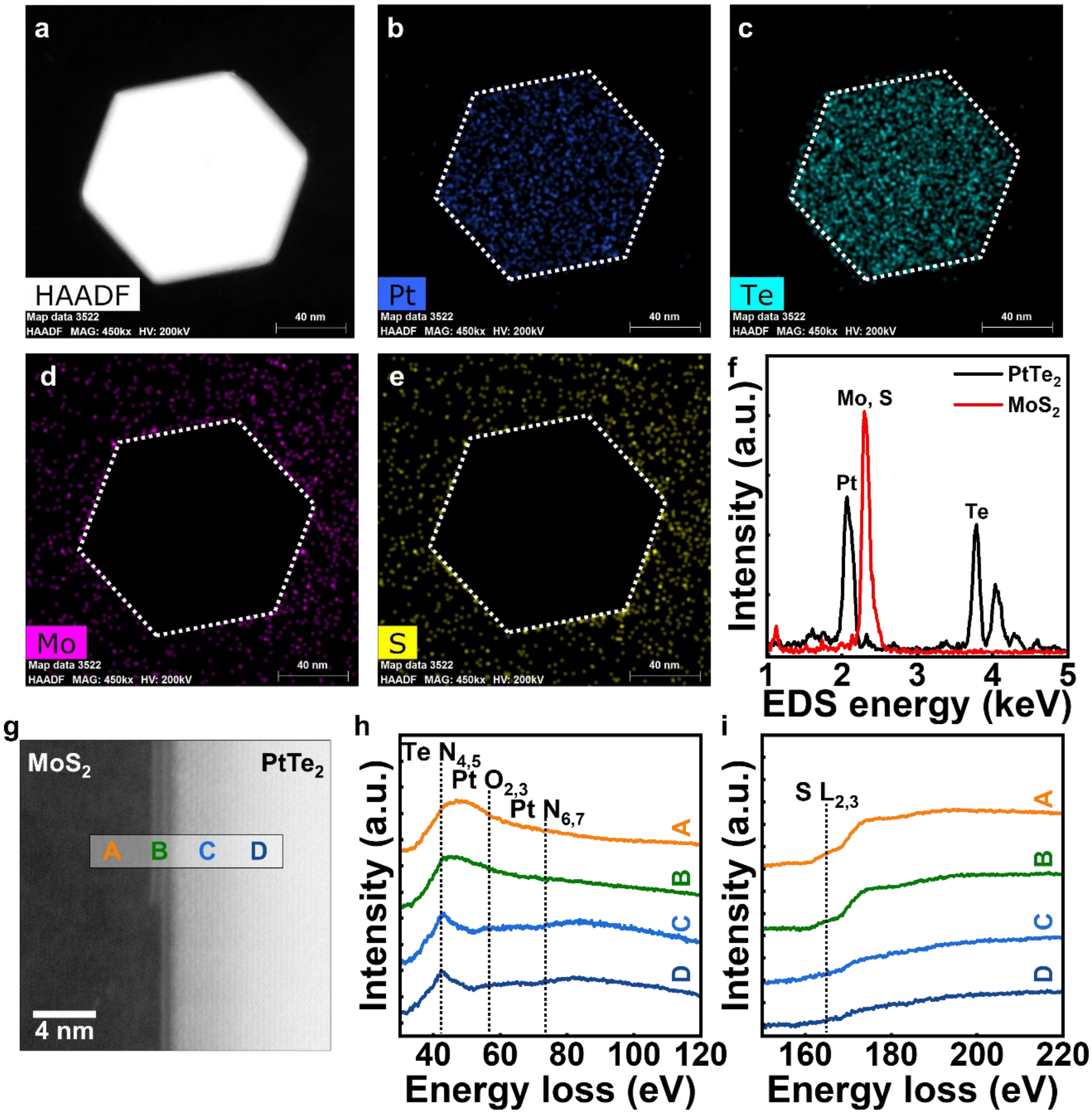

Supplementary Fig. 9. Compositional analysis of 2D-2D $PtTe_2$-MoS_2 lateral heterointerface conducted by (a-e) STEM-EDS and (f-h) STEM-EELS. (a-e) EDS elemental mapping images of the (b) Pt, (c) Te, (d) Mo, and (e) S for the corresponding $PtTe_2$-MoS_2 heterostructure in (a). The abrupt signal changes in Pt and Te suggest that the elements were not intermixed. In the case of Mo and S, the relatively high thickness of $PtTe_2$ leads to increased background noise, which can result in measurement errors in the $PtTe_2$ region, thereby the noise signals are removed here. (f) Representative STEM-EDS profiles of MoS_2 and $PtTe_2$ crystals of the lateral heterostructure. (g-i) EELS measurement for the heterointerface for scans of (h) Te-*N*, Pt-*O* and (i) S-*L*, conducted at the four locations of A-D in the vicinity of the $PtTe_2$-MoS_2 interface as shown in the HAADF-STEM image in (g).

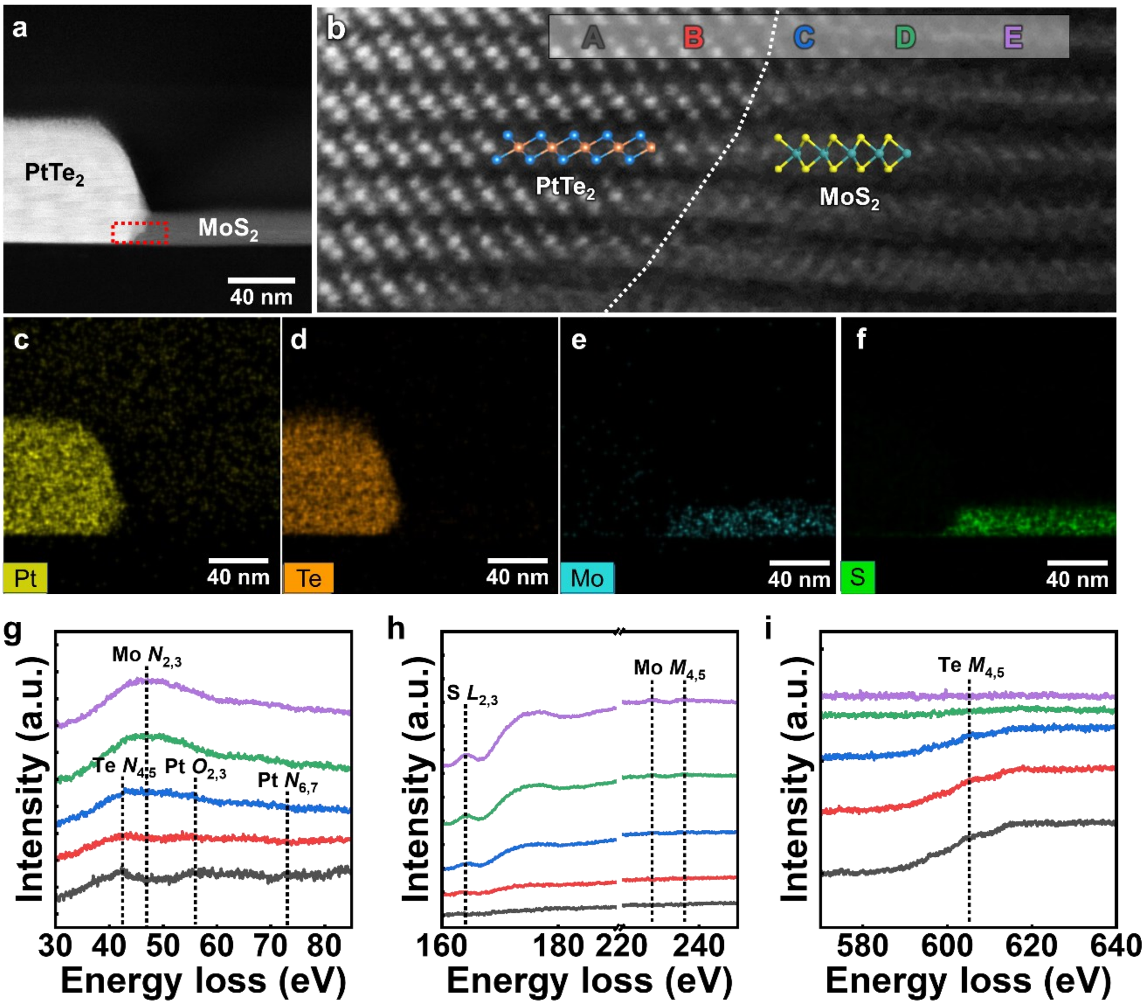

Supplementary Fig. 10. Cross-sectional TEM analysis of MoS_2-$PtTe_2$ lateral heterostructure. (a) Low-magnified STEM image of the heterostructure. (b) Atomic-resolution HAADF-STEM image captured at the marked red box in (a). (c-f) EDS mapping of the corresponding to (c) Pt, (d) Te, (e) Mo, and (f) S elements. (g-i) EELS plots for the heterointerface, conducted at the five locations of A-E in (b). For this measurement, we intentionally synthesized and characterized multilayer-MoS_2-connected $PtTe_2$ heterostructure to avoid failures in cross-sectional sample preparation for monolayer MoS_2 attributed to the damage by focused ion beam process.

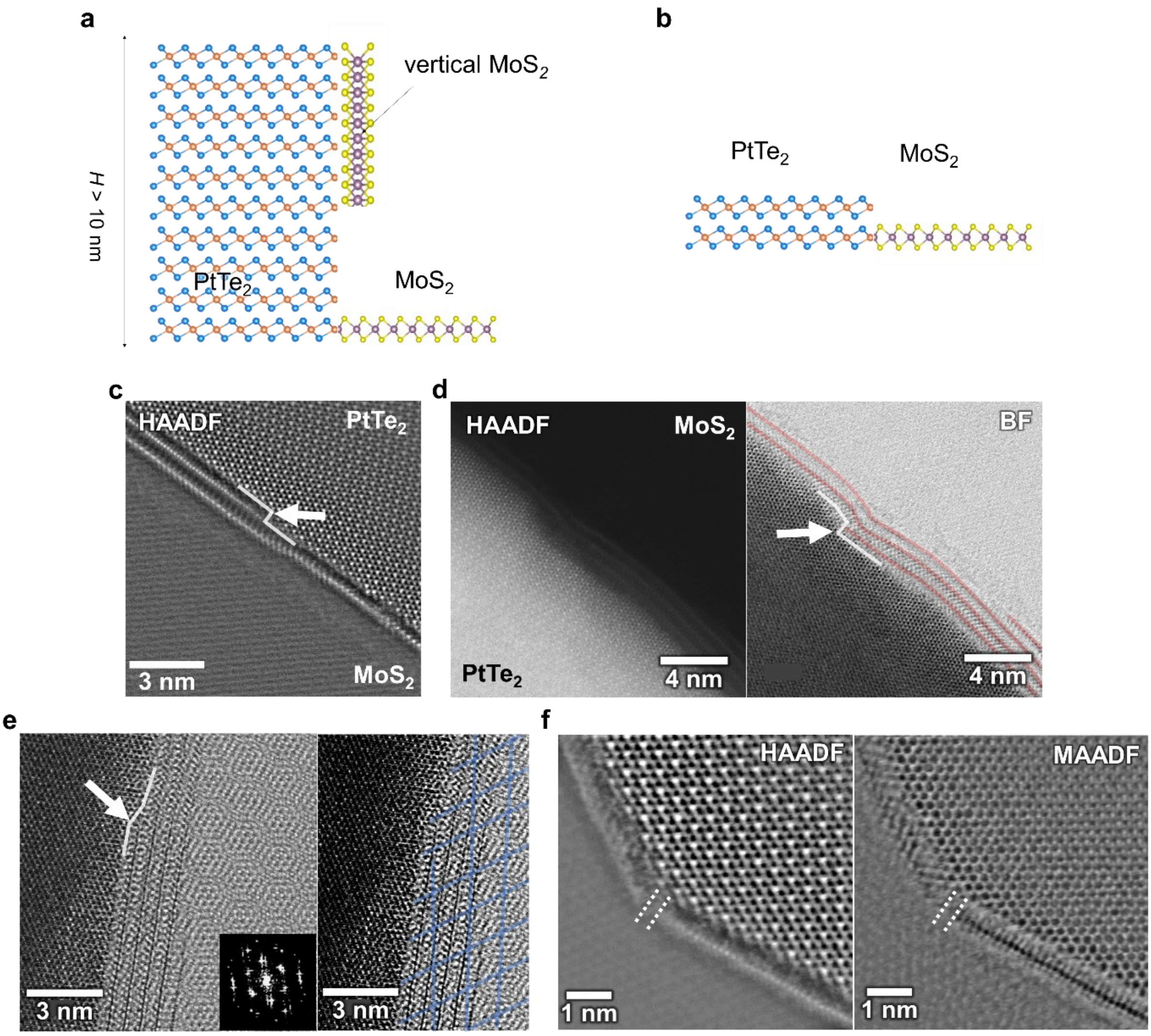

Supplementary Fig. 11. TEM analysis of vertical MoS_2 structure, grown attached to the $PtTe_2$ edge irrespective of the in-plane MoS_2-$PtTe_2$ heterojunction. (a, b) Cross-sectional view of the schematic of $PtTe_2$/MoS_2 heterostructures depending on the thickness of $PtTe_2$. (a) Roughly, $PtTe_2$ thicker than 10 nm might have a synthesized vertical MoS_2 structure at the edge, whereas (b) a thinner one lowers the possibility of the growth of vertical MoS_2. (c-e) STEM images of the different heterostructures with the nucleation of vertical MoS_2 initiated at the step-like edges of the $PtTe_2$ (indicated by an arrow). Since the Moiré patterns of the MoS_2 multilayer appeared underlying vertical MoS_2 (blue lines in (e)), the vertical and lateral MoS_2 layers were individually grown by different growth mechanisms. (Here, the multilayer MoS_2 instead of monolayer MoS_2 was intentionally grown to investigate the Moiré patterns by increasing growth temperature to ~730 °C). (f) (left) HAADF- and (right) medium AADF (MAADF)-STEM images showing the two different vertical MoS_2 layers attached to separate edge faces of $PtTe_2$. The lateral MoS_2 was grown continuously, whereas the vertical layer had a partial disconnection (marked as dashed lines). Furthermore, the lateral and vertical MoS_2 layers were not connected as an integrated atomic structure.

: The existence of a vertical layer attached to some $PtTe_2$ explains how the dangling bond at the edge, in addition to the basal plane, could provide an adequate location for MoS_2 nucleation. However, the vertical MoS_2 was not connected to the lateral MSJ and grew independently (Supplementary Fig. 11a), as concluded by the following observations: (i) First, the Moiré patterns of the lateral MoS_2 multilayer appeared as underlying vertical MoS_2 (Supplementary Fig. 11e). If the structures were connected to each other, the Moiré patterns would not be observed underlying the vertical pattern. (ii) Second, two different vertical MoS_2 layers were separated from each other, whereas the underlying MoS_2 was continuously grown (Supplementary Fig. 11f). Because the vertical structure was isolated, as depicted in Supplementary Fig 11a, our lateral MSJ could have a monolayer-thick interface. The carrier injection path was not related to the vertical structure, rather to the atomically thin MSJ interface; thus, the atomic MoS_2 transistor could be justified.

In our actual structural characteristics, most MSJs possessed laterally stitched components without vertical structures. In addition, we could not find vertically stitched MoS_2 near $PtTe_2$ when the $PtTe_2$ thickness was less than ~10 nm, as is often the case for patterned $PtTe_2$ layers in position-controlled heterostructures (Supplementary Fig. 11b).

We propose the following growth mechanism: The growth of MoS_2 relies on the adsorption of Mo-based precursors (e.g., MoO_x) and their conversion to MoS_2. In thicker flakes, the high density of exposed dangling bonds and the step-like surface of $PtTe_2$ can provide a large surface for the adsorption of vapor-phase MoO_x. Because the edges of the $PtTe_2$ planes are the locations where the basal plane of MoS_2 can grow along the surface (i.e., $(001)_{PtTe2}//(110)_{MoS2}$), the conversion from MoO_x to MoS_2 results in the vertical growth of MoS_2. This growth mechanism resembles those reported previously for Au-MoS_2 (ref. [14]) or MoO_3-MoS_2 core-shell structures[15]. However, in our experiments, the dangling-bond free $PtTe_2$ basal plane prevented the adsorption of MoO_x and the subsequent formation of an out-of-plane overlapped heterostructure. In contrast, for MoO_x adsorbed near the interface between $PtTe_2$ and the SiO_2/Si substrate, the substrate beneath $PtTe_2$ could promote the in-plane lateral epitaxy of MoS_2 whereas $PtTe_2$ provided heterogeneous nucleation sites.

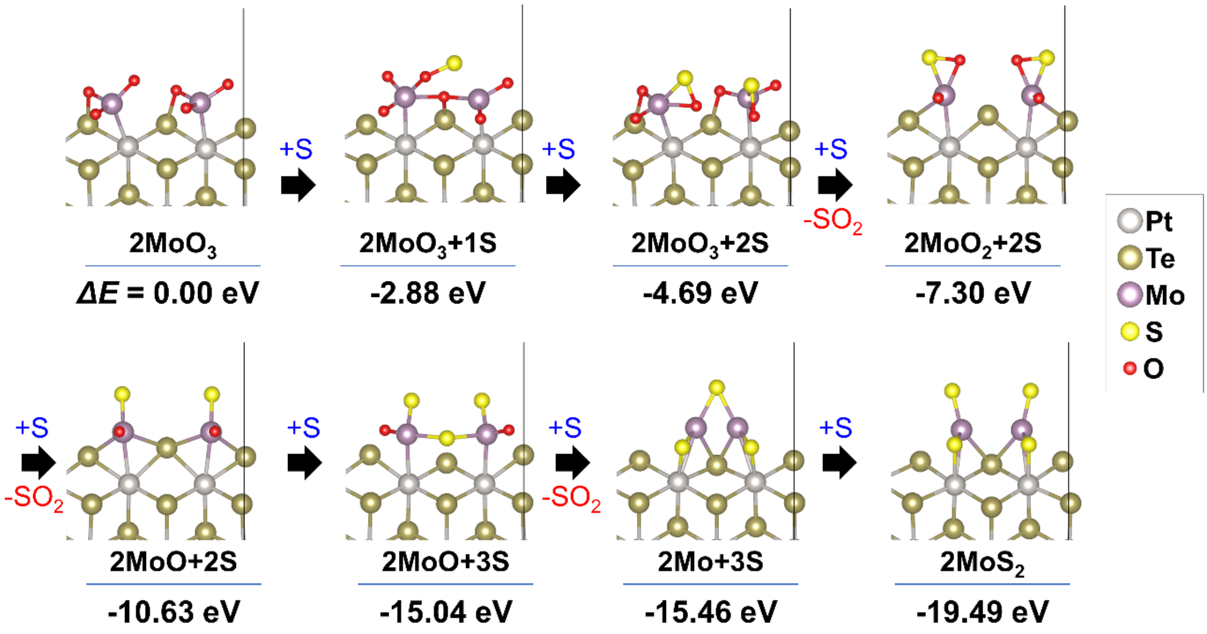

Supplementary Fig. 12. Top-view atomic configurations of MoO_x and S dimers during MoS_2 growth at $PtTe_2$ edge. The value of ΔE below each illustration represents the energy of the process relative to that of the $PtTe_2$ + $2MoO_3$ atomic structure. Gas-phase S tends to react with MoO_x. This results in the desorption of SO_2 and the promotion of MoO_x reduction. The final MoS_2 structure rather than MoO_x is the most stable structure because the calculated ΔE of the former is the smallest (~ -19.49 eV) among the steps.

: The initial atomic configuration of the $PtTe_2$ edge was assumed to have 50 % Te coverage, and the termination of the as-grown $PtTe_2$ flake was confirmed by STEM study (Supplementary Fig. 3). As shown in Supplementary Table 2, the relative energy (ΔE) of the possible intermediates of MoO_x and S adsorbents attached to the $PtTe_2$ edge were evaluated, indicating the relative stability of each atomic structure. Reactive, gas-phase S atom is likely bound to MoO_3 dimers attached to the edge of $PtTe_2$ (ΔE < -2.88 eV), and O in MoO_3 tends to desorb since it reacts with additional S forming SO_2. This is owing to the exothermic processes in the single-layer model calculations. The similar reduction process for MoO_{3-x} repeated to form the most stable structure of MoS_2, because its energy relative to the initial atomic structure was substantial (ΔE = -19.49 eV). A previous study for the structural evolution of the MoS_2-Mo_2C interface[16] reported that the exothermic (ΔE < 0 eV) and endothermic (ΔE > 0 eV) steps were mixed during the interface generation steps in the DFT calculations. In contrast, the lateral epitaxy of MoS_2 to the $PtTe_2$ edge interface in this study was driven by multiple exothermic steps, indicating that the reaction is relatively favorable.

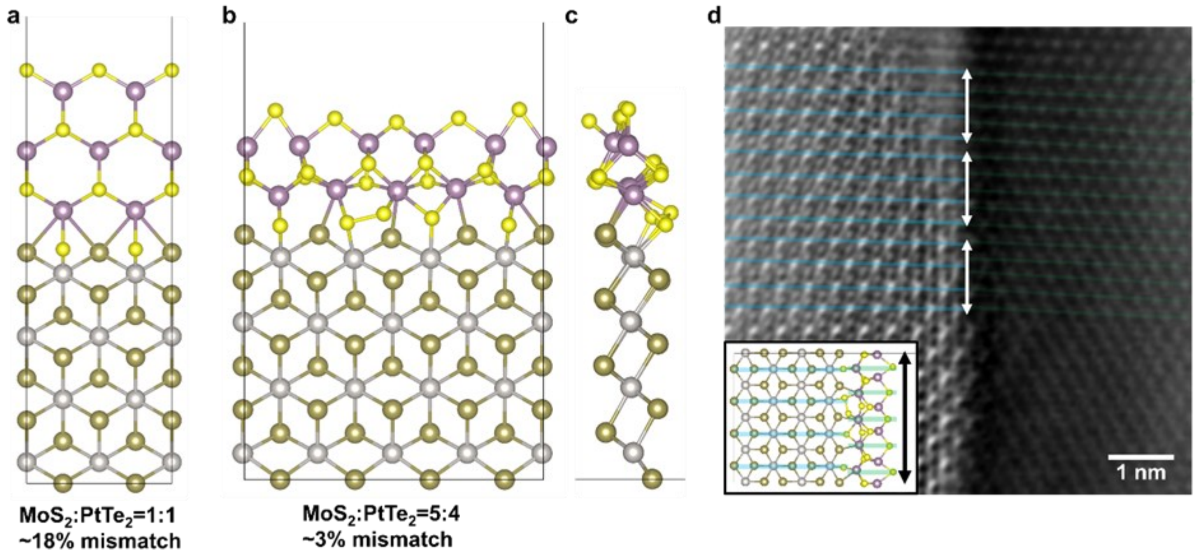

Supplementary Fig. 13. Junction formation in heterostructure with minimized lattice mismatch. (a) Schematic of $PtTe_2$-MoS_2 MSJ realized by one-to-one matching of each unit cell. The lattice mismatch of ~18% between MoS_2 and $PtTe_2$ may lead to substantial mechanical strain. (b) Top- and (c) side-view atomic structures of $PtTe_2$-MoS_2 MSJ with the smallest unit interface mismatch of ~3% calculated using DFT. (d) HAADF-STEM image showing the formation of the interface in the lateral MSJ with four $PtTe_2$ and five MoS_2 periodic unit cells in each interfacial unit cell. The inset shows the corresponding schematic atomic arrangement at the interface.

: Supplementary Figures 13b and c present the calculated atomic structures of the $PtTe_2$-MoS_2 heterointerface consisting of the five MoS_2 and four $PtTe_2$ unit cells. The MoS_2 and $PtTe_2$ lattices are continuous across the interface along the <100> direction while forming Mo-Te and Pt-S bonds. The S atom is situated between the two Te atoms of the $PtTe_2$ termination in the top-view structure in Supplementary Fig. 13b, and then the MoS_2 grows continuously (the calculated relative energy also validates each reaction process conducted for MoS_2 dimers in Supplementary Fig. 12a.). Although the atomic termination of MoS_2 was strained to form bonds with $PtTe_2$, there were no dangling bonds at the MoS_2/$PtTe_2$(010) interface, as shown in Figure R3a, indicating a semi-coherent interface. The periodic interfacial cell repeatedly forms the lateral MSJ heterostructure, as shown in the three periodic cells displayed as white arrows in the STEM image in Supplementary Fig. 13d. The regular matching of the atomic sites indicates their (semi-)coherency. The TEM diffractograms of the $PtTe_2$-MoS_2 heterostructure (Figs. 1i and 3h) also demonstrate a well-defined orientational consistency between the 2D materials in two different directions (i.e., epitaxial relation of the (110) and (100) planes), which could not be realized in an incoherent interface with large misfits.

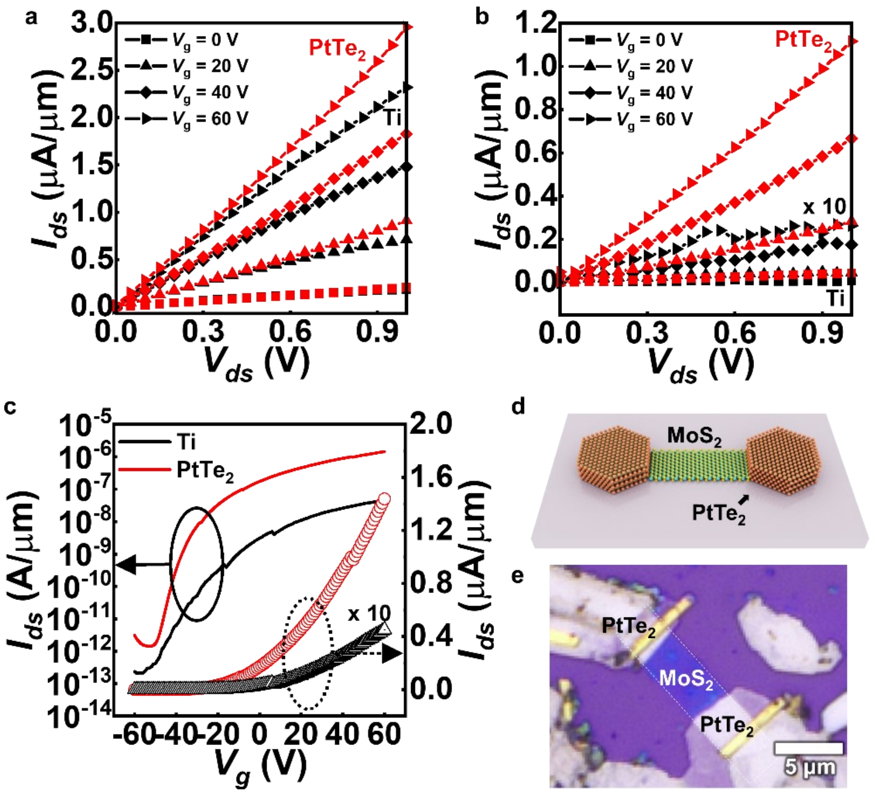

Supplementary Fig. 14. Room-temperature electrical properties of 2D-2D $PtTe_2$-MoS_2 MSJ FETs with (a) asymmetric and (b–d) symmetric contacts. (a) Output curves (I_{ds}-V_{ds}) of monolayer MoS_2 MSJ FETs where the electrons are injected from $PtTe_2$ (red) and Ti (black); this is the same device represented in Fig. 2g. (b–d) Electrical performance of MoS_2 FETs fabricated with symmetric $PtTe_2$ edge contacts. Ti-contact MoS_2 FETs were also fabricated and evaluated for comparison. (b) Output and (c) transfer characteristics of representative device. (d) Schematic of device with symmetric $PtTe_2$ contacts. (e) OM image of representative device. Performance improvement using $PtTe_2$ edge contact was confirmed for the MoS_2 device with symmetrical contact. The device showed a maximum μ_{FE} of up to ~15.8 $cm^2V^{-1}s^{-1}$, higher than those from asymmetrically contacted devices in Fig. 2g, h.

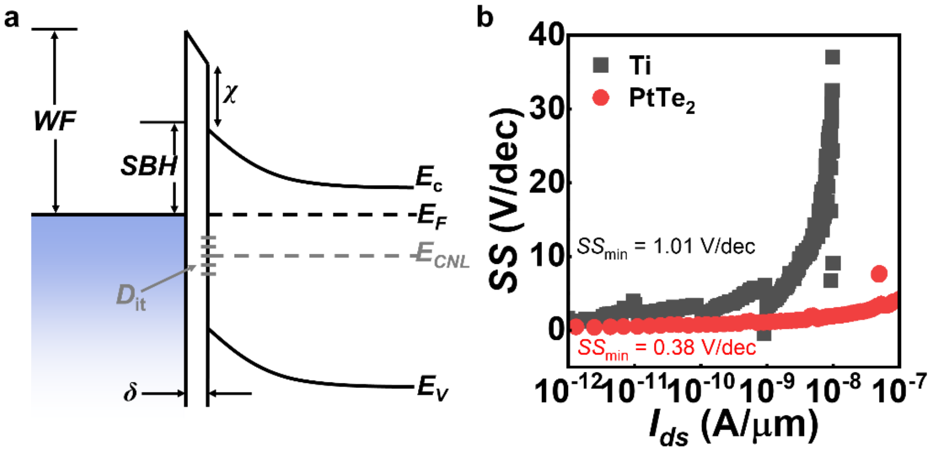

Supplementary Fig. 15. Influence of interfacial defects on FLP effect in 2D MSJs. (a) Energy band diagram of a metal-MoS_2 junction containing an interfacial layer with a thickness of *δ*. (b) Subthreshold swing (SS) versus I_{ds} plot calculated from the data set in Figure 4c. The minimum SS value (SS_{min}) measured at 300 K was improved from 1.01 V dec^{-1} to 0.38 V dec^{-1} in the edge-contact $PtTe_2$. The reduced SS_{min} values of the $PtTe_2$-MoS_2 MSJ FETs suggests a smaller contact interfacial trap density (D_{it}) in these FETs.

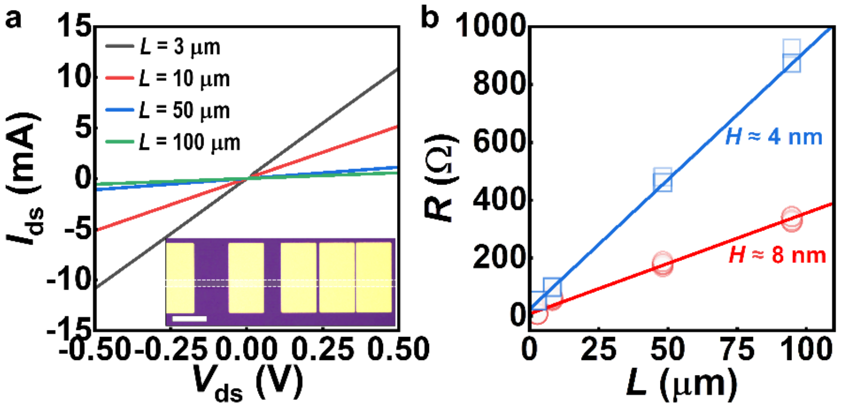

Supplementary Fig. 16. TLM measurements for Ti/Au contacted two-terminal $PtTe_2$ devices. (a) Representative I_{ds}-V_{ds} characteristics of $PtTe_2$ devices with various channel lengths (L). The inset shows the OM image of a representative TLM device (scale bar: 100 μm). (b) TLM plots for Ti/$PtTe_2$ devices with different channel thicknesses (H). Each data point corresponds to the total resistance (R) of one device. Linear plots were fitted to the different TLM sets to extract the average $2R_c$ values corresponding to the y-intercepts. The $PtTe_2$ channel here was synthesized using the method described in Fig. 3a.

: We extracted the R_c values between the Ti/Au and $PtTe_2$ thin films (~4 and ~8 nm) using the TLM, as shown in Supplementary Fig. 16. The average R_c values were ~230 ± 20 and ~120 ± 30 Ω·μm for the ~4 and ~8 nm-thick $PtTe_2$ films, respectively. Compared to the TLM-driven R_c of the $PtTe_2$-MoS_2 MSJ (168 ± 127 kΩ·μm; Fig. 4f), the influence of R_c at the Ti/$PtTe_2$ interface was trivial (~0.13%). The estimated R_c of the Ti/$PtTe_2$ interface was almost equal to the lowest value obtained for 3D metal/vdW metal interfacial systems and smaller than that of the most widely used vdW metal, graphene. Efficient carrier transfer from a 3D metal to a vdW metallic layer is dependent on the differences between their (i) DOS and (ii) WFs because these quantities determine the DOS-limited dipole layer at the interface[17,18]. For example, the negligible DOS in the vicinity of the Dirac point in graphene results in a longer charge-transfer region at the interface (i.e., increased screening length)[19]. Compared to graphene, the anisotropically contacted electron and hole pockets in the Type II Dirac semimetal $PtTe_2$ produce a finite DOS around the Dirac point[20] with a higher carrier concentration (> 6×10^{22} cm^{-3})[21], which led to better transport at the 3D metal/$PtTe_2$ contact interface. Furthermore, the small WF difference between $PtTe_2$ and Ti (~200 meV) resulted in a smaller potential difference in the dipole layer. We also note that the wetting property of Ti evaporated on the $PtTe_2$ surface can reduce the equilibrium separation between $PtTe_2$ and Ti, which can be regarded as being smaller than the $PtTe_2$ vdW gap (~2.5 Å). Given the negligible impact of the vdW gap with a small separation (~3.3 Å) in Au/graphene[19], the tunnel barrier in our Ti/$PtTe_2$ system might also be insignificant. The strong interlayer interaction of $PtTe_2$, as indicated by its vdW gap (~2.5 Å) being smaller than the intrinsic layer thickness (~2.7 Å)[21], can result in an invariance of the contact resistance properties with respect to variations in the thickness similar to that in WTe_2 (ref. [22]).

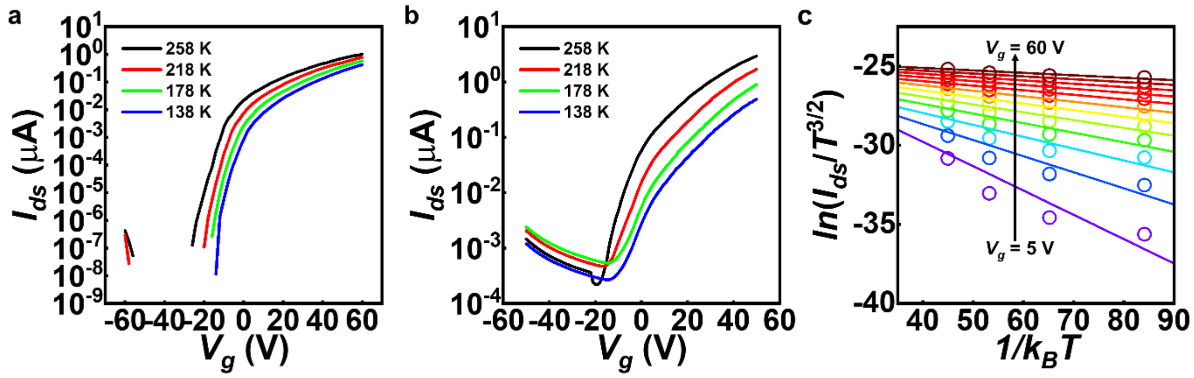

Supplementary Fig. 17. Extraction of SBH of (a) $PtTe_2$-MoS_2 and (b, c) Ti/MoS_2 MSJs by using the thermionic emission model. (a, b) Representative *T*-dependent transfer characteristics (I_{ds}-V_g) of monolayer MoS_2 MSJ FETs with (a) $PtTe_2$ flakes and (b) Ti symmetric contact. (c) Corresponding Arrhenius plot of Ti/MoS_2 MSJ in (b).

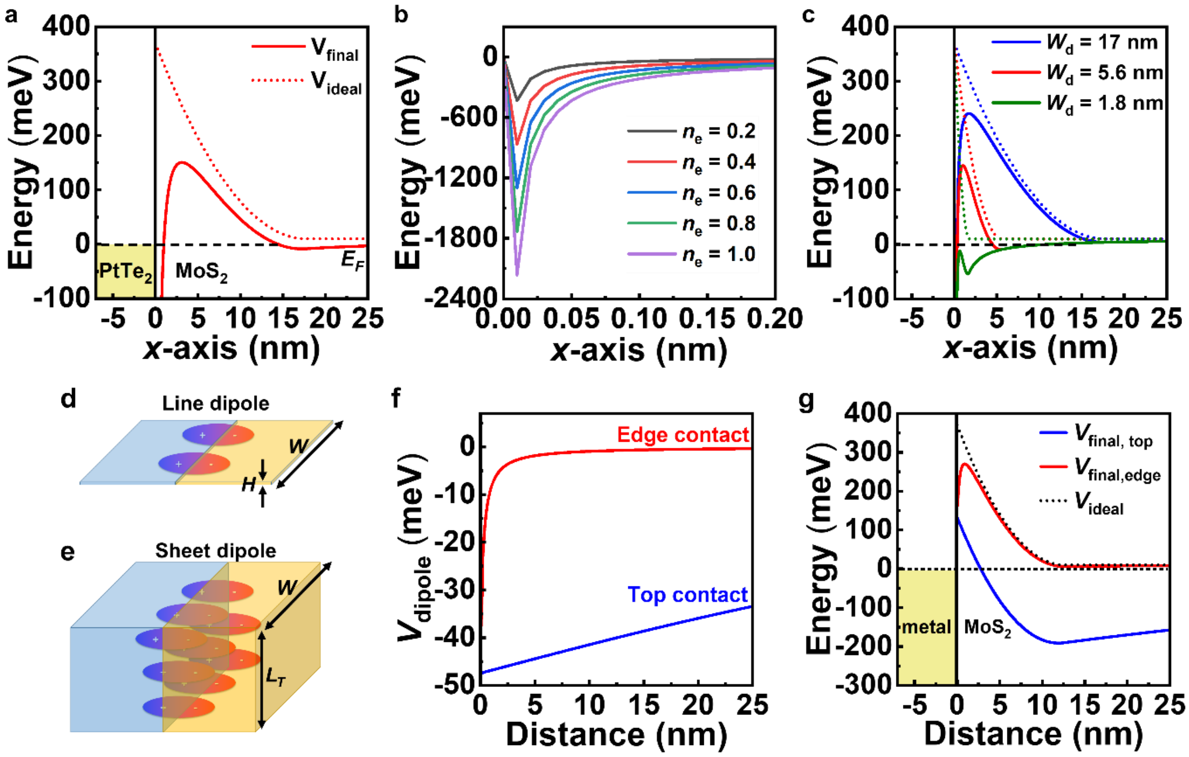

Supplementary Fig. 18. Effect of metal-induced gap state (MIGS) produced by dipole potential (V_{dipole}) on energy profiles of lateral $PtTe_2$-based heterostructure with a MoS_2 monolayer. (a) Calculated potential of the lateral heterostructure depicting the ideal potential (V_{ideal}, dashed curves) and the potential modified by the distributed dipoles at the interface ($V_{final} = V_{ideal} + V_{dipole}$, solid curves). These potential profiles were estimated by assuming the high density of pinning charges (~51 C/m^2) and typical barrier profiles for the few-layered MoS_2. (b) The calculated dipole potential (V_{dipole}) as a function of the number of pinning charges per S atom (n_e)[23-25]. As the pinning charge density increases for edge contact ($\sigma = 4q/(a \cdot c) \times n_e$)[23], the extent of V_{dipole} increases. (c) Plot showing the substantial effect of the carrier concentration (n_{3D}) and depletion regions (W_d) on the modulating V_{ideal} (dotted line) even with the same V_{dipole}. The W_d of ~1.8, ~5.6, and ~17 nm was calculated by the consideration of n_{3D} of ~5 × 10^{19}, ~5 × 10^{18}, and ~5 × 10^{17} cm^{-3}, respectively. (d, e) Schematics of contact geometry-dependent dipoles: (d) line dipoles in an edge contact and (e) sheet dipoles in a vertical contact. (f) Calculated V_{dipole} of top and edge contacts for an ideal case with the same pinning charges per sulfur ($n_e = 1$). (g) Calculated V_{ideal} and V_{final} modified by different V_{dipole} configurations depending on the contact dimensions. The same contact potential (V_p) was assumed in the calculation to compare the effect of the two contact geometries on V_{final}.

: The perfect FLP-free formation of SBH for the $PtTe_2$-MoS_2 MSJ can be challenging, primarily attributed to the modified potential of the heterostructure by dipole-induced FLP,

following an approach suggested by refs. [23-25]. In every MSJ system, an electron density rearrangement at any MSJ happens attributed from the overlapping wavefunctions of metal and semiconductor; thus, forming so-called metal-induced gap states (MIGS)[26,27]. According to previous reports for top contacts to 2D semiconductors, the charge redistribution modifies SBH even without the chemical reaction between 3D metal and MoS_2, as the XPS studies for Pd on the MoS_2 surface revealed that a considerable band bending and Pd's WF decrease from 5.55 eV to 4.84 after its deposition[28]. This dipole-induced FLP can be alleviated by introducing an interlayer between the metal and semiconductor, where the MIGS are likely to degrade as interlayer thickness increases[29,30].

Accordingly, in every MSJ, the transfer of carriers between the interface state and metal can result in potential formation due to the dipoles, which modify the ideal band-bending profile ($V_{final} = V_{ideal} + V_{dipole}$; Supplementary Fig. 18a)[23-25]. The strong covalent bonds between $PtTe_2$ and MoS_2 can also induce a charge transfer (recalling the XPS peak shift of Pt-Te bindings in Supplementary Fig. 6), resulting in the formation of a dipole potential (V_{dipole}), although the distribution was confined to few-layered MoS_2. We calculated V_{ideal} as a function of the distance (x) from the junction interface toward the 2D semiconductor by using Poisson's equation[23]:

$$qV_{ideal}(x) = \frac{qV_P(x-W_d)^2}{W_d^2} + \left(E_c - E_f\right) \qquad \text{(S1)}$$

where qV_p is the contact potential calculated by $qV_p = (WF - \chi) - (E_c - E_f)$ and W_d is estimated by the following expression:

$$W_d = \sqrt{\frac{2\varepsilon V_P}{qn_{3D}}} \qquad \text{(S2)}$$

The WF for the calculation for $PtTe_2$ was ~4.65 eV (Supplementary Fig. 2h), χ is ~4.28 eV and ε was ~4 for monolayer MoS_2 (refs. [5,31]). As for the computations in Supplementary Fig. 18a, we used the values of 10 meV for E_c-E_f, and n_{3D} of ~10^{18} cm^{-3}, which is validated for the highly n-doped MoS_2 FET with a threshold voltage (V_{th}) of ~14.6 V for our devices (Supplementary Fig. 14) by considering the 2D carrier density of a 300-nm-thick SiO_2 dielectric layer ($n_{2D} = C_{ox}(V_g - V_{th})/q$, where $V_g = 0$ V). Regarding the potential established due to the dipole charges (V_{dipole}), the parallel rectangular contact width ($W = \infty$) and thickness (H = 0.7 nm for monolayer MoS_2) of the edge contact was considered with a separation of d (~0.32)[23,81] as expressed by:

$$qV_{dipole}(x, H) = -q\left(\int_{-\frac{d}{2}}^{x} E \cdot dx + \int_{\frac{d}{2}}^{x} E \cdot dx\right) \qquad \text{(S3)}$$

Here, E(x) is the electrical field caused by dipoles at the heterostructure as follows:

$$E(x) = \frac{\sigma x}{4\pi\varepsilon}\int_{-\frac{W}{2}}^{\frac{W}{2}}\int_{-\frac{H}{2}}^{\frac{H}{2}} \frac{1}{(x^2+y^2+z^2)^{\frac{3}{2}}}\, dzdy \qquad \text{(S4)}$$

The heavy n-type doping of MoS_2 decreases both the conduction band offset with respect to the Fermi level (E_C-E_f) and the depletion width (W_d). This also results in greater carrier injection because the effect of V_{dipole} on V_{final} increases as W_d decreases (Supplementary Fig. 18b). Since monolayer MoS_2 was grown by CVD, the high-temperature-driven vacancies[32] and the large trap density of the substrate[33] may cause the n-doping of the MoS_2, decreasing both E_C-E_f and W_d, finally instigating FLP. We also suppose that the transported carriers preferred to pass through the SBH of the multilayer MoS_2 interface rather than the monolayer because of the higher electron affinity ($\chi_{ML} \approx 4.45$ eV (ref. [5])) and higher carrier density (n_{3D}), lowering both V_{ideal} (from ~370 meV to ~200 meV) and W_d (from ~17 nm to ~11 nm) calculated by Equations S1 and S2.

However, compared to the 3D vertical contacts, V_{dipole} decays more rapidly away from the interface. This is because the pinning charges at the 1D-like interface at the edge contact are modeled as dipoles in two-parallel rectangles with the width W and MoS_2 thickness H, as depicted in Supplementary Figure 18d. In contrast, the top contact has a geometry with the width W and carrier transfer length L_T. Therefore, away from the interface, the conventional Schottky-Mott limit alignment, that is, partial depinning of the Fermi level, is recovered in the edge contact[5,23,24,34-36]. We qualitatively show the dimensional effect of the edge and top contacts on V_{dipole} and V_{final}. The different decay rates of V_{dipole} modify the total V_{final} depending on the contact geometry and reduce the influence of V_{ideal} on V_{final} at the edge interface.

The weak FLP contributed by the reduced dimensionality of the interface is also implied in the Szes' model (Supplementary Note 2). According to the model, a single-atom-thick interfacial layer (i.e., interface thickness, $\delta \approx 0$) yields a pinning factor (S) of ~1, thereby weakening the FLP. Therefore, regardless of the coherence/incoherence of the heterointerface, edge contact can provide a rather FLP-free interface compared to the 3D vertical contacts, as proved by the previous reports for the incoherent edge interface fabricated by the etching of the 2D semiconductor edge and *in-situ* 3D metal deposition[23,35].

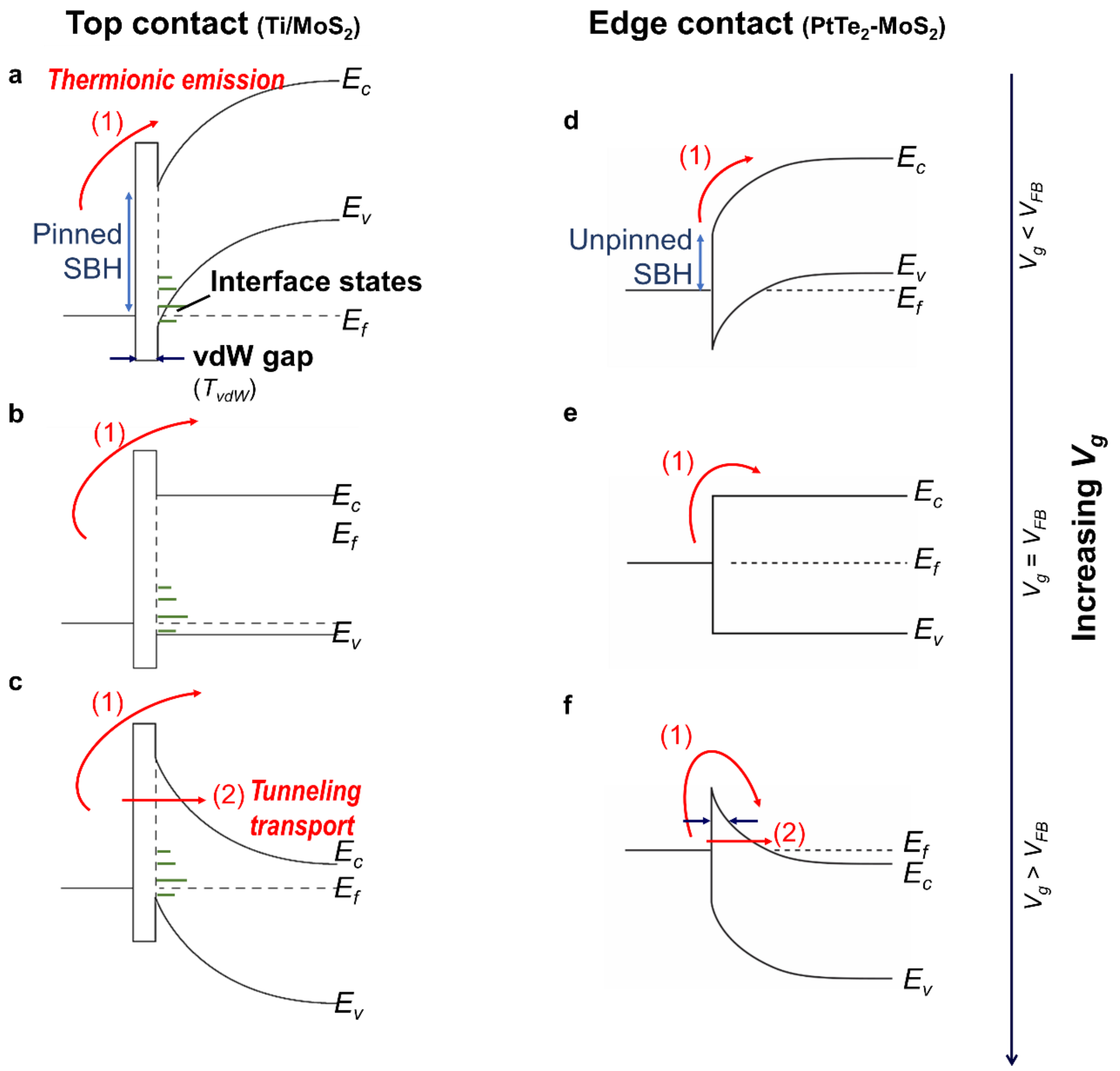

Supplementary Fig. 19. Band diagrams depicting the charge transport mechanism. Electron injection through the Schottky barrier in the (a–c) 3D/2D Ti/MoS_2 MSJ, and (d–f) lateral 2D-2D $PtTe_2$-MoS_2 interface shown for various *n*-doping levels of the channel layer induced by induced V_g: (a, d) $V_g < V_{FB}$, (b, e) $V_g = V_{FB}$, and (c, f) $V_g > V_{FB}$. (1) and (2) denote thermionic emission and tunneling transport, respectively.

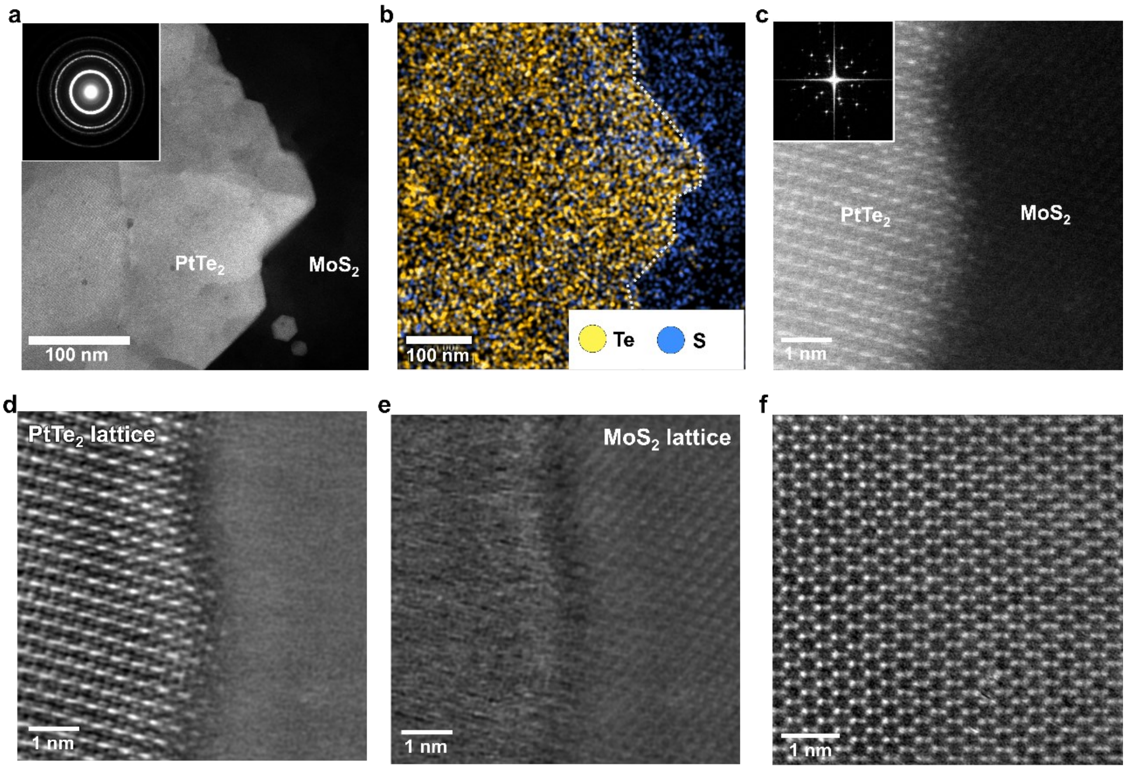

Supplementary Fig. 20. TEM image of heterostructure with patterned $PtTe_2$ film (H ≈4 nm) and monolayer MoS_2. (a) Low magnification HAADF-STEM image of heterostructure. Inset shows a representative SAED pattern of heterostructure, demonstrating polycrystalline nature. (b) Corresponding EDS mapping of crystals in (a) for Te and S. The slightly mixed signals at the $PtTe_2$ region are attributed to measurement error (e.g., noise signals) owing to the thickness difference. (c) HAADF-STEM image of the MoS_2-$PtTe_2$ heterostructure. The inset shows the corresponding FFT patterns. (d, e) Corresponding inverse FFT images for (d) the $PtTe_2$ and (e) MoS_2 lattices. The FFT images show that the MoS_2 layer was fully connected to $PtTe_2$. (f) Atomic-resolution HAADF-STEM image of the MoS_2 surface far from the $PtTe_2$ interface. In Fig. 3e, the MoS_2 surface appears somewhat indistinct compared to $PtTe_2$, because of the difficulties in focusing on both surfaces simultaneously due to the thickness difference between $PtTe_2$ and MoS_2 (Figure 3 was taken with the focus on $PtTe_2$.). In addition, because of the difference in Z contrast between Pt-Te and Mo-S, the intensity of Mo-S was not distinct in the HAADF-STEM images.

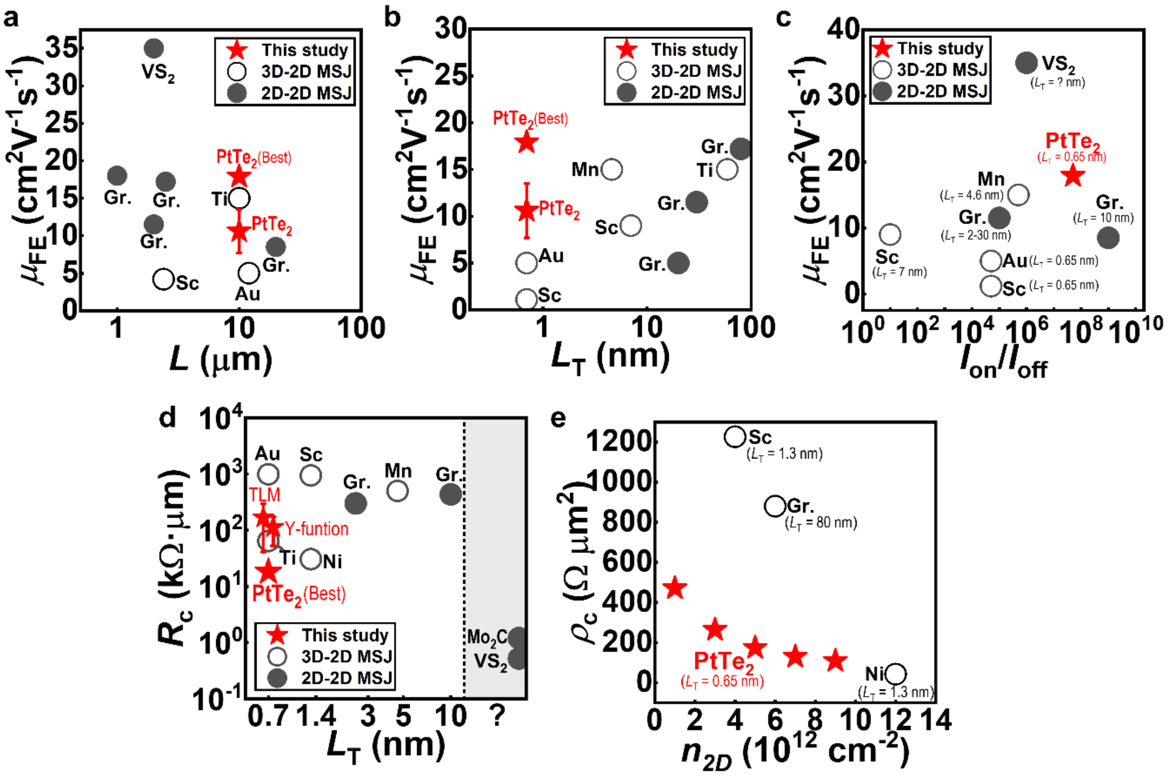

Supplementary Fig. 21. Comparison of field-effect mobility (μ_{FE}) and contact resistance of $PtTe_2$-MoS_2 MSJ FETs with other reported values. (a, b) Two-terminal μ_{FE} values of lateral MoS_2 MSJ FETs with $PtTe_2$ edge contacts and different (a) channel lengths (L) and (b) transfer lengths (L_T). The corresponding values for reported edge-contact 3D-2D MSJs (e.g., Sc (ref. [37]), Ti (ref. [35]), Au (ref. [24]), and Mn (ref. [23])) and lateral 2D-2D MSJs (e.g., graphene (Gr.) (ref. [38-41]), and VS_2 (ref. [42])) are shown for comparison. (c) Comparison of μ_{FE} and I_{on}/I_{off} values for MSJ FETs with an ultrashort L_T (<10 nm). For fair comparison, the highest values reported in each paper[23,24,37-42] were utilized. We also note L_T values reported in each paper because μ_{FE} and R_c tend to be decrease when L_T is increased, owing to the weakened current crowding effect. (d) Evaluation of the R_c of our $PtTe_2$ edge contact with previously reported ones for MoS_2-based MSJ FETs with an L_T less than 10 nm, obtained using various metals such as Ti/Au (ref. [35]), Sc (ref. [37]), Au (ref. [24]), Ni (ref. [43]), Mn (ref. [23]), Gr. (ref. [38-41]), VS_2 (ref. [42]), and Mo_2C (ref. [16]). The L_T of Mo_2C and VS_2 was unidentified in each paper[16,42]. (e) ρ_c of $PtTe_2$-MoS_2 MSJ FETs depending on the n_{2D}. We also demonstrate ρ_c of other reported devices that used lateral contacts such as Gr. (ref. [39]), Sc (ref. [37]), and Ni (ref. [43]). For more details on the performance evaluation, see Supplementary Note 3.

: Although our $PtTe_2$ edge-contact MoS_2 MSJ FET had the shortest L_T with the thinnest channel among the reported lateral/edge MSJ FETs, it showed the lower R_c (Supplementary Fig. 21d), thus, resulting in higher two-terminal μ_{FE} and I_{on}/I_{off} values than those reported in other papers[23,24,37-42] (Supplementary Fig. 21c and Supplementary Table 5).

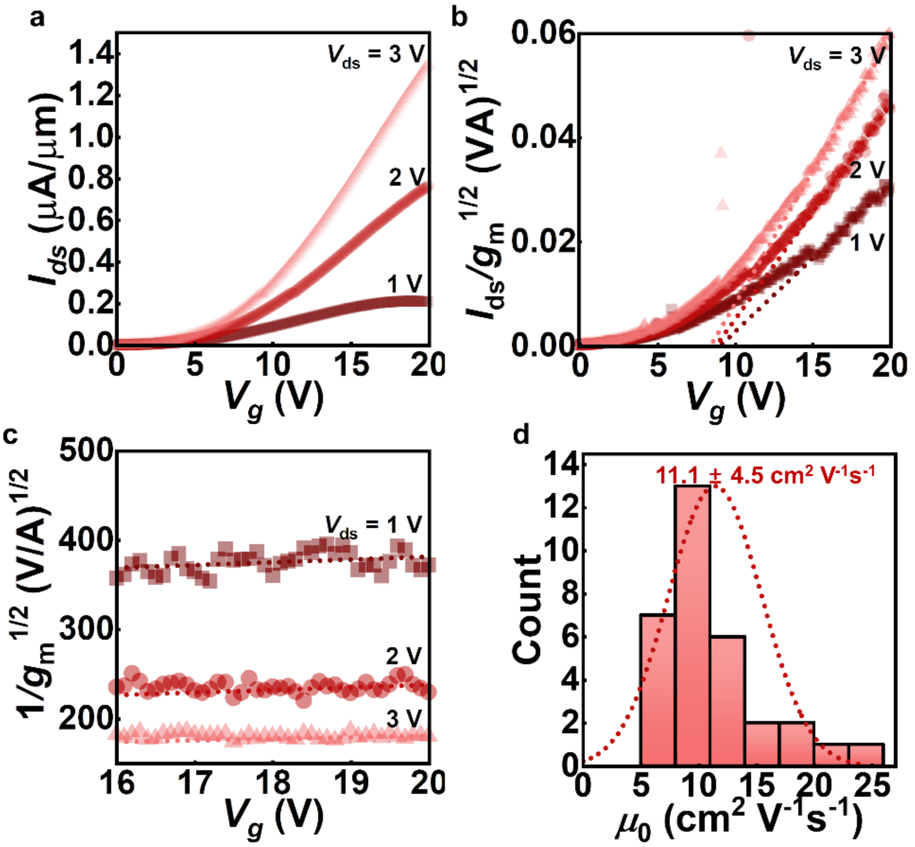

Supplementary Fig. 22. *Y*-function-derived intrinsic carrier mobility and contact resistance of $PtTe_2$-MoS_2 MSJ FETs. (a) Representative transfer characteristics of $PtTe_2$-MoS_2 MSJ FETs plotted on a linear scale, showing the sub-linearly increased I_{ds} at the inversion regime. (b) Plots of Y-function *vs.* V_g from which the intrinsic carrier mobility (μ_0) is extracted as the slope (S_1) of the linear fits. (c) Plot of $1/(g_m)^{1/2}$ *vs.* V_g in the strong accumulation regime. (d) Histograms of μ_0 extracted by the Y-function approach. The lowest R_c (ρ_c) obtained in this study is ~18.2 kΩ·μm (~11.8 Ω·μm^2) and the average value reached ~113.0 ± 60.3 kΩ·μm (~73.5 ± 39.2 Ω·μm^2), which is comparable to that calculated by TLM (Fig. 6g). The extracted μ_0 approached ~23.6 cm^{2}V^{-1}s^{-1} (~11.1 ± 4.5 cm^{2}V^{-1}s^{-1} on average), a value higher than the two-terminal μ_{FE} (~10.6 ± 2.9 cm^{2}V^{-1}s^{-1}) because of the circumvention of its underestimation associated with the presence of a contact barrier.

: To ensure the accuracy of the R_c calculated by TLM, we extracted R_c using the Y-function method, which has been successfully applied in several studies on MoS_2 FETs[40,44,45]. Compared to TLM, this approach allows the extraction of the performance parameters, despite the possibility of device-to-device variations in the R_c and R_{sh} of MoS_2. As the R_c induced by the Schottky barrier can result in additional voltage drops at V_{ds} (=V_{ds}-2$I_{ds}R_c$) and V_g (=V_g-$I_{ds}R_c$), the ideal expression of I_{ds} in the accumulation regime can be modified as;

$$I_{ds} = \mu_0 C_{ox} \frac{W}{L}\left[\left(V_g - I_{ds}R_c\right) - V_{th} - \frac{(V_{ds}-2I_{ds}R_C)}{2}\right] \times (V_{ds} - 2I_{ds}R_c) \qquad \text{(S5)}$$

where μ_0 is the intrinsic carrier mobility of the device, which is independent of the contact property. The effect of R_c on I_{ds}, therefore, results in a sublinear increase in I_{ds} with V_g in the inversion region, as shown in Supplementary Fig. 22a. Furthermore, the effective mobility of the device is attenuated by a factor of $\theta = \theta_0 + 2R_c\mu_0 C_{ox}(W/L)$ (where the first-order mobility attenuation coefficient, θ_0, is negligible in the strong inversion region)[45]; this in turn enables the modification of Equation S5 to:

$$I_{ds} = \left(\frac{\mu_0}{1+\theta\left(V_g - V_{th} - \frac{V_{Ds}}{2}\right)}\right) C_{ox}\frac{W}{L}\left(V_g - V_{th} - \frac{V_{ds}}{2}\right) V_{ds} \qquad \text{(S6)}$$

Equation S6 can be modified to the relationship of the commonly accepted Y-function method from the classification of the transconductance ($g_m = \partial I_{ds}/\partial V_g$) and Y-function ($Y = I_{ds}/(g_m)^{1/2}$), as follows:

$$Y = \frac{I_{ds}}{\sqrt{g_m}} = \left(\mu_0 C_{ox} V_{ds}\frac{W}{L}\right)^{\frac{1}{2}}\left(V_g - V_{th} - \frac{V_{ds}}{2}\right) \qquad \text{(S7)}$$

This expression allows the extraction of μ_0 and V_{th} from the slope (S_1) and x-intercept of the Y-function plot (Y *vs.* V_g), respectively (Supplementary Fig. 22b). Furthermore, the relationship between V_g and $1/(g_m)^{1/2}$ can be used to calculate the attenuation factor θ, using the modified expression of Equation S6:

$$\frac{1}{\sqrt{g_m}} = \left(\frac{L}{\mu_0 C_{ox} V_{ds} W}\right)^{\frac{1}{2}}\left[1 + \theta\left(V_g - V_{th} - \frac{V_{ds}}{2}\right)\right] \qquad \text{(S8)}$$

We can also calculate R_c using the slope S_1 (Y *vs.* V_g plot) and slope S_2 of the plot of $1/(g_m)^{1/2}$ vs. V_g in the strong inversion region (Supplementary Fig. 22c) using the following expression:

$$R_c = S_2 V_{ds}/2S_1 \qquad \text{(S9)}$$

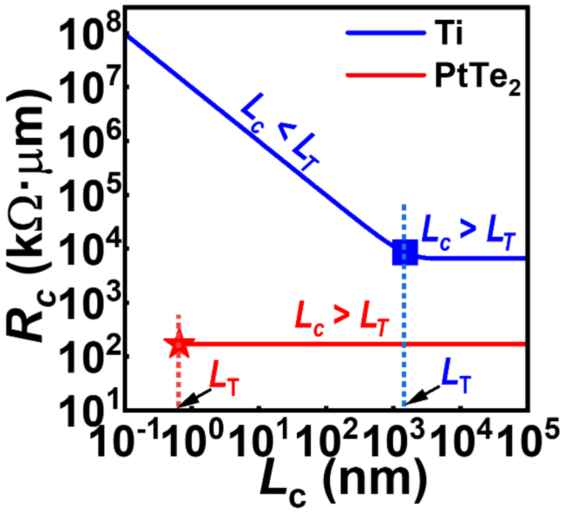

Supplementary Fig. 23. R_c of Ti/MoS_2 MSJ FET depending on the L_c calculated based on our data and a transmission line model[46,47]. The scaling in L_c of Ti/MoS_2 MSJ FET can increase the R_c because of crowding effect of carrier (blue, $L_c < L_T$). Compared to 3D vertical contacts, edge contact prospectively affords contact scalability down to the physically obtained L_T of ~0.7 nm (red).

Supplementary Table 1. Comparison of growth methods to obtain lateral edge-contacted 2D vdW MSJ by the two-step CVD process. Most studies on the lateral MSJs prepared using CVD rely on the growth of vdW metals after the preparation of vdW semiconductors because the typical vdW semiconductor (e.g., WS_2, $MoSe_2$) requires a relatively high growth temperature (700-800 °C) compared to vdW metals (e.g., 600 °C for NbS_2 or VSe_2)[48-50]. Although graphene can be exploited to create nucleation sites for MoS_2 afterward[38,39,51], the preparation of CVD-grown graphene requires wet-transfer onto a target substrate which is a cumbersome process producing polymer-based impurities that could act as nucleation sites on the graphene basal surface[39]. Most importantly, the significant lattice mismatch between graphene and the vdW semiconductor (> 25%) hinders lateral epitaxial growth, resulting in a vertically overlapped junction[39,51] instead of pure edge contact. Furthermore, the inertness of the $PtTe_2$ surface without the in-plane alloying is a phenomenon not observed in the studies on synthetic VS_2-MoS_2 (ref. [42]) and graphene-MoS_2 (refs. [39,51]), owing to the surface roughening of VS_2 under a high-temperature oxidative atmosphere[42] and transfer-induced polymer residues[39,51].

**Growth order**	**Materials**		**Growth *T* of the 2nd CVD**	**Basal plane quality**	**Use of growth promoters**	**Ref.**
	**1st CVD**	**2nd CVD**				
Metal ⇒ Semiconductor	**$PtTe_2$**	**MoS_2**	**700 °C**	**Avoided thermal budget processes or in-plane alloying**	**Possible to use NaCl**	**This study**
	VS_2	MoS_2	650 °C	Formation of in-plane alloying	N/A	Ref. 42
	Gr. *(transferred)*		650 °C		N/A	Ref. 51
			850 °C		N/A	Ref. 38
Semi-conductor ⇒ Metal	MoS_2	Mo_2C	820 °C	Thermal budget effects on the active layer of semiconductors	Cu foil	Ref. 16
		NbS_2	600 °C		N/A	Ref. 49
	WS_2	NbS_2	790 °C		NaCl	Ref. 52
	WSe_2	VSe_2	600 °C		N/A	Ref. 48
		WTe_2	700 °C		KI	Ref. 53
	2H-$MoTe_2$	1T'-$MoTe_2$	530 °C		N/A	Ref. 54

Supplementary Table 2. Calculated relative energy (*ΔE*) for every possible atomic combination during the vaporization process of MoO_3 and S. The adsorption of MoO_3 to the edge of $PtTe_2$ was set as an initial process with a *ΔE* = 0 eV.

**Atomic structure**	***ΔE* (eV)**
$PtTe_2$+2MoO_3	0.00
$PtTe_2$+2MoO_3+S	-2.88
$PtTe_2$+2MoO_3+2S	-4.69
$PtTe_2$+2MoO_2	-1.80
$PtTe_2$+2MoO_2+S	-4.58
$PtTe_2$+2MoO_2+2S	-7.30
$PtTe_2$+2MoO	-1.32
$PtTe_2$+2MoO+S	-6.95
$PtTe_2$+2MoO+2S	-10.63
$PtTe_2$+2MoO+3S	-15.04
$PtTe_2$+2Mo	0.71
$PtTe_2$+2Mo+S	-6.18
$PtTe_2$+2Mo+2S	-9.47
$PtTe_2$+2Mo+3S	-15.46
$PtTe_2$+2Mo+4S (2MoS_2)	-19.49

Supplementary Table 3. Comparison of R_c values for 3D metal/vdW metal interfacial system.

2D metals	3D metal contact	Deposition method	Preparation methods of 2D metals	R_c (kΩ·μm)	Ref.
**1T-$PtTe_2$**	**Ti/Au**	**E-beam evaporator**	**Powder-based tellurization**	**0.23 ± 0.02**	**This study**
**$MoTe_2$ (1T' phase)**	Au	N/A	CVD	0.95	Ref. 55
	Ti/Au	E-beam evaporator	CVD	0.47	Ref. 56
	Pd/Au	E-beam evaporator	CVD	0.6	Ref. 54
**WTe_2**	Ti/Au	E-beam evaporator	Exfoliated	~0.4-0.9	Ref. 22
**Graphene**	Cr/Au	E-beam evaporator	CVD	90	Ref. 57
	Ni			12	
	Pd			3	
	Ti/Au	E-beam evaporator	CVD	3	Ref. 18
	Cu			3.5	
	Pd			2.5	
	Ti/Au	E-beam evaporator	Exfoliated	1.65	Ref. 58
	Ti/Au	E-beam evaporator	Exfoliated	$5\text{-}10^2$	Ref. 59
		Sputtering		$10^6\text{-}10^7$	

Supplementary Table 4. Comparison of preparation methods for obtaining 2D or 3D MSJs based on MoS_2.

2D- or 3D-edge	Contact	Preparation methods		Patterning of heterojunction	Pure edge contact	Extraction method for R_c	Multiple calculations of R_c	Ref.
		2D or 3D metal	2D MoS_2					
2D-edge	**$PtTe_2$**	**CVD**	**CVD**	**Yes**	**Yes**	**TLM and Y-function**	**Yes**	**This study**
	Mo_2C	Mechanical exfoliation	CVD	No	Yes	Four-point	No	Ref. 16
	Graphene	Transfer of CVD-grown layer	CVD	Yes	No	Four-point	No	Ref. 51
			CVD	Yes	No	N/A		Ref. 38
			CVD	Yes	No	Four-point	No	Ref. 39
			CVD	Yes	No	Y-function	No	Ref. 40
			CVD	No	Yes	N/A		Ref. 41
	VS_2 (Degraded)	CVD	CVD	Yes	No	Four-point	No	Ref. 42
3D-edge	Ti	3D metal deposition	Mechanical exfoliation	No	Yes	N/A		Ref. 35
	Ni		CVD	Yes	Yes	TLM	Yes	Ref. 43
	Ti/Au		Mechanical exfoliation	Yes	Yes	Y-function	Yes	Ref. 44
	Mn		Mechanical exfoliation	Yes	Yes	Four-point	No	Ref. 23
	Sc		Mechanical exfoliation	No	Yes	Four-point	No	Ref. 37
	Au		CVD	No	Yes	Four-point	No	Ref. 24

Supplementary Table 5. Device performances of few-layered *n*-type MoS_2-based FETs with a short L_T (~0.65-80 nm). The values here are the best reported in each paper, whereas the average value from multiple devices is provided in the parentheses. Note that the graphene lateral contacts[38-40,51] have some vertical overlapping between the materials, which suggests that there may not be exact edge contact, where L_T is longer than the thickness of the channel (starred). Fair comparison of the R_c values is not possible due to the varying extraction methods. For example, a four-point measurement can cause the current shunting effect, resulting in underestimated resistance values[47]. For our $PtTe_2$ multilayer flake-based MoS_2 MSJ, the device geometry made the extraction of R_c by TLM or four-point measurement challenging. Instead, we estimated that R_c for our device would be less than ~125 kΩ·μm, which could be achieved via the relation of $R_{total} \approx 2R_c$ at high V_g. As high V_g values can substantially lower R_{sh}, we can neglect the R_{ch} term in the equation: $R_{total} = R_{ch} + 2R_c$. '1L' signifies the monolayer of MoS_2. The represented μ_{FE} is the two-terminal field-effect mobility of electrons and not the Y-function-extracted intrinsic mobility or four-terminal field-effect mobility.

**2D- or 3D-edge**	**Contact**	***H* of MoS_2**	***L* of MoS_2 (μm)**	**L_T (nm)**	**μ_{FE} ($cm^2V^{-1}s^{-1}$)**	**On/off ratio**	**SBH at V_{FB} (meV)**	**R_c at high V_g (kΩ·μm)**	**n_{2D} (10^{12} cm^{-2})**	**Extraction method for R_c**	**Gate dielectric**	**Ref.**
2D-lateral	**$PtTe_2$ flake**	**1L**	**0.5**	**0.65**	**< 16.2**	**10^6**	**35.9 ± 9.8**	**< 125**	**N/A**	**a relation of $R \approx 2R_c$**	**300 nm SiO_2**	**This study**
	**$PtTe_2$ film**		**> 10**		**< 17.9** (10.6 ± 2.9)	**10^7-10^8**	**N/A**	**> 168** (168 ± 127)	**9**	**TLM**	**50 nm Al_2O_3**	
								**> 18.2** (113.0 ± 60.3)	**N/A**	**Y-function**		
	Mo_2C	Few-layer	2	N/A	N/A	10^6	26	1.2	2	Four-point	285 nm SiO_2	Ref. 16
	Gr.	1L	2	2.5*	N/A	10^6	40	300	N/A	Four-point	300 nm SiO_2	Ref. 51
			2	2-30*	11.5	10^5	30-50	N/A			SiO_2	Ref. 38
			2.5	~80*	30 (17.2 ± 3.9)	10^6	N/A	11	6	Four-point	20 nm ZrO_2 (top gate)	Ref. 39
			20	10*	8.5 (5)	10^9	N/A	435	N/A	Y-function	20 nm Al_2O_3 (top gate)	Ref. 40
			1	N/A	18	10^8	N/A				300 nm SiO_2	Ref. 41
	VS_2	> 2L	2	N/A	35	10^6	25-45	0.52	6	Four-point	285 nm SiO_2	Ref. 42

3D-edge	Ti	59 nm	8	59	15	10^5-10^6	140	N/A			285 nm SiO_2	Ref. 35
	Mo	21 nm	6	21	N/A	10^3-10^4	70	N/A				
	Cr	3L	2.2	1.95	N/A	10^3-10^4	11.7	N/A			300 nm SiO_2	Ref. 43
	Ni	1L	0.6	0.65	N/A	10^4-10^5	N/A	30.5	12	TLM		
		2L	0.3	1.3	N/A			30.5	12	TLM		
	Ti/Au	1L	1	0.65	N/A	10^7	N/A	64.2	N/A	Y-function	BN/SiO_2	Ref. 44
	Mn	4.6 nm	N/A	4.6	15	10^5-10^6	N/A	500	N/A	Four-point	BN/SiO_2	Ref. 23
	Sc	1L	1.8	0.65	1.2 (1.1 ± 0.1)	10^4-10^5	N/A				BN/SiO_2	Ref. 37
		2L	N/A	1.3	N/A			943.5	4	Four-point		
		11L	2.4	7	9 (4.2)	10^1	N/A					
	Au	1L	12	0.65	5	10^4-10^5	124	990	N/A	Four-point	BN/SiO_2	Ref. 24

Supplementary Note 1. Edge-mediated growth of monolayer MoS_2

To obtain monolayer MoS_2, a high reaction rate at low mass flux is generally required in CVD-based synthesis of 2D TMDs[60]. In our experiments, we controlled the growth aspects by using (i) a confined space for gas precursors and a MoO_x thin-film precursor, (ii) a lateral growth promoter (i.e., NaCl), and (iii) a reduced growth temperature of 700 °C.

We placed the target ($PtTe_2/SiO_2/Si$) and precursor ($MoO_x/SiO_2/Si$) substrates in a face-to-face configuration to create a confined area between the two substrates. This resulted in a reduction of the S precursor concentration ($C(x,t)$) inside the area as a function of the distance from the edge of the target substrate (x) given by

$$C(x,t) = \frac{C_s}{\sqrt{\pi D_t}} \exp\left(-\frac{x^2}{4Dt}\right) \quad \text{(S10)}$$

where C_s is the concentration of the gas precursor arriving at the edge, D is the diffusion coefficient, and t is time. The decay of the mass flux ($J \propto C$) with increasing x therefore prevented excessive S flux inside the confined region[61]. At the same time, the use of a MoO_x thin-film precursor instead of powder precursor enabled the clean and reproducible growth of monolayer MoS_2 because the amount of MoO_x precursor required could be decreased by controlling the deposited thickness precisely[62,63]. The small fluxes of Mo and S significantly reduced the supersaturation level near the substrates, resulting in a very low nucleation density and an enlarged monolayer domain. They also prevented the formation of multilayers and undesired phases such as MoO_xS_y[63]. Using a similar strategy, Mohapatra *et al.*[61] successfully grew monolayer MoS_2 along pre-patterned Au arrays through gas-confined CVD. In contrast, the challenges in managing the local vapor pressure of S and Mo in typical powder-based CVD result in the formation of multilayers and intermediate products.

To promote a high reaction rate for the formation of large-sized monolayer MoS_2, we used a NaCl promoter coated on the corner of the MoO_x precursor film. The use of alkali metal compounds resulted in the formation of alkali metal molybdates and molybdenum oxide or oxychloride compounds that transformed into monolayer MoS_2 during growth. The low melting point of the intermediates increased their mobility. This reduced the number of nuclei and resulted in the preferential lateral synthesis of MoS_2 at 650–700 °C[60,64].

We further maintained the growth temperature at a reduced temperature of 650–700 °C instead of the usual 800 °C for edge-mediated growth. Previous studies on the CVD of 2D-2D lateral heterostructures[40,65] have shown that edge-mediated synthesis is reliant on the kinetic effect rather than the thermodynamic mechanism, which is predominantly dependent on the growth temperature. A high temperature (nearly 800 °C) promotes the extensive nucleation and growth of 2D semiconductors and leads to a thermodynamic preference for vertical heterostructures. In contrast, lowering the growth temperature (<750 °C) promoted edge-mediated synthesis, which led to the formation of in-plane heterojunctions via a kinetic effect rather than a thermodynamic mechanism.

Supplementary Note 2. Device operation principle

(i) *Schottky barrier formation*

Because 2D materials are thin and vulnerable to defects when typical metal electrodes are deposited onto their surfaces, process-induced defects can, in general, produce a high density of interfacial defects at the MSJ. When a large number of interfacial defects are present, the SBH becomes independent of the metal WF and is predominantly determined by the mid-gap states, as described in the following equation:

$$SBH = S(WF - E_{\mathrm{CNL}}) + (E_{\mathrm{CNL}} - \chi). \qquad \text{(S11)},$$

where S (= |d(SBH)/d(WF)|) is the pinning factor, which can be statistically evaluated by plotting the SBHs of semiconductors with different metal-contact WFs. Assuming a model for the surface states and barrier height, the S value is also a function of the interface trap density (D_{it})[66-68] as follows:

$$S = \frac{\varepsilon_i}{\varepsilon_i + q^2 D_{it}\delta}. \qquad \text{(S12)}$$

In this model, an interfacial layer with thickness (δ) of a few angstroms and small interfacial permittivity (ε_i), which allows electrons with energies greater than the potential barrier to pass, was assumed. The layer is assumed to be transparent to electrons with energies greater than the potential barrier. Because the δ and ε_i values denote interfacial properties, they are affected by the surfaces of the metal contact and semiconductor. Accordingly, the analysis by S is a universal method to analyze the FLP for many 2D semiconductor FETs with different metal surfaces/interfaces. For example, the extraction of the S value has been frequently conducted for many vertical 3D/2D MSJ, in which the high-energy deposition of 3D metal results in the formation of an atomic metal extrusion surrounded by a 2D semiconductor[5, 47] (i.e., long δ). The calculation of S was also conducted for edge contacts with a 2D semiconductor[49,51], which provided a better understanding of the edge interfacial properties. When D_{it} ($\approx \infty$) is large enough at the MSJ interface, the SBH is described by the Bardeen limit of strong pinning, which is expressed as $S = 0$ ($SBH = E_{\mathrm{CNL}} - \chi$), whereas the Schottky–Mott limit ($S = 1$; $SBH = WF - \chi$) is recovered when there are minimal interfacial defects ($D_{\mathrm{it}} \approx 0$). Therefore, the value of D_{it} can provide a better understanding of the effect of interfacial defects in the SBH formation mechanism. For instance, assuming that δ = 1 nm (ref. [5]), D_{it} at the 3D metal/MoS_2 interface is ~2.68×10^{14} eV^{-1} cm^{-2}. This is almost ten times larger than the D_{it} of a typical Si-based MSJ (~2.7×10^{13} eV^{-1} cm^{-2}) and induces stronger FLP in the 2D MoS_2 (ref. [67]).

This surface model implies that the edge-contact MSJ has a significant advantage because it possesses a weakened FLP effect compared to defect-rich conventional 3D metal contacts due to the atomically thin δ and smaller D_{it}. Because the edge contact has a 1D-like interface, the value of δ should correspond to the size of a single atom in principle. The suppression of process-induced defects (e.g., nonstoichiometric alloying, phase separation, and crystal discontinuity) leads to smaller values of D_{it}. Negligible δ and D_{it} values can lead to a weaker FLP because S can approach ~1. Through XPS and TEM analyses, we observed minimal δ and D_{it} values in our $PtTe_2$-MoS_2 MSJ FETs. The epitaxially grown MoS_2 at the

$PtTe_2$ edge did not exhibit substantial variations in its binding energies and atomic structure compared to bare MoS_2 flakes. Furthermore, the small subthreshold swing (SS) of the MoS_2 FETs with $PtTe_2$ contacts suggests that they had a smaller D_{it} value compared to the FETs with Ti 3D metal contacts. The SS values are typically determined using the semiconductor capacitance (C_s), dielectric capacitance (C_{ox}), and capacitance from interfacial charges at the MoS_2/oxide interface ($C_{it,ox}$) as follows:

$$SS = \frac{dV_g}{d(\log_{10} I_{ds})} = \ln 10 \cdot \frac{k_B T}{q}\left(1 + \frac{C_s + C_{it,ox} + C_{it}}{C_{ox}}\right) \approx 60\ \text{mV}\left(1 + \frac{C_{it,ox} + C_{it}}{C_{ox}}\right). \quad \text{(S13)}$$

Because the C_s, C_{ox}, and $C_{it,ox}$ values were the same in our FETs regardless of whether they contained edge or vertical contacts, the differences in the SS values between the devices were solely due to their contact properties. For example, a parasitic capacitance (C_{it}) in parallel to a contact resistance (R_c) can be introduced into a dielectric/MoS_2/contact system[69-71].The minimum *SS* value for the MoS_2 FET with a $PtTe_2$ contact (~0.38 V/dec) was smaller than that for Ti/MoS_2 (~1.01 V/dec), indicating that the edge-contact $PtTe_2$ had a reduced value of D_{it} and exhibited weaker FLP behavior at the interfaces compared to the vertical 3D Ti metal contact. Based on this model, we estimated $D_{it,ox}+D_{it} \approx 5.73 \times 10^{12}$ eV^{-1} cm^{-2} for the $PtTe_2$-MoS_2 MSJ, whereas it increased to ~1.70×10^{13} eV^{-1} cm^{-2} for Ti/MoS_2. Because the $C_{it,ox}$ values are comparable across the two contact geometries, the increase in D_{it} (ΔD_{it}) due to the use of the top contact in place of the edge contact is ~1.13×10^{13} eV^{-1} cm^{-2}.

(ii) *Tunneling barrier formation*

Another obstacle to carrier transport in the conventional 3D top-contact geometry is the presence of an extra tunneling barrier (TB) between the 2D semiconductor and 3D metal. The vdW gap at the interface has a notable contribution to this TB. Because the vdW interface has no significant orbital overlap at the interface, the resultant formation of a square tunnelling decreases the barrier field emission efficiency. According to various studies[4,72], the quantum tunnelling probability (P_{TB}) through the vdW gap can be calculated as

$$P_{TB} = \exp\left(-\frac{2T_{vdw}}{\hbar}\sqrt{2m\Phi_{TB}}\right) \quad \text{(S14)}$$

where $\hbar$ is the reduced Planck's contact, m the free electron mass, Φ_{TB} the tunneling barrier height, and T_{vdw} the width of the TB. Equation S14 implies that a lower barrier height and narrower width are required for higher electron injection efficiency during the tunneling process. Quantitative analysis using computational methods suggests that the values of Φ_{TB} and T_{vdw} span across a diverse range depending on the atomic size and orbital overlaps of the metal. Although the Ti/MoS_2 MSJ had a smaller Φ_{TB} and T_{vdw} than those of the In, Au, and Pd interface[72], the existence of the square TB affected carrier injection through the interface, especially when compared to the edge contact. Therefore, in the top-contact MoS_2 FET with the extra TB, electrons were mostly injected through thermionic emission over the barrier. In contrast, the edge contact allowed additional injection into the channel via field and thermionic field emission due to the absence of the TB and increased orbital overlap.

(iii) *Carrier transport behavior through the barriers*

We illustrate the effects of the SB and TB on V_g-dependent carrier transport in top-contact Ti/MoS_2 and edge-contact $PtTe_2$-MoS_2 MSJ FETs in Supplementary Fig. 19. The thermionic emission model was fitted to Figure 2i in the manuscript under the assumption that the injection current was dominated by thermionic emission carriers. This assumption is valid when $V_g < V_{FB}$, in which case, the extracted Φ_B is the exact value of the effective barrier height and Φ_B at V_{FB} is the true SBH of the interface. Consequently, in the Ti/MoS_2 MSJ, which had a high SBH due to a large FLP, the influence of the SB resulted in Φ_B values that were more than twice that of the $PtTe_2$-MoS_2 MSJ.

In contrast, when $V_g > V_{FB}$, thermionic tunneling current begins to contribute because the SBW is narrowed by band bending. Thus, in this on-state bias regime, the influence of the BW (including T_{vdw}) is mainly reflected mainly in the Φ_B-V_g curve (Fig. 2i). The SBW values at $V_g = V_{FB}$ for our MoS_2 with Ti (~1.5 nm) and $PtTe_2$ contacts (~0.94 nm) could be calculated by using Equation S2 in the Supplementary Information. The reduced SBW of the edge contact resulted in a smaller Φ_B that could even approach ~0 meV in Fig. 2i owing to the substantial band bending. On the other hand, the Φ_B of Ti had finite values at all V_g values, which could be attributed to the considerable impact of the SBW and T_{vdW}.

Supplementary Note 3. Essential considerations for fair performance evaluations

(i) *Field-effect mobility*

To determine the field-effect mobility (μ_{FE}), we considered the peak transconductance (g_m = dI_{ds}/dV_g) at the maximum slope of the I_{ds}-V_g curve. This is the most frequently used technique. The field-effect mobility is then given by

$$\mu_{FE} = \frac{L}{WC_gV_{ds}} g_m. \qquad \text{(S15)}$$

Although this approach is universally applicable, different edge-contact MSJ FET fabrication processes can result in different intrinsic, extrinsic, and device geometry-related contact effects that lead to the overestimation or underestimation of μ_{FE}. In particular, the edge-contact 3D-2D MSJ was fabricated through *in situ* etching of 2D semiconductors followed by deposition of 3D metal, whereas the lateral 2D-2D MSJ was fabricated through CVD. The fundamental differences between the two processes led to discrepancies in both the channel quality and producible device structure. Therefore, to perform a fair comparison of μ_{FE} values across edge-contact MSJ FETs reported in different studies, the factors that can affect the performance parameter should be considered carefully. In particular, both the short-channel effect and intrinsic mobility (μ_0 instead of μ_{FE}) should be considered to avoid misinterpretation of μ_{FE}.

The μ_{FE} in short-channel devices tends to be underestimated compared to that in devices with longer L because R_{ch} scales with L whereas R_c is independent of L (ref. 73). In our $PtTe_2$-MoS_2 MSJ FETs, L was long enough (>10 μm) for this short-channel effect to be negligible. We compared the μ_{FE} values of lateral MSJ FETs from various studies as functions of L. Our study shows that μ_{FE} in two-terminal devices (up to ~17.9 and ~10.6 ± 2.9 $cm^2V^{-2}s^{-1}$ on average) are higher than those in edge-contact MSJ FETs with 3D metals, but slightly smaller than those in 2D-2D MSJ FETs considering their L. The higher μ_{FE} of the reported 2D-2D MSJ FETs could be explained by the higher carrier injection due to the vertical overlap of the interface between the 2D semiconductor and 2D metals (i.e., longer L_T), which was not the case in our devices (Supplementary Fig. 21b). We can hence conclude that our devices exhibited higher μ_{FE} values than any other pure edge contacts with sub-nanometer L_T.

The determination of the μ_0 value using the Y-function method should also be considered if the μ_{FE} of the device is severely underestimated. The Y-function method yields the μ_0 value without any R_c-related degradation and provides a good approach to understanding the intrinsic device properties. In our in-plane $PtTe_2$-MoS_2 MSJ, the μ_0 values were calculated to be ~11.1 ± 4.5 $cm^2V^{-2}s^{-1}$ (averaged over 30 devices), which is almost comparable to the μ_{FE} values (~10.6 ± 2.9 $cm^2V^{-2}s^{-1}$) calculated using g_m (see Supplementary Fig. 22 for μ_0 calculation details). The insignificant difference between the values (~4.5%) implies that there was only a minor contact barrier at the edge interface with little impact on μ_{FE}.

In addition to the above considerations, the miscalculation of μ_{FE} can also be caused by the choice of the V_g sweeping direction and the top-dielectric geometry; however, our devices were unaffected by these minor considerations. The dielectric interface, and in particular the V_g-induced interface traps there, can affect the hysteresis behavior of FETs, which may lead to overestimation of the g_m values during the backward V_g sweep[74]. This was avoided by using the forward sweep direction (i.e., negative to positive) to obtain the g_m of our devices. The

coupling between the top and bottom capacitances of the dielectric oxide in the device structure may also lead to overestimation of μ_{FE} (refs. [75,76]). It is likely that μ_{FE} has been overestimated in some previously reported devices with edge contacts and top-gated structures[39,40] because the gate capacitance was not accurately assessed. In contrast to these previous studies, our FETs were measured through the back-gated dielectric oxide (which suppressed dielectric coupling), and their actual capacitance was characterized through C-V analysis (see Methods).

Finally, we emphasize the importance of extracting μ_{FE} from multiple edge-contact FETs. This is particularly important for edge contacts considering the significant challenges in the reproducible fabrication of such devices. However, most of the reports on edge-contacted devices showed only one or two of the most promising data sets, which raises concerns for their technological feasibility. In our study, the high processability of $PtTe_2$ allowed the fabrication of more than 30 different FETs with edge contacts. Statistical calculations can therefore be performed on μ_0 and μ_{FE}.

(ii) *Contact resistance*

Similar to μ_{FE}, the contact resistance (R_c) is also frequently misestimated or misinterpreted. The most commonly used method to calculate R_c for edge-contact MSJ FETs is the four-point probe measurement method, which requires voltage-sensing probes to be added along the 2D channel to measure the voltage drop between the probes while I_{ds} is applied. The voltage-sensing probes should have small point-like contact areas with the channel to avoid perturbing the electric field[77]. The application of non-ideal voltage-sensing electrodes with large contact areas instead of small point-like contact areas to the channel leads to the considerable underestimation of the R_c value in four-point probe measurements because of the shunted current paths through the sensing probes. In particular, in 2D FETs, the practical constraints of both the patterning techniques and the chemical reactions of 3D metals with 2D semiconductors can result in a considerable invasion of the voltage probe contact area. According to a previous study, the R_c value obtained using the four-probe method in identical MoS_2 MSJs with conventional 3D vertical contacts may be underestimated by a factor of ten[7]. Most importantly, for 2D MSJ FETs with edge contacts, the voltage-sensing probe should take the form of edge contacts to avoid mixed effects from 3D vertical metal contacts[23]. However, this scheme was not utilized in nearly all the previous studies on lateral MSJ FETs[24,37,39,42,51]. It is therefore inappropriate to compare the R_c values in these studies with those obtained using other techniques, such as the Y-function method or TLM.

The extraction of R_c using the Y-function method requires only a linear regime in the I_{ds}-V_g transfer curve. This provides an advantage over the four-point probe measurement method and TLM because it avoids a number of issues related to variations across different channels and the reproducibility of fabricating FETs with ultrathin edge interfaces. However, the Y-function method is only applicable to a FET in which V_g has no effect on the source or drain, i.e., a device operating in the strong accumulation regime[45]. Furthermore, in contrast to the assumption that R_c is independent of V_g, R_c in actuality gradually decreases with the injected carrier density and slowly saturates towards the accumulation regime in conventional MSJ FETs. Therefore, the Y-function technique provides improved measurements only when the impact of the Schottky barrier (SB) is minimal[44,78]. This method is therefore appropriate for edge-contact MSJs with small SBH and SB widths.

Compared to the four-point probe measurement and Y-function methods, the TLM provides more accurate measurements of R_c at different values of V_g although it requires multiple FETs with different channel L. Despite its accuracy, the TLM is an extrapolation method with the important requirement that all the R_c and channel R_{sh} values must be consistent across metal probes. The satisfaction of this requirement in previous studies on edge contacts was challenging because of fabrication complexity and/or device-to-device variations. In contrast, the TLM patterns in our $PtTe_2$-MoS_2 MSJ FETs enabled the extraction of R_c (168 ± 127 kΩ·µm). This demonstrates their possibilities in large-scale-compatible technology and spatial control of the coplanar MS interface. The consistency of the measurements is demonstrated by the agreement of the R_c values with the average values extracted using the Y-function method (113 ± 60 kΩ·µm). The agreement of the R_c values measured over 30 devices using the two distinct methods in Fig. 4g indicates that there were minor measurement errors and substantial variance between the FETs. It should also be noted that other studies on edge contacts with 2D MoS_2 typically reported only the best value obtained from four-probe point measurements[16,23,24,37,39,42,51], which should be avoided for appropriate benchmarking.

For edge or lateral contacts with ultrashort L_T (= L_c), the specific contact resistivities (ρ_c) rather than R_c should be evaluated and compared. This is because the R_c value is primarily affected by the thickness of the edge interface (i.e., $\rho_c = R_c/L_T$) owing to the surge in current crowding with the reduction of L_T. Considering the potential applications of edge contacts as ultra-scaled contact electrodes, the achievement of low values for both ρ_c and L_T is desirable in 2D FETs. In Figure 4h, we compare the ρ_c and L_T values of MoS_2-based MSJ FETs with edge and top contacts. The values for the $PtTe_2$-MoS_2 MSJ FET were almost the same as the lowest values reported for edge contacts (ρ_c as low as ~11.7 $\Omega \cdot \mu m^2$ and L_T of ~0.7 nm), which is promising for the realization of ultralow R_c in L_c-scale FETs. We also compared ρ_c as a function of n_{2D} for a fair comparison in Supplementary Fig. 21c because the V_g-induced carrier densities (n_{2D}) in a 2D channel can vary across different papers. Our $PtTe_2$ edge contact showed the lowest ρ_c across different n_{2D} values.

Regarding the thickness of the MoS_2 channel, multilayer MoS_2 (the case for refs.[5,13-15]) could have SBH and R_c values smaller than a monolayer because of less influence of interfacial traps at the substrates[7] and the downshift of the conduction band edge in thicker MoS_2[29].

Supplementary References

1 Liu, Y., Stradins, P. & Wei, S.-H. Van der Waals metal-semiconductor junction: Weak Fermi level pinning enables effective tuning of Schottky barrier. *Sci. Adv.* **2**, e1600069 (2016).

2 Majidi, L. *et al.* New Class of Electrocatalysts Based on 2D Transition Metal Dichalcogenides in Ionic Liquid. *Adv. Mater.* **31**, 1804453 (2019).

3 Shawkat, M. S. *et al.* Large-area 2D $PtTe_2$/silicon vertical-junction devices with ultrafast and high-sensitivity photodetection and photovoltaic enhancement by integrating water droplets. *Nanoscale* **12**, 23116-23124 (2020).

4 Shen, T., Ren, J.-C., Liu, X., Li, S. & Liu, W. van der Waals Stacking Induced Transition from Schottky to Ohmic Contacts: 2D Metals on Multilayer InSe. *J. Am. Chem Soc.* **141**, 3110-3115 (2019).

5 Kim, C. *et al.* Fermi Level Pinning at Electrical Metal Contacts of Monolayer Molybdenum Dichalcogenides. *ACS Nano* **11**, 1588-1596 (2017).

6 Hu, X. *et al.* Infrared Nanoimaging of Surface Plasmons in Type-II Dirac Semimetal $PtTe_2$ Nanoribbons. *ACS Nano* **14**, 6276-6284 (2020).

7 Politano, A. *et al.* Tailoring the Surface Chemical Reactivity of Transition-Metal Dichalcogenide $PtTe_2$ Crystals. *Adv. Funct. Mater.* **28**, 1706504 (2018).

8 Kim, B. G., Lee, J.-H. & Choi, S.-M. Selective decoration of nanocrystals on single-crystalline PtTe nanowires based on a solid-state reaction. *RSC Adv.* **5**, 80766-80771 (2015).

9 Wang, M. *et al.* Wafer-Scale Growth of 2D $PtTe_2$ with Layer Orientation Tunable High Electrical Conductivity and Superior Hydrophobicity. *ACS Appl. Mater. Interfaces* **12**, 10839-10851 (2020).

10 Zhang, K. *et al.* Growth of large scale PtTe, $PtTe_2$ and $PtSe_2$ films on a wide range of substrates. *Nano Res.* **14**, 1663-1667 (2021).

11 Schaeffer, J. *et al.* Contributions to the effective work function of platinum on hafnium dioxide. *Appl. Phy. Lett.* **85**, 1826-1828 (2004).

12 Anemone, G. *et al.* Experimental determination of surface thermal expansion and electron–phonon coupling constant of 1T-$PtTe_2$. *2D Mater.* **7**, 025007 (2020).

13 Wang, H. *et al.* Electrochemical tuning of vertically aligned MoS_2 nanofilms and its application in improving hydrogen evolution reaction. *Proc. Natl. Acad. Sci. U.S.A.* **110**, 19701-19706 (2013).

14 Li, Y. *et al.* Au@MoS_2 Core–Shell Heterostructures with Strong Light–Matter Interactions. *Nano Lett.* **16**, 7696-7702 (2016).

15 Cain, J. D., Shi, F., Wu, J. & Dravid, V. P. Growth Mechanism of Transition Metal Dichalcogenide Monolayers: The Role of Self-Seeding Fullerene Nuclei. *ACS Nano* **10**, 5440-5445 (2016).

16 Jeon, J. *et al.* Epitaxial Synthesis of Molybdenum Carbide and Formation of a Mo_2C/MoS_2 Hybrid Structure via Chemical Conversion of Molybdenum Disulfide. *ACS Nano* **12**, 338-346 (2018).

17 Xia, F., Perebeinos, V., Lin, Y.-m., Wu, Y. & Avouris, P. The origins and limits of metal–graphene junction resistance. *Nat. Nanotech.* **6**, 179-184 (2011).

18 Park, H.-Y. *et al.* Extremely Low Contact Resistance on Graphene through n-Type Doping and Edge Contact Design. *Adv. Mater.* **28**, 864-870 (2016).

19 Liu, F., Navaraj, W. T., Yogeswaran, N., Gregory, D. H. & Dahiya, R. van der Waals Contact Engineering of Graphene Field-Effect Transistors for Large-Area Flexible Electronics. *ACS Nano* **13**, 3257-3268 (2019).

20 Yan, M. *et al.* Lorentz-violating type-II Dirac fermions in transition metal dichalcogenide $PtTe_2$. *Nat. Commun.* **8**, 257 (2017).

21 Hao, S. *et al.* Low-Temperature Eutectic Synthesis of $PtTe_2$ with Weak Antilocalization and Controlled Layer Thinning. *Adv. Funct. Mater.* **28**, 1803746 (2018).

22 Mleczko, M. J. *et al.* High Current Density and Low Thermal Conductivity of Atomically Thin Semimetallic WTe_2. *ACS Nano* **10**, 7507-7514 (2016).

23 Choi, H. *et al.* Edge Contact for Carrier Injection and Transport in MoS_2 Field-Effect Transistors. *ACS Nano* **13**, 13169-13175 (2019).

24 Moon, B. H. *et al.* Junction-Structure-Dependent Schottky Barrier Inhomogeneity and Device Ideality of Monolayer MoS_2 Field-Effect Transistors. *ACS Appl. Mater. Interfaces* **9**, 11240-11246 (2017).

25 Tung, R. T. Recent advances in Schottky barrier concepts. *Mater. Sci. Eng. R Rep.* **35**, 1-138 (2001).

26 Tersoff, J. Schottky Barrier Heights and the Continuum of Gap States. *Phys. Rev. Lett.* **52**, 465-468 (1984).

27 Léonard, F. & Tersoff, J. Role of Fermi-Level Pinning in Nanotube Schottky Diodes. *Phys. Rev. Lett.* **84**, 4693-4696 (2000).

28 Dong, H. *et al.* Schottky Barrier Height of Pd/MoS_2 Contact by Large Area Photoemission Spectroscopy. *ACS Appl. Mater. Interfaces* **9**, 38977-38983 (2017).

29 Kim, G.-S. *et al.* Schottky Barrier Height Engineering for Electrical Contacts of Multilayered MoS_2 Transistors with Reduction of Metal-Induced Gap States. *ACS Nano* **12**, 6292-6300 (2018).

30 Agrawal, A., Shukla, N., Ahmed, K. & Datta, S. A unified model for insulator selection to form ultra-low resistivity metal-insulator-semiconductor contacts to n-Si, n-Ge, and n-InGaAs. *Appl. Phys. Lett.* **101**, 042108 (2012).

31 Chen, X. *et al.* Probing the electron states and metal-insulator transition mechanisms in molybdenum disulphide vertical heterostructures. *Nat. Commun.* **6**, 6088 (2015).

32 Cho, K. *et al.* Electrical and Optical Characterization of MoS_2 with Sulfur Vacancy

Passivation by Treatment with Alkanethiol Molecules. *ACS Nano* **9**, 8044-8053 (2015).

33 Dolui, K., Rungger, I. & Sanvito, S. Origin of the n-type and p-type conductivity of MoS_2 monolayers on a SiO_2 substrate. *Phys. Rev. B* **87**, 165402 (2013).

34 Yu, H., Gupta, S., Kutana, A. & Yakobson, B. I. Dimensionality-Reduced Fermi Level Pinning in Coplanar 2D Heterojunctions. *J. Phys. Chem.* **12**, 4299-4305 (2021).

35 Yang, Z. *et al.* A Fermi-Level-Pinning-Free 1D Electrical Contact at the Intrinsic 2D MoS_2–Metal Junction. *Adv. Mater.* **31**, 1808231 (2019).

36 Paz, W. S. & Palacios, J. J. A theoretical study of the electrical contact between metallic and semiconducting phases in monolayer MoS_2. *2D Materials* **4**, 015014 (2016).

37 Chai, Y. *et al.* Making one-dimensional electrical contacts to molybdenum disulfide-based heterostructures through plasma etching. *Phys. Status Solidi A* **213**, 1358-1364 (2016).

38 Behranginia, A. *et al.* Direct Growth of High Mobility and Low-Noise Lateral MoS_2–Graphene Heterostructure Electronics. *Small* **13**, 1604301 (2017).

39 Zhao, M. *et al.* Large-scale chemical assembly of atomically thin transistors and circuits. *Nat. Nanotech.* **11**, 954-959 (2016).

40 Hong, W., Shim, G. W., Yang, S. Y., Jung, D. Y. & Choi, S.-Y. Improved Electrical Contact Properties of MoS_2-Graphene Lateral Heterostructure. *Adv. Funct. Mater.* **29**, 1807550 (2019).

41 Suenaga, K. *et al.* Surface-Mediated Aligned Growth of Monolayer MoS_2 and In-Plane Heterostructures with Graphene on Sapphire. *ACS Nano* **12**, 10032-10044 (2018).

42 Leong, W. S. *et al.* Synthetic Lateral Metal-Semiconductor Heterostructures of Transition Metal Disulfides. *J. Am. Chem. Soc.* **140**, 12354-12358 (2018).

43 Cheng, Z. *et al.* Immunity to Contact Scaling in MoS_2 Transistors Using in Situ Edge Contacts. *Nano Lett.* **19**, 5077-5085 (2019).

44 Jain, A. *et al.* One-Dimensional Edge Contacts to a Monolayer Semiconductor. *Nano Lett.* **19**, 6914-6923 (2019).

45 Chang, H.-Y., Zhu, W. & Akinwande, D. On the mobility and contact resistance evaluation for transistors based on MoS_2 or two-dimensional semiconducting atomic crystals. *App. Phys. Lett.* **104**, 113504 (2014).

46 Andrews, K., Bowman, A., Rijal, U., Chen, P.-Y. & Zhou, Z. Improved Contacts and Device Performance in MoS_2 Transistors Using a 2D Semiconductor Interlayer. *ACS Nano* **14**, 6232-6241 (2020).

47 English, C. D., Shine, G., Dorgan, V. E., Saraswat, K. C. & Pop, E. Improved Contacts to MoS_2 Transistors by Ultra-High Vacuum Metal Deposition. *Nano Lett.* **16**, 3824-3830 (2016).

48 Zhang, Z. *et al.* Epitaxial Growth of Two-Dimensional Metal–Semiconductor Transition-Metal Dichalcogenide Vertical Stacks (VSe_2/MX_2) and Their Band

Alignments. *ACS Nano* **13**, 885-893 (2019).

49 Wang, B. *et al.* Bifunctional NbS_2-Based Asymmetric Heterostructure for Lateral and Vertical Electronic Devices. *ACS Nano* **14**, 175-184 (2020).

50 Kim, S. Y., Kwak, J., Ciobanu, C. V. & Kwon, S. Y. Recent developments in controlled vapor-phase growth of 2D group 6 transition metal dichalcogenides. *Adv.Mater.* **31**, 1804939 (2019).

51 Ling, X. *et al.* Parallel Stitching of 2D Materials. *Adv. Mater.* **28**, 2322-2329 (2016).

52 Zhang, Y. *et al.* Edge-Epitaxial Growth of 2D NbS_2-WS_2 Lateral Metal-Semiconductor Heterostructures. *Adv. Mater.* **30**, 1803665 (2018).

53 Lee, C.-S. *et al.* Epitaxial van der Waals Contacts between Transition-Metal Dichalcogenide Monolayer Polymorphs. *Nano Lett.* **19**, 1814-1820 (2019).

54 Xu, X. *et al.* Scaling-up Atomically Thin Coplanar Semiconductor–Metal Circuitry via Phase Engineered Chemical Assembly. *Nano Lett.* **19**, 6845-6852 (2019).

55 Sung, J. H. *et al.* Coplanar semiconductor–metal circuitry defined on few-layer $MoTe_2$ via polymorphic heteroepitaxy. *Nat. Nanotech.* **12**, 1064-1070 (2017).

56 Ma, R. *et al.* $MoTe_2$ Lateral Homojunction Field-Effect Transistors Fabricated using Flux-Controlled Phase Engineering. *ACS Nano* **13**, 8035-8046 (2019).

57 Song, S. M., Park, J. K., Sul, O. J. & Cho, B. J. Determination of work function of graphene under a metal electrode and its role in contact resistance. *Nano Lett.* **12**, 3887-3892 (2012).

58 Russo, S., Craciun, M. F., Yamamoto, M., Morpurgo, A. F. & Tarucha, S. Contact resistance in graphene-based devices. *Phys. E. Low-Dimens. Syst. Nanostructures* **42**, 677-679 (2010).

59 Nagashio, K., Nishimura, T., Kita, K. & Toriumi, A. Contact resistivity and current flow path at metal/graphene contact. *Appl. Phys. Lett.* **97**, 143514 (2010).

60 Zhou, J. *et al.* A library of atomically thin metal chalcogenides. *Nature* **556**, 355-359 (2018).

61 Mohapatra, P. K., Ranganathan, K. & Ismach, A. Selective Area Growth and Transfer of High Optical Quality MoS_2 Layers. *Adv. Mater. Interfaces* **7**, 2001549 (2020).

62 Withanage, S. S. *et al.* Uniform Vapor-Pressure-Based Chemical Vapor Deposition Growth of MoS_2 Using MoO_3 Thin Film as a Precursor for Coevaporation. *ACS Omega* **3**, 18943-18949 (2018).

63 Lee, J. *et al.* Thermodynamically Stable Synthesis of Large-Scale and Highly Crystalline Transition Metal Dichalcogenide Monolayers and their Unipolar n–n Heterojunction Devices. *Adv. Mater.* **29**, 1702206 (2017).

64 Wang, P. *et al.* Mechanism of Alkali Metal Compound-Promoted Growth of Monolayer MoS_2: Eutectic Intermediates. *Chem. Mater.* **31**, 873-880 (2019).

65 Gong, Y. *et al.* Vertical and in-plane heterostructures from WS_2/MoS_2 monolayers. *Nat.*

Mater. **13**, 1135-1142 (2014).

66 Cowley, A. M. & Sze, S. M. Surface States and Barrier Height of Metal-Semiconductor Systems. *J. Appl. Phys.* **36**, 3212-3220 (1965).

67 Wang, Y. & Chhowalla, M. Making clean electrical contacts on 2D transition metal dichalcogenides. *Nat. Rev. Phys.* **4**, 101-112 (2022).

68 Sotthewes, K. *et al.* Universal Fermi-Level Pinning in Transition-Metal Dichalcogenides. *J. Phys. Chem. C* **123**, 5411-5420 (2019).

69 Jang, J. *et al.* Clean Interface Contact Using a ZnO Interlayer for Low-Contact-Resistance MoS_2 Transistors. *ACS Appl. Mater. Interfaces* **12**, 5031-5039 (2020).

70 Liu, F. *et al.* 2D Ruddlesden–Popper Perovskite Single Crystal Field-Effect Transistors. *Adv. Funct. Mater.* **31**, 2005662 (2021).

71 Kappera, R. *et al.* Phase-engineered low-resistance contacts for ultrathin MoS_2 transistors. *Nat. Mater.* **13**, 1128-1134 (2014).

72 Kang, J., Liu, W., Sarkar, D., Jena, D. & Banerjee, K. Computational Study of Metal Contacts to Monolayer Transition-Metal Dichalcogenide Semiconductors. *Phys. Rev. X* **4**, 031005 (2014).

73 Nasr, J. R., Schulman, D. S., Sebastian, A., Horn, M. W. & Das, S. Mobility Deception in Nanoscale Transistors: An Untold Contact Story. *Adv. Mater.* **31**, 1806020 (2019).

74 Liu, Y. *et al.* Promises and prospects of two-dimensional transistors. *Nature* **591**, 43-53 (2021).

75 Radisavljevic, B. & Kis, A. Mobility engineering and a metal–insulator transition in monolayer MoS_2. *Nat. Mater.* **12**, 815-820 (2013).

76 Fuhrer, M. S. & Hone, J. Measurement of mobility in dual-gated MoS_2 transistors. *Nat. Nanotech.* **8**, 146-147 (2013).

77 Choi, H. H. *et al.* Accurate Extraction of Charge Carrier Mobility in 4-Probe Field-Effect Transistors. *Adv. Funct. Mater.* **28**, 1707105 (2018).

78 Hemanjaneyulu, K., Kumar, J. & Shrivastava, M. Gaps in the Y-Function Method For Contact Resistance Extraction in 2D Few-Layer Transition Metal Dichalcogenide Back-Gated FETs. *IEEE Electron Device Lett.* **43**, 635-638 (2022).
